# Straightforward Synthesis of Bifunctional Phosphorus Phenols via Phosphination of In Situ Generated *o*-Quinone Methides

**DOI:** 10.3390/molecules23061240

**Published:** 2018-05-23

**Authors:** Zhangpei Chen, Qinglong Shi, Gongshu Wang, Siwen Chen, Jianshe Hu

**Affiliations:** Center for Molecular Science and Engineering, College of Sciences, Northeastern University, Shenyang 110819, China; 1600210@stu.neu.edu.cn (Q.S.); 1770058@stu.neu.edu.cn (G.W.); 1770035@stu.neu.edu.cn (S.C.)

**Keywords:** *o*-quinone methides, 2-tosylalkyl phenols, phosphorus phenols, diphenylphosphine oxide, organophosphorus compounds

## Abstract

An efficient and practical approach towards bifunctional phosphorus phenols has been developed through a reaction of diphenylphosphine oxide and the *o*-quinone methides in situ generated from 2-tosylalkyl phenols under basic conditions. This protocol features simple experimental procedures under mild conditions and is easily scaled up. With this method, a variety of diarylmethyl phosphine oxides can be produced with up to 92% yield.

## 1. Introduction

**Figure 1:**
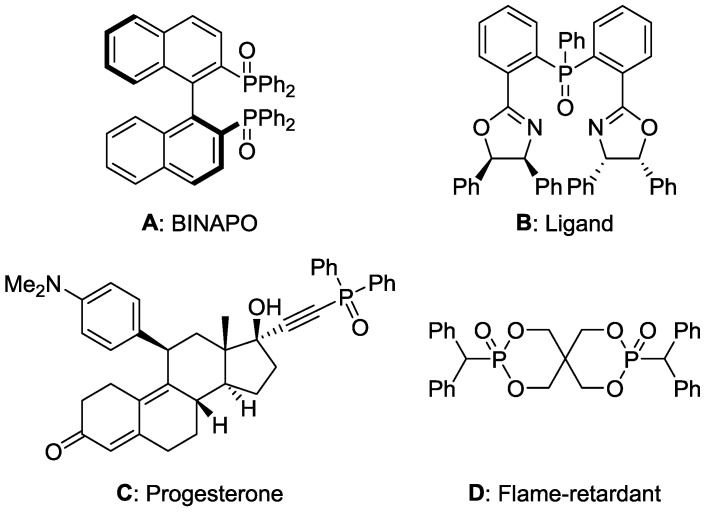
Some organophosphorus compounds.

Since the pioneering work of Wittig, organophosphorus compounds have emerged to be enormously important for various areas in chemistry and resonate across numerous disciplines [1,2,3,4]. Among them, phosphine oxides have attracted considerable attention due to their wide structural diversity and properties. In recent years, an increasing number of documents on the application of phosphine oxides in ligand chemistry [5,6], pharmaceutical chemistry [7,8,9], organic synthetic chemistry, and materials science have been demonstrated [10,11]. In particular, aside from the well-known triphenylphosphine oxide, plenty of novel phosphine oxides have been designed and synthesized as useful molecules for both industry and academia. For example, the chiral phosphine oxide compound **A** (BINAPO) [12] and the bisoxazoline phosphorus ligand **B** [13] have been employed as a catalyst or ligand to catalyze versatile asymmetric reactions; the phosphorus chromones **C** is a progesterone receptor antagonist [14]; compound **D** is known as a preeminent flame retardant [15] (Figure 1).

**Scheme 1:**
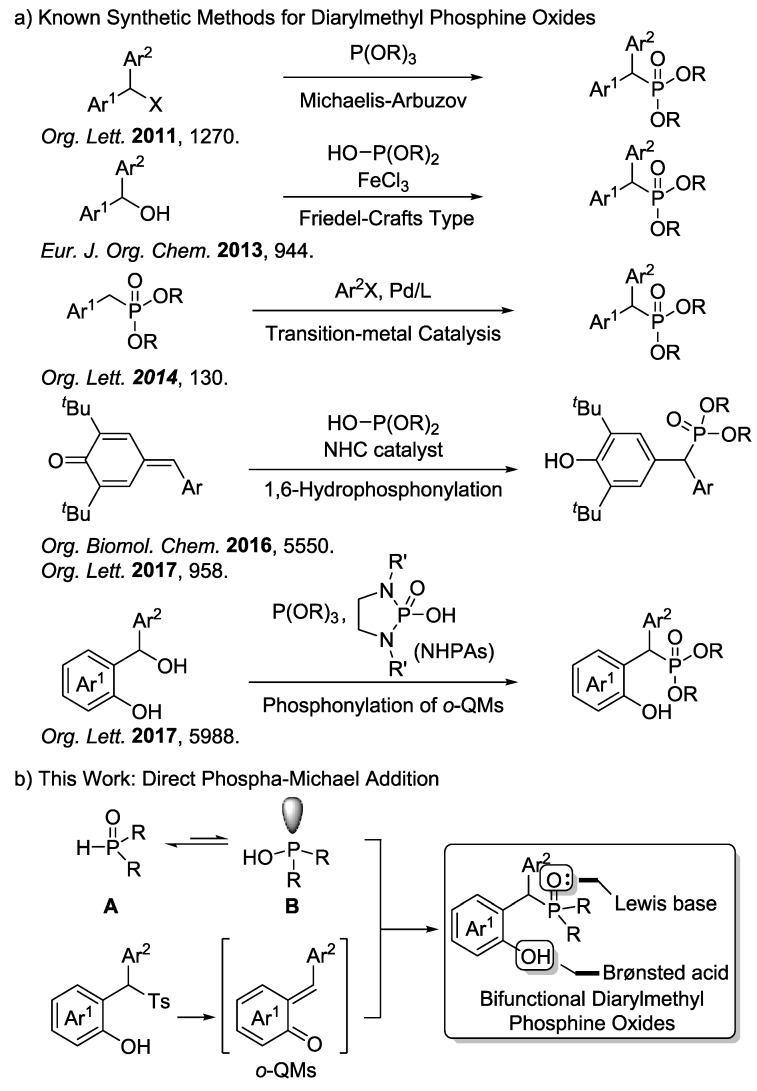
Synthesis of diarylmethyl phosphine oxides.

Accordingly, the preponderance of phosphine oxides has expedited the exploration of efficient and environmentally benign synthetic methods for their preparation [16,17,18,19,20]. Although much progress has been achieved in this chemistry, only a few approaches have been developed for the synthesis of arylated-methyl phosphine oxides, particularly diaryl and triarylmethyl phosphine oxides. Some classical approaches include the Michaelis−Arbuzov or Michaelis−Becker reaction [21,22] and the FeCl_3_-mediated Friedel−Crafts reaction [23] (Scheme 1a). However, these reactions require harsh reaction conditions and are limited to a narrow substrate scope. Walsh’s group developed the Pd-catalyzed α-arylation of benzylic phosphine oxides with haloarenes in medium to good yield [24]. Although this method involves a relatively wide substrate scope, the use of expensive metal catalysts and difficulty of scaling up make it unattractive. Recently, the Anand [25] and Kang groups [26] revealed 1,6-hydrophosphonylation of *para*-quinone methides (*p*-QMs) for the construction of diaryl phosphonates under metal-free conditions. Nonetheless, the *di*-tert-butyl group on *p*-QM derivatives, which needs extra removal steps, may decrease the synthetic value of the product. Very recently, Kang and co-workers [27] developed the Brønsted acid catalyzed phospha-Michael addition reaction of trialkylphosphites with the *ortho*-quinone methides (*o*-QMs) in situ generated from *o*-hydroxybenzyl alcohols. This strategy needs a catalytic amount of Brønsted acid based on *N*-heterocyclic phosphorodiamidic acids (NHPAs) that should be synthesized from *N*-phosphonyl chloride, which limited its application. Consequently, metal-free and easy operating methods for the synthesis of diarylmethyl phosphine oxides are still highly desirable. Considering the well-documented nucleophilic addition reactions with secondary phosphine oxides (R_2_P(O)H), we hypothesized that the direct phospha-Michael addition of R_2_P(O)H to *o*-QMs would be an attractive way to construct diarylmethyl phosphine oxides with both Lewis base and Brønsted acid functional groups (Scheme 1b).

In general, secondary phosphine oxides exist in two tautomeric forms, i.e., the most stable and dominant phosphane oxide isomer (form **A**), and the phosphinous acid isomer (form **B**), which is the nucleophilic form (Scheme 1b) [28,29]. The equilibrium toward the active phosphinous acid form **B** should be promoted by the presence of a base. In addition, due to the wide application of *o*-QMs in organic synthetic chemistry, several methods have been successfully developed for the generation of *o*-QMs, including oxidation, acid or base catalysis, etc. [30,31,32,33,34,35,36,37,38,39,40,41,42,43]. Zhou and co-workers developed an efficient base-induced approach for the generation of *o*-QMs via the desulfonylation of 2-tosylalkyl phenols under mild conditions [44,45,46,47,48,49,50,51]. Therefore, we supposed that this direct phospha-Michael addition to *o*-QMs in situ generated from 2-tosylalkyl phenols could be realized under appropriate basic conditions. To fulfill this target, the following obstacles should be considered: firstly, the base employed should promote the formation of *o*-QMs and meanwhile facilitate the equilibrium shifts to nucleophilic active form **B**; secondly, the reaction rate of phospha-Michael addition must be faster than the dimerization or other nucleophilic reaction of the in situ generated highly reactive *o*-QMs. In view of the importance of the phosphine oxides, and in order to broaden the application scope of phospha-Michael addition reactions, we report our initial findings toward the construction of bifunctional phosphorus phenols through phospha-Michael addition of diphenylphosphine oxide to the *o*-QMs under basic conditions.

## 2. Results and Discussion

With the abovementioned consideration, we initiated our investigation with the readily available 2-(phenyl(tosyl)methyl)phenol **1a** and diphenylphosphine oxide **2a** in the presence of K_2_CO_3_ (1.2 equiv.) at 60 °C. To our delight, the desired diarylmethyl phosphine oxide product **3a** was isolated in 32% yield (Table 1, Entry 1). Subsequently, the effects of various solvents on the reactivity were investigated (Entries 1–4). Evaluation of solvents revealed that the transformation was sensitive to the reaction medium; no desired product was achieved with dichloromethane (DCM) or tetrahydrofuran (THF) as the solvent, whereas toluene proved to be the most favorable solvent, giving a promising yield of 36%. Further increasing the reaction temperature to 110 °C, the reactivity was effectively improved and high yield of 92% was obtained (Entry 6). The base played a vital role in the reaction, which promotes the desulfonylation to generate *o*-QM, meanwhile facilitating the formation of nucleophilic active phosphinous acid form of secondary phosphine oxide. The screening of several other inorganic bases revealed that K_2_CO_3_ remained superior to Na_2_CO_3_, Cs_2_CO_3_, and NaOH. It is worth noting that the weak base NaHCO_3_ was completely unable to promote this reaction. Therefore, the optimal conditions for this reaction were established by using K_2_CO_3_ (1.2 equiv.) as the base and toluene as the solvent at 110 °C. Then, phosphites including diethyl phosphite and diphenyl phosphite were also investigated as the phosphorus nucleophiles. Disappointingly, no desired products could be isolated under this reaction condition (Entries 11, 12).

With the aforementioned reaction conditions maintained, we next sought to explore the scope of the substrate generality. As summarized in Table 2, the transformations proceeded very well and good to excellent yields were achieved. For aryl substituents such as R^1^, the position of substituents in the aryl ring barely affected the reaction activities (**3a**–**3d**). In addition, the electronic property had a slight influence on the reaction yields. For instance, substrates with different substituents (**1e**, **1f**) reacted with diphenylphosphine oxide in 86% and 80% yields, respectively. The reaction tolerates both electron-deficient and -donating groups on the benzene ring of the phenols, providing the corresponding products in good yields as well (**3g**, **3h**). We also explored the scope of alkyl, aryl-mixed substrates. For example, the 2-(1-tosylethyl)phenol **1i** was demonstrated to be a suitable reaction component and provided the target product **3i** in 84% yield. What is more, the sesamol derived 2-tosylalkyl phenols (**1j**–**1l**) also proved to be suitable substrates, and provided the corresponding products with good yields.

**Scheme 2:**
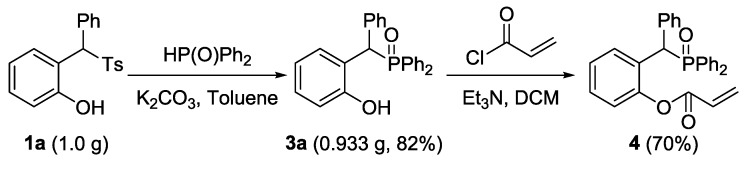
Large-scale experiment and synthetic transformation.

Furthermore, to illustrate the synthetic utility of the diaryl phosphonate products, we first tested a large-scale experiment with **1a** (1.0 g, 5.0 mmol), and it afforded the target adduct **3a** (1.44 g) in 82% yield (Scheme 2). In consideration of the reports that copolymerization of phosphorus phenols with phenolic resin could be used as preeminent flame retardants [52,53], we transformed **3a** to monomers for polymerization reactions. Gratifyingly, the treatment of **3a** with acryloyl chloride under basic conditions afforded the phosphorus acrylate **4** in 70% yield, and thus provided a promising flame-retardant candidate.

**Scheme 3:**
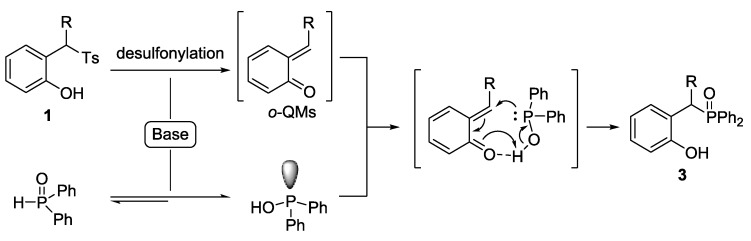
A plausible mechanism.

Based on the above experimental results and previous studies on the formation of *o*-QM under basic conditions reported by Zhou [44,45,46,47,48,49,50,51], a plausible mechanism is depicted in Scheme 3. Firstly, the desulfonylation of 2-(1-tosylalkyl) phenols occurred to generate *o*-QM intermediate in the presence of a suitable base, meanwhile the base promoted the formation of nucleophilic active phosphinous acid (Ph_2_POH). Subsequently, the phosphinous acid proceeded phospha-Michael addition to the active *o*-QM species and delivering the desired product **3**.

## 3. Experimental Section

General Procedures: A reaction mixture of 2-tosylalkyl phenols **1** (0.50 mmol), potassium carbonate (0.6 mmol, 82.9 mg) and diphenylphosphine oxide (0.6 mmol) in toluene (5 mL) was stirred at 110 °C for 4 h. Then water (20 mL) was added to the mixture. The organic layer was separated and the aqueous layer was extracted with dichloromethane (30 mL × 3). The combined organic layer was dried by anhydrous sodium sulfate, concentrated in vacuo. The crude product was purified through column chromatography using dichloromethane and ethyl acetate to give the corresponding product **3**.

*((2-Hydroxyphenyl)(phenyl)methyl)diphenylphosphine oxide* (**3a**). 176.8 mg, 92% yield, unknown compound, pale white solid, m.p.: 238–240 °C, R_f_ = 0.45 (DCM/EA = 50/1); ^1^H-NMR (400 MHz, DMSO-*d*_6_) δ 9.85 (s, 1H), 8.01 (d, *J* = 7.6 Hz, 1H), 7.83–7.77 (m, 2H), 7.75–7.65 (m, 2H), 7.48–7.34 (m, 8H), 7.17–7.13 (m, 2H), 7.12–7.03 (m, 1H), 6.96–6.92 (m, 1H), 6.72–6.68 (m, 2H), 5.62 (d, *J_H-P_* = 9.2 Hz, 1H); ^13^C-NMR (100 MHz, DMSO-*d*_6_) δ 154.9 (d, *J_C-P_* = 7.9 Hz), 137.8 (d, *J_C-P_* = 4.4 Hz), 134.1 (d, *J_C-P_* = 12.2 Hz), 133.1 (d, *J_C-P_* = 12.4 Hz), 131.9 (d, *J_C-P_* = 2.3 Hz), 131.8 (d, *J_C-P_* = 2.3 Hz), 131.2 (d, *J_C-P_* = 8.7 Hz), 130.9 (d, *J_C-P_* = 8.7 Hz), 130.7 (d, *J_C-P_* = 5.7 Hz), 130.3 (d, *J_C-P_* = 6.3 Hz), 128.8 (d, *J_C-P_* = 18.5 Hz), 128.8 (d, *J_C-P_* = 4.1 Hz), 128.5, 128.4, 126.9, 125.0 (d, *J_C-P_* = 3.1 Hz), 119.5, 115.7, 43.2 (d, *J_C-P_* = 68.2 Hz); ^31^P-NMR (162 MHz, DMSO-*d*_6_) δ 31.3; IR (KBr): 3413, 3058, 1576, 1485, 1437, 1275, 1248, 1144, 1119, 811, 750, 691, 560, 530; HRMS (ESI) calcd for C_25_H_22_O_2_P [(M + H)]^+^: 385.1352, found: 385.1352.

## 4. Conclusions

In conclusion, we have developed a concise protocol for the rapid synthesis of bifunctional phosphorus phenols by using diphenylphosphine oxide and 2-(1-tosylalkyl) phenols via the phospha-Michael addition of organophosphorus compounds to in situ generated *o*-QM intermediates under basic condition. This work broadens the scope of phospha-Michael addition reactions that can be employed in the synthesis of diarylmethyl phosphine oxides. Further investigations into the synthetic applications of existing phosphorus phenols are ongoing in our laboratory.

## Figures and Tables

**Table 1:**
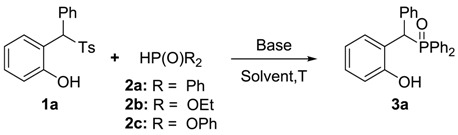
Optimization for the reaction of 2-(phenyl(tosyl)methyl)-phenol **1a** with secondary phosphine oxide *^a^*.

Entry	Solvent	Base	*T*	Yield *^b^*/%
1	EtOAc	K_2_CO_3_	60 °C	32
2	DCE	K_2_CO_3_	60 °C	N.A.
3	THF	K_2_CO_3_	60 °C	N.A.
4	Toluene	K_2_CO_3_	60 °C	36
5	Toluene	K_2_CO_3_	80 °C	73
6	Toluene	K_2_CO_3_	110 °C	92
7	Toluene	Cs_2_CO_3_	110 °C	82
8	Toluene	Na_2_CO_3_	110 °C	68
9	Toluene	NaHCO_3_	110 °C	N.A.
10	Toluene	NaOH	110 °C	86
11 *^c^*	Toluene	K_2_CO_3_	110 °C	N.A.
12 *^d^*	Toluene	K_2_CO_3_	110 °C	N.A.

*^a^* Conditions: **1a** (0.50 mmol), **2a** (0.6 mmol), Base (1.2 equiv.), Solvent (5 mL), 4 h, N.A. = Not Available. *^b^* Isolated yields. *^c^* **2b** (0.6 mmol) was used instead of **2a**. *^d^* **2c** (0.6 mmol) was used instead of **2a**.

**Table 2:** Scope for the reaction of 2-tosylalkyl phenols **1** with diphenylphosphine oxide *^a^*.

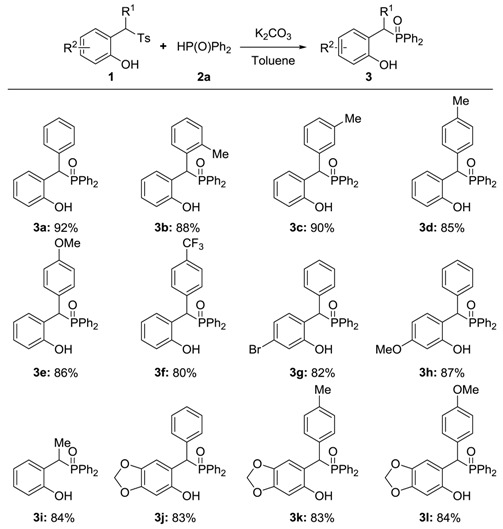

*^a^* Conditions: **1** (0.50 mmol), HP(O)Ph_2_ (0.6 mmol), K_2_CO_3_ (1.2 equiv.), Toluene (5 mL), 110 °C, 4 h, Isolated yields.

## Supplementary Materials

Supplementary File 1
