# Supplementary material for: Straightforward Synthesis of Bifunctional Phosphorus Phenols via Phosphination of In Situ Generated o-Quinone Methides
Source: Molecules. 2018 May 23;23(6):1240. doi: 10.3390/molecules23061240 (PMC6100389; doi:10.3390/molecules23061240)
Supplement: Supplementary File 1 [file molecules-23-01240-s001.pdf]

Supporting Information

Straightforward Synthesis of Bifunctional Phosphorus Phenols *via* Phosphination of *in situ* Generated *o*-Quinone Methides

Zhangpei Chen,* Qinglong Shi, Gongshu Wang, Siwen Chen, and Jianshe Hu*
Center for Molecular Science and Engineering, College of Sciences, Northeastern University, Shenyang 110819 P. R. China.
E-mail address: chenzhangpei@mail.neu.edu.cn (Z. Chen), hujs@mail.neu.edu.cn (J. Hu)

General Information: All compounds were fully characterized by spectroscopic data. The NMR spectra were recorded on a Bruker Avance III 400 M NMR at room temperature (^{1}H: 400 MHz, ^{13}C: 100 MHz, ^{31}P NMR: 162 MHz external standard 85% H_3PO_4, ^{19}F: 376 MHz), chemical shifts (δ) are expressed in parts per million (ppm), coupling constants (*J*) values are given in Hz, and $CDCl_3$ or DMSO-d_6 was used as the solvent. The high resolution mass spectra (HR-MS) data were recorded on Agilent 1290 Infinity LC & 6540 UHD Q-TOF mass spectrometer. IR spectra were recorded on a Thermo Scientific Nicolet 6700 Fourier IR spectrometer (AT-IR) with KBr pellet. Melting points were measured on a digital Electrothermal 9100 apparatus. Commercially available reagents were used without further purification. Solvents were treated prior to use according to the standard methods. The reactions were monitored by thin layer chromatography (TLC) using silica gel GF254. Column chromatography was performed on silica gel (200–300 mesh).

Experimental Section

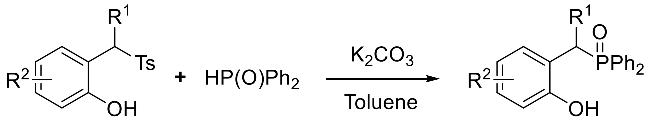

1 **2a** **3**

General Procedure: A reaction mixture of 2-(1-tosylalkyl)phenols **1** (0.50 mmol), potassium carbonate (0.6 mmol, 82.9 mg) and diphenylphosphine oxide **2a** (0.6 mmol) in toluene (5 mL) was stirred at 110 °C for 4 h. Then water (20 mL) was added to the mixture. The organic layer was separated and the aqueous layer was extracted with dichloromethane (30 mL×3). The combined organic layer was dried by anhydrous sodium sulfate, concentrated in *vacuo*. The crude product was purified through column chromatography using dichloromethane and ethyl acetate to give the corresponding product **3**.

A Large-Scale Reaction: A reaction mixture of 2-(phenyl(tosyl)methyl)phenol **1a** (2.96 mmol, 1.002 g), potassium carbonate (3.55 mmol, 0.491 g) and diphenylphosphine oxide (3.55 mmol, 0.718 g) in toluene (25 mL) was stirred at 110 °C for 4 h. Then water (50 mL) was added to the mixture. The organic layer was separated and the aqueous layer was extracted with dichloromethane (50 mL×3). The combined organic layer was dried by anhydrous sodium sulfate, concentrated in *vacuo*. The crude product was purified by column chromatography (dichloromethane : ethyl acetate = 60:1 to 55:1, gradient) to afford **3a** (0.933 g, 82% yield).

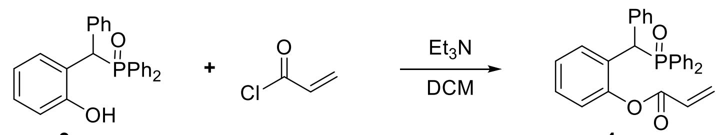

3a **4**

Synthesis of 2-((diphenylphosphoryl)(phenyl)methyl)phenyl acrylate 4: The mixture of **3a** (0.50 mmol, 192.2 mg) and Et_3N (0.75 mmol, 75.9 mg) in dry CH_2Cl_2 (10 mL) was cooled to 0 °C in an ice-water bath and acryloyl chloride (0.75 mmol, 67.9 mg) was added dropwise. The mixture was warmed to room temperature and stirred for 4 h. Following quenching with 2N HCl (10 mL), the mixture was extracted with dichloromethane (30 mL×3). The combined organic phases were sequentially washed with 2N HCl (20 mL), saturated aqueous potassium carbonate solution (20 mL) and brine (20 mL), and then dried over anhydrous sodium sulfate concentrated in *vacuo*. The crude product was purified by column chromatography (dichloromethane : ethyl acetate = 50:1) to afford **4** (154.4 mg, 70% yield).

((2-Hydroxyphenyl)(phenyl)methyl)diphenylphosphine oxide (3a): 176.8 mg, 92% yield, unknown compound, pale white solid, mp: 238-240 °C, R_f = 0.45 (DCM/EA = 50/1); ^{1}H NMR (400 MHz, DMSO-*d*$_6$) δ 9.85 (s, 1H), 8.01 (d, J = 7.6 Hz, 1H), 7.83–7.77 (m, 2H), 7.75–7.65 (m, 2H), 7.48–7.34 (m, 8H), 7.17–7.13 (m, 2H), 7.12–7.03 (m, 1H), 6.96–6.92 (m, 1H), 6.72–6.68 (m, 2H), 5.62 (d, $J_{H\text{-}P}$ = 9.2 Hz, 1H); ^{13}C NMR (100 MHz, DMSO-*d*$_6$) δ 154.9 (d, $J_{C\text{-}P}$ = 7.9 Hz), 137.8 (d, $J_{C\text{-}P}$ = 4.4 Hz), 134.1 (d, $J_{C\text{-}P}$ = 12.2 Hz), 133.1 (d, $J_{C\text{-}P}$ = 12.4 Hz), 131.9 (d, $J_{C\text{-}P}$ = 2.3 Hz), 131.8 (d, $J_{C\text{-}P}$ = 2.3 Hz), 131.2 (d, $J_{C\text{-}P}$ = 8.7 Hz), 130.9 (d, $J_{C\text{-}P}$ = 8.7 Hz), 130.7 (d, $J_{C\text{-}P}$ = 5.7 Hz), 130.3 (d, $J_{C\text{-}P}$ = 6.3 Hz), 128.8 (d, $J_{C\text{-}P}$ = 18.5 Hz), 128.8 (d, $J_{C\text{-}P}$ = 4.1 Hz), 128.5, 128.4, 126.9, 125.0 (d, $J_{C\text{-}P}$ = 3.1 Hz), 119.5, 115.7, 43.2 (d, $J_{C\text{-}P}$ = 68.2 Hz); ^{31}P NMR (162 MHz, DMSO-*d*$_6$) δ 31.3; IR (KBr): 3413, 3058, 1576, 1485, 1437, 1275, 1248, 1144, 1119, 811, 750, 691, 560, 530; HRMS (ESI) calcd for $C_{25}H_{22}O_2P$ $[(M+H)]^+$: 385.1352, found: 385.1352.

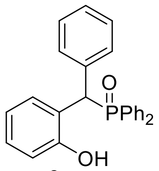

3a

((2-Hydroxyphenyl)(*o*-tolyl)methyl)diphenylphosphine oxide (3b): 175.3 mg, 88% yield, unknown compound, pale white solid, mp: 232-235 °C, R_f = 0.42 (DCM/EA = 50/1); ^{1}H NMR (400 MHz, DMSO-*d*$_6$) δ 9.75 (s, 1H), 8.07 (d, *J* = 7.8 Hz, 1H), 7.88 (d, *J* = 7.7 Hz, 1H), 7.78–7.68 (m, 2H), 7.55–7.38 (m, 6H), 7.36–7.31 (m, 2H), 7.12 (t, *J* = 7.5 Hz, 1H), 7.02 (t, *J* = 7.4 Hz, 1H), 6.98–6.89 (m, 2H), 6.74–6.63 (m, 2H), 5.66 (d, $J_{H\text{-}P}$ = 9.5 Hz, 1H), 2.16 (s, 3H); ^{13}C NMR (100 MHz, DMSO-*d*$_6$) δ 154.8 (d, $J_{C\text{-}P}$ = 7.4 Hz), 137.0 (d, $J_{C\text{-}P}$ = 7.8 Hz), 136.5 (d, $J_{C\text{-}P}$ = 3.6 Hz), 133.9 (d, $J_{C\text{-}P}$ = 95.5 Hz), 133.2 (d, $J_{C\text{-}P}$ = 96.6 Hz), 131.9 (d, $J_{C\text{-}P}$ = 2.8 Hz), 131.8 (d, $J_{C\text{-}P}$ = 2.7 Hz), 131.3 (d, $J_{C\text{-}P}$ = 8.7 Hz), 131.1 (d, $J_{C\text{-}P}$ = 5.4 Hz), 131.0 (d, $J_{C\text{-}P}$ = 8.8 Hz), 130.6 (d, $J_{C\text{-}P}$ = 5.1 Hz), 130.3, 128.8 (d, $J_{C\text{-}P}$ = 2.3 Hz), 128.6 (d, $J_{C\text{-}P}$ = 2.3 Hz), 128.2, 127.0, 126.1, 124.4 (d, $J_{C\text{-}P}$ = 3.9 Hz), 119.4, 115.2, 38.5 (d, $J_{C\text{-}P}$ = 68.4 Hz), 19.9; ^{31}P NMR (162 MHz, DMSO-*d*$_6$) δ 31.4; IR (KBr): 3435, 3039, 2955, 2733, 1596, 1488, 1457, 1438, 1383, 1277, 1157, 1112, 849, 785, 697, 560, 527; HRMS (ESI) calcd for $C_{26}H_{24}O_2P$ $[(M+H)]^+$: 399.1508, found: 399.1508.

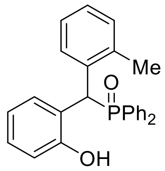

3b

((2-Hydroxyphenyl)(*m*-tolyl)methyl)diphenylphosphine oxide (3c): 179.3 mg, 90% yield, unknown compound, pale white solid, mp: 238-240 °C, R_f = 0.42 (DCM/EA = 50/1); ^{1}H NMR (400 MHz, DMSO-*d*$_6$) δ 9.85 (s, 1H), 7.99 (d, J = 7.6 Hz, 1H), 7.86–7.75 (m, 2H), 7.75–7.63 (m, 2H), 7.45–7.35 (m, 6H), 7.29 (d, J = 7.8 Hz, 1H), 7.24 (s, 1H), 7.04 (t, J = 7.6 Hz, 1H), 6.99–6.84 (m, 2H), 6.72–6.68

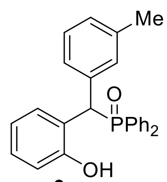
3c

(m 2H), 5.58 (d, J_{H-P} = 9.3 Hz, 1H), 2.13 (s, 3H); ^{13}C NMR (100 MHz, DMSO-d_6) δ 154.9 (d, J_{C-P} = 7.7 Hz), 137.7 (d, J_{C-P} = 4.3 Hz), 137.4, 134.1 (d, J_{C-P} = 12.8 Hz), 133.2 (d, J_{C-P} = 13.1 Hz), 131.8 (d, J_{C-P} = 9.5 Hz), 131.8 (d, J_{C-P} = 4.1 Hz), 131.2 (d, J_{C-P} = 8.8 Hz), 130.9, 130.9, 130.9, 130.8, 130.8, 128.8 (d, J_{C-P} = 5.5 Hz), 128.8 (d, J_{C-P} = 17.0 Hz), 128.4, 127.6, 127.4 (d, J_{C-P} = 6.1 Hz), 124.9 (d, J_{C-P} = 3.1 Hz), 119.5, 115.8, 43.1 (d, J_{C-P} = 68.0 Hz), 21.5; ^{31}P NMR (162 MHz, DMSO-d_6) δ 31.3; IR (KBr): 3426, 3071, 3011, 2951, 2926, 2737, 1603, 1487, 1457, 1437, 1383, 1275, 1241, 1158, 821, 718, 610, 568, 528, 513; HRMS (ESI) calcd for $C_{26}H_{24}O_2P$ $[(M+H)]^+$: 399.1508, found: 399.1506.

((2-Hydroxyphenyl)(*p*-tolyl)methyl)diphenylphosphine oxide (3d): 169.3 mg, 85% yield, unknown compound, pale white solid, mp: 241-245 ºC, R_f = 0.42 (DCM/EA = 50/1); ^{1}H NMR (400 MHz, CDCl$_3$) δ 10.54 (s, 1H), 7.76–7.65 (m, 2H), 7.65–7.54 (m, 2H), 7.51–7.40 (m, 2H), 7.38–7.33 (m, 4H), 7.27–7.24 (m, 2H), 7.07–7.03 (m, 2H), 6.96–6.88 (m, 3H), 6.67 (t, J = 7.4 Hz, 1H), 4.80 (d, J_{H-P} = 12.4 Hz, 1H), 2.22 (s, 3H); ^{13}C NMR (100 MHz, CDCl$_3$) δ 155.8 (d, J_{C-P} = 4.6 Hz), 136.8 (d, J_{C-P} = 1.9 Hz), 132.6 (d, J_{C-P} = 4.0 Hz), 132.3 (d, J_{C-P} = 8.6 Hz), 132.1 (d, J_{C-P} = 1.8 Hz), 132. 1 (d, J_{C-P} = 7.7 Hz), 131.4 (d, J_{C-P} = 9.2 Hz), 131.2 (d, J_{C-P} = 8.8 Hz), 130.7 (d, J_{C-P} = 98.1 Hz), 130.4 (d, J_{C-P} = 99.1 Hz), 129.6 (d, J_{C-P} = 6.2 Hz), 129.2 (d, J_{C-P} = 1.0 Hz), 129.2 (d, J_{C-P} = 1.0 Hz), 128.5 (d, J_{C-P} = 3.5 Hz), 128.5 (d, J_{C-P} = 20.2 Hz), 123.7 (d, J_{C-P} = 4.7 Hz), 120.2, 119.8 (d, J_{C-P} = 1.2 Hz), 53.1 (d, J_{C-P} = 65.3 Hz), 21.0; ^{31}P NMR (162 MHz, CDCl$_3$) δ 38.3; IR (KBr): 3421, 3062, 2925, 2739, 1596, 1511, 1456, 1438, 1386, 1154, 852, 792, 753, 723, 560, 530, 494; HRMS (ESI) calcd for $C_{26}H_{24}O_2P$ $[(M+H)]^+$: 399.1508, found: 399.1510.

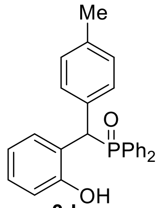
3d

((2-Hydroxyphenyl)(4-methoxyphenyl)methyl)diphenylphosphine oxide (3e): 178.2 mg, 86% yield, unknown compound, pale white solid, mp: 204-205 ºC, R_f = 0.32 (DCM/EA = 50/1); ^{1}H NMR (400 MHz, CDCl$_3$) δ 10.54 (s, 1H), 7.75–7.67 (m, 2H), 7.62–7.54 (m, 2H), 7.49–7.41 (m, 2H), 7.39–7.33 (m, 4H), 7.30–7.26 (m, 2H), 7.08–7.03 (m, 2H), 6.92–6.88 (m, 1H), 6.72–6.64 (m, 3H), 4.78 (d, J_{H-P} = 12.4 Hz, 1H), 3.70 (s, 3H); ^{13}C NMR (100 MHz, CDCl$_3$) δ 158.6 (d, J_{C-P} = 1.7 Hz), 155.7 (d, J_{C-P} = 4.7 Hz), 132.2 (d, J_{C-P} = 8.8 Hz), 132.1 (d, J_{C-P} = 7.0 Hz), 132.1 (d, J_{C-P} = 1.2 Hz), 131.4 (d, J_{C-P} = 9.2 Hz), 131.2 (d, J_{C-P} = 8.9 Hz), 130.8 (d, J_{C-P} = 6.0 Hz), 130.6 (d, J_{C-P} = 97.7 Hz), 130.3 (d, J_{C-P} = 99.1 Hz), 129.2, 128.5 (d, J_{C-P} = 3.6 Hz), 128.5 (d, J_{C-P} = 20.1 Hz), 127.6 (d, J_{C-P} = 4.0 Hz), 123.8 (d, J_{C-P} = 4.4 Hz), 120.2, 119.9 (d, J_{C-P} = 1.0 Hz), 113.9, 55.2, 52.8 (d, J_{C-P} = 65.6 Hz); ^{31}P NMR (162 MHz, CDCl$_3$) δ 38.4; IR (KBr): 3425, 3060, 2958, 1608, 1510, 1455, 1438, 1384, 1249, 1154, 1118, 1031, 831, 784, 754, 561, 529; HRMS (ESI) calcd for $C_{26}H_{24}O_3P$ $[(M+H)]^+$: 415.1458, found: 415.1457.

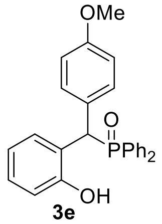
3e

((2-Hydroxyphenyl)(4-(trifluoromethyl)phenyl)methyl)diphenylphosphine oxide (3f): 180.8 mg, 80% yield, unknown compound, pale white solid, mp: 187-189 ºC, R_f = 0.46 (DCM/EA = 50/1); ^{1}H NMR (400 MHz, CDCl$_3$) δ 10.02 (s, 1H), 7.76–7.70 (m, 2H), 7.62–7.50 (m, 4H), 7.49–7.28 (m, 9H), 7.03 (t, J = 7.8 Hz, 1H), 6.88 (d, J = 8.1 Hz, 1H), 6.70 (t, J = 7.5 Hz, 1H), 5.12 (d, J_{H-P} = 10.9 Hz, 1H); ^{13}C NMR (100 MHz, CDCl$_3$) δ 155.2 (d, J_{C-P} = 5.8 Hz), 140.2 (d, J_{C-P} = 2.9 Hz), 132.3 (d, J_{C-P} = 2.8 Hz), 132.2 (d, J_{C-P} = 2.9 Hz), 131.8 (d, J_{C-P} = 7.6 Hz), 131.2 (d, J_{C-P} = 19.5 Hz), 131.2, 130.9 (d, J_{C-P} = 5.4 Hz), 130.7, 130.2 (d, J_{C-P} = 6.1 Hz), 129.9 (d, J_{C-P} = 4.6 Hz), 129.3, 129.0 (d, J_{C-P} = 13.1

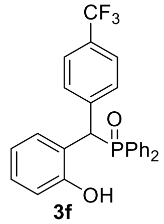
3f

Hz), 128.7 (d, $J_{C\text{-}P}$ = 1.7 Hz), 128.6 (d, $J_{C\text{-}P}$ = 1.8 Hz), 125.4, 125.2 (q, $J_{C\text{-}F}$ = 4.0 Hz), 123.0 (d, $J_{C\text{-}P}$ = 4.4 Hz), 122.7, 120.4, 118.6, 50.3 (d, $J_{C\text{-}P}$ = 66.1 Hz); ^{31}P NMR (162 MHz, $CDCl_3$) δ 37.1; ^{19}F NMR (376 MHz, $CDCl_3$) δ -62.6; IR (KBr): 3428, 3061, 1619, 1455, 1439, 1325, 1167, 1121, 1068, 761, 726, 699, 557; HRMS (ESI) calcd for $C_{26}H_{21}F_3O_2P$ $[(M+H)]^+$: 453.1226, found: 453.1223.

((4-Bromo-2-hydroxyphenyl)(phenyl)methyl)diphenylphosphine oxide (3g): 189.9 mg, 82% yield, unknown compound, pale white solid, mp: 270-271 °C, R_f = 0.46 (DCM/EA = 50/1); ^{1}H NMR (400 MHz, DMSO-*d₆*) δ 10.35 (s, 1H), 7.99–7.96 (m, 1H), 7.83–7.78 (m, 2H), 7.74–7.68 (m, 2H), 7.52–7.34 (m, 8H), 7.18–7.07 (m, 2H), 7.12–7.05 (m, 1H), 6.94–6.91 (m, 1H), 6.89–6.87 (m, 1H), 5.56 (d, $J_{H\text{-}P}$ = 9.1 Hz, 1H); ^{13}C NMR (100 MHz, DMSO-d_6) δ 156.2 (d, $J_{C\text{-}P}$ = 7.7 Hz), 137.2 (d, $J_{C\text{-}P}$ = 4.4 Hz), 133.8 (d, $J_{C\text{-}P}$ = 23.3 Hz), 132.8 (d, $J_{C\text{-}P}$ = 24.3 Hz), 132.3 (d, $J_{C\text{-}P}$ = 5.5 Hz), 132.1 (d, $J_{C\text{-}P}$ = 2.9 Hz), 131.9 (d, $J_{C\text{-}P}$ = 2.6 Hz), 131.2 (d, $J_{C\text{-}P}$ = 8.7 Hz), 130. 9 (d, $J_{C\text{-}P}$ = 8.8 Hz), 130.2 (d, $J_{C\text{-}P}$ = 6.2 Hz), 128.9 (d, $J_{C\text{-}P}$ = 3.2 Hz), 128.9 (d, $J_{C\text{-}P}$ = 26.0 Hz), 128.6, 127.1, 124.7 (d, $J_{C\text{-}P}$ = 3.2 Hz), 122.3, 120.7, 118.3, 42.7 (d, $J_{C\text{-}P}$ = 67.5 Hz); ^{31}P NMR (162 MHz, DMSO-d_6) δ 31.0; IR (KBr): 3422, 3025, 2901, 2718, 1589, 1491, 1419, 1260, 1145, 1116, 1095, 1072, 887, 859, 837, 726, 537, 501; HRMS (ESI) calcd for $C_{25}H_{21}BrO_2P$ $[(M+H)]^+$: 463.0457, found: 463.0453.

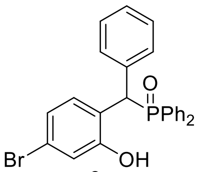

3g

((2-Hydroxy-4-methoxyphenyl)(*p*-tolyl)methyl)diphenylphosphine oxide (3h): 186.4 mg, 87% yield, unknown compound, pale white solid, mp: 240-241 °C, R_f = 0.34 (DCM/EA = 50/1); ^{1}H NMR (400 MHz, $CDCl_3$) δ 10.80 (s, 1H), 7.76–7.67 (m, 2H), 7.60–7.55 (m, 2H), 7.48–7.41 (m, 2H), 7.39–7.32 (m, 4H), 7.23–7.20 (m, 2H), 6.95–6.90 (m, 3H), 6.47 (d, *J* = 2.6 Hz, 1H), 6.27–6.24 (m, 1H), 4.73 (d, $J_{H\text{-}P}$ = 12.7 Hz, 1H), 3.67 (s, 3H), 2.21 (s, 3H); ^{13}C NMR (100 MHz, $CDCl_3$) δ 160.4 (d, $J_{C\text{-}P}$ = 1.4 Hz), 157.0 (d, $J_{C\text{-}P}$ = 4.6 Hz), 136.7 (d, $J_{C\text{-}P}$ = 2.0 Hz), 132.9 (d, $J_{C\text{-}P}$ = 8.9 Hz), 132.9 (d, $J_{C\text{-}P}$ = 3.6 Hz), 132.1 (d, $J_{C\text{-}P}$ = 5.4 Hz), 132.1, 131.4 (d, $J_{C\text{-}P}$ = 9.2 Hz), 131.2 (d, $J_{C\text{-}P}$ = 8.8 Hz), 130.8 (d, $J_{C\text{-}P}$ = 97.8 Hz), 130.4 (d, $J_{C\text{-}P}$ = 98.5 Hz), 129.5 (d, $J_{C\text{-}P}$ = 6.1 Hz), 129.2 (d, $J_{C\text{-}P}$ = 1.3 Hz), 128.5 (d, $J_{C\text{-}P}$ = 2.2 Hz), 128.5 (d, $J_{C\text{-}P}$ = 25.8 Hz), 116.0 (d, $J_{C\text{-}P}$ = 4.6 Hz), 107.0, 104.4 (d, $J_{C\text{-}P}$ = 1.5 Hz), 55.1, 52.5 (d, $J_{C\text{-}P}$ = 65.6 Hz), 21.0; ^{31}P NMR (162 MHz, $CDCl_3$) δ 38.7; IR (KBr): 3412, 3058, 3007, 2940, 2898, 1736, 1615, 1524, 1437, 1153, 1036, 854, 802, 720, 696, 536; HRMS (ESI) calcd for $C_{27}H_{26}O_3P$ $[(M+H)]^+$: 429.1614, found: 429.1617.

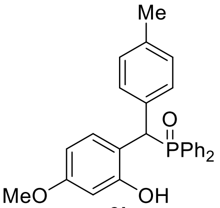

3h

(1-(2-Hydroxyphenyl)ethyl)diphenylphosphine oxide (3i): 135.4 mg, 84% yield, unknown compound, pale white solid, mp: 198-200 °C, R_f = 0.45 (DCM/EA = 50/1); ^{1}H NMR (400 MHz, $CDCl_3$) δ 10.20 (s, 1H), 7.94–7.80 (m, 2H), 7.70–7.48 (m, 5H), 7.47–7.28 (m, 3H), 7.13–7.04 (m, 1H), 6.96–6.93 (m, 1H), 6.84–6.81 (m, 1H), 6.67 (t, *J* = 7.4 Hz, 1H), 3.64 (dt, *J* = 9.4, 7.4 Hz, 1H), 1.59 (dd, *J* = 15.9, 7.5 Hz, 3H); ^{13}C NMR (100 MHz, $CDCl_3$) δ 156.3 (d, $J_{C\text{-}P}$ = 4.1 Hz), 132.3 (d, $J_{C\text{-}P}$ = 2.9 Hz), 132.0 (d, $J_{C\text{-}P}$ = 2.8 Hz), 131.3 (d, $J_{C\text{-}P}$ = 7.3 Hz), 131.2 (d, $J_{C\text{-}P}$ = 8.9 Hz), 130.9 (d, $J_{C\text{-}P}$ = 9.1 Hz),130.2 (d, $J_{C\text{-}P}$ = 6.5 Hz), 129.2, 128.9 (d, $J_{C\text{-}P}$ = 2.1 Hz), 128.9 (d, $J_{C\text{-}P}$ = 11.5 Hz), 128.5 (d, $J_{C\text{-}P}$ = 11.7 Hz), 124.5 (d, $J_{C\text{-}P}$ = 5.8 Hz), 120.2, 119.9 (d, $J_{C\text{-}P}$ = 2.1 Hz), 40.7 (d, $J_{C\text{-}P}$ = 67.2 Hz), 13.0 (d, $J_{C\text{-}P}$ = 2.2 Hz); ^{31}P NMR (162 MHz, $CDCl_3$) δ 41.6; IR (KBr): 3426, 3060, 2961, 1592, 1451, 1437, 1390, 1158, 1119, 1091, 1020, 778, 751, 721, 696, 605, 556; HRMS (ESI) calcd for $C_{20}H_{20}O_2P$ $[(M+H)]^+$: 323.1195, found: 323.1196.

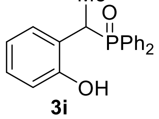

3i

((6-Hydroxybenzo[*d*][1,3]dioxol-5-yl)(phenyl)methyl)diphenylphosphine oxide (3j): 177.8 mg, 83% yield, unknown compound, pale white solid, mp: 253-255 °C, R_f = 0.42 (DCM/EA = 40/1);^{1}H NMR (400 MHz, DMSO-d_6) δ 9.64 (s, 1H), 7.84–7.78 (m, 2H), 7.73–7.64 (m, 2H), 7.58–7.56 (m, 1H), 7.52–7.31 (m, 8H), 7.17–7.13 (m, 2H), 7.12–7.04 (m, 1H), 6.35–6.34 (m, 1H), 5.86–5.80 (m, 2H), 5.54 (d, $J_{H\text{-}P}$ = 9.6 Hz, 1H); ^{13}C NMR (100 MHz, DMSO-d_6) δ 149.3 (d, $J_{C\text{-}P}$ = 8.2 Hz), 146.2, 139.5, 137.5 (d, $J_{C\text{-}P}$ = 4.1 Hz), 133.6 (d, $J_{C\text{-}P}$ = 15.8 Hz), 132.6 (d, $J_{C\text{-}P}$ = 15.7 Hz), 131.5 (d, $J_{C\text{-}P}$ = 2.6 Hz), 131.3 (d, $J_{C\text{-}P}$ = 2.4 Hz), 131.4 (d, $J_{C\text{-}P}$ = 14.7 Hz), 130.5 (d, $J_{C\text{-}P}$ = 32.2 Hz), 130.5 (d, $J_{C\text{-}P}$ = 14.8 Hz), 129.6 (d, $J_{C\text{-}P}$ = 6.2 Hz), 128.4 (d, $J_{C\text{-}P}$ = 4.5 Hz), 128.4 (d, $J_{C\text{-}P}$ = 27.0 Hz), 128.0, 126.4, 115.7 (d, $J_{C\text{-}P}$ = 3.3 Hz), 109.2 (d, $J_{C\text{-}P}$ = 5.5 Hz), 100.6, 97.5, 42.3 (d, $J_{C\text{-}P}$ = 68.2 Hz); ^{31}P NMR (162 MHz, DMSO-d_6) δ 31.7; IR (KBr): 3431, 3057, 2926, 1626, 1504, 1438, 1289, 1158, 1118, 1040, 942, 872, 742, 723, 540; HRMS (ESI) calcd for $C_{26}H_{22}O_4P$ $[(M+H)]^+$: 429.1256, found: 429.1251.

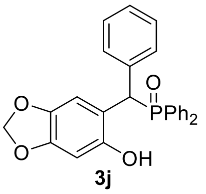

3j

((6-Hydroxybenzo[*d*][1,3]dioxol-5-yl)(*p*-tolyl)methyl)diphenylphosphine oxide (3k): 183.6 mg, 83% yield, unknown compound, pale white solid, mp: 236-237 °C, R_f = 0.42 (DCM/EA = 40/1); ^{1}H NMR (400 MHz, $CDCl_3$) δ 10.40 (s, 1H), 7.74–7.65 (m, 2H), 7.62–7.52 (m, 2H), 7.51–7.44 (m, 2H), 7.41–7.33 (m, 4H), 7.24–7.16 (m, 2H), 6.97–6.93 (m, 2H), 6.49–6.45 (m, 2H), 5.86–5.74 (m, 2H), 4.66 (d, $J_{H\text{-}P}$ = 13.5 Hz, 1H), 2.23 (s, 3H); ^{13}C NMR (100 MHz, $CDCl_3$) δ 151.2 (d, $J_{C\text{-}P}$ = 4.9 Hz), 147.9 (d, $J_{C\text{-}P}$ = 1.2 Hz), 141.0, 136.9 (d, $J_{C\text{-}P}$ = 1.8 Hz), 132.7 (d, $J_{C\text{-}P}$ = 3.3 Hz), 132.1 (d, $J_{C\text{-}P}$ = 4.9 Hz), 132.1, 131.4 (d, $J_{C\text{-}P}$ = 9.2 Hz), 131.2 (d, $J_{C\text{-}P}$ = 8.8 Hz), 130.7 (d, $J_{C\text{-}P}$ = 98.2 Hz), 130.4 (d, $J_{C\text{-}P}$ = 98.1 Hz), 129.6 (d, $J_{C\text{-}P}$ = 6.4 Hz), 129.2, 128.7, 128.5, 128.4, 115.2 (d, $J_{C\text{-}P}$ = 4.8 Hz), 110.5 (d, $J_{C\text{-}P}$ = 8.8 Hz), 101.5 (d, $J_{C\text{-}P}$ = 1.5 Hz), 101.1, 52.4 (d, $J_{C\text{-}P}$ = 65.8 Hz), 21.0; ^{31}P NMR (162 MHz, $CDCl_3$) δ 38.6; IR (KBr): 3422, 3055, 2923, 1623, 1504, 1438, 1158, 1039, 938, 975, 721, 696, 538; HRMS (ESI) calcd for $C_{27}H_{24}O_4P$ $[(M+H)]^+$: 443.1407, found: 443.1406.

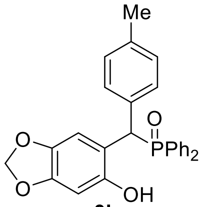

3k

((6-Hydroxybenzo[*d*][1,3]dioxol-5-yl)(4-methoxyphenyl)methyl)diphenylphosphine oxide (3l): 192.5 mg, 84% yield, unknown compound, pale white solid, mp: 231-233 °C, R_f = 0.42 (DCM/EA = 40/1);^{1}H NMR (400 MHz, DMSO-d_6) δ 9.58 (s, 1H), 7.80–7.75 (m, 2H), 7.70–7.65 (m, 2H), 7.53–7.32 (m, 9H), 6.75–6.68 (m, 2H), 6.31 (s, 1H), 5.82 (m, 2H), 5.46 (d, $J_{H\text{-}P}$ = 9.8 Hz, 1H), 3.63 (s, 3H); ^{13}C NMR (100 MHz, DMSO-d_6) δ 158.2, 149.7 (d, $J_{C\text{-}P}$ = 7.8 Hz), 146.6, 140.0, 134.2 (d, $J_{C\text{-}P}$ = 15.5 Hz), 133.3 (d, $J_{C\text{-}P}$ = 16.4 Hz), 131.8 (d, $J_{C\text{-}P}$ = 12.2 Hz), 131.1 (d, $J_{C\text{-}P}$ = 4.5 Hz), 131.1, 130.9 (d, $J_{C\text{-}P}$ = 8.6 Hz), 129.9 (d, $J_{C\text{-}P}$ = 4.0 Hz), 128.9 (d, $J_{C\text{-}P}$ = 1.3 Hz), 128.9 (d, $J_{C\text{-}P}$ = 20.2 Hz), 116.7 (d, $J_{C\text{-}P}$ = 2.9 Hz), 114.0, 109.6 (d, $J_{C\text{-}P}$ = 3.8 Hz), 101.1, 98.0, 55.4, 41.8 (d, $J_{C\text{-}P}$ = 69.2 Hz); ^{31}P NMR (162 MHz, DMSO-d_6) δ 31.9; IR (KBr): 3425, 3061, 2960, 2903, 1607, 1509, 1439, 1245, 1174, 1154, 1113, 1048, 942, 877, 833, 727, 700, 593, 542, 523; HRMS (ESI) calcd for $C_{27}H_{24}O_5P$ $[(M+H)]^+$: 459.1356, found: 459.1354.

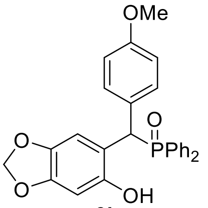

3l

2-((Diphenylphosphoryl)(phenyl)methyl)phenyl acrylate (4): 154.3 mg, 70% yield, unknown compound, pale white solid, mp: 78-79 °C, R_f = 0.38 (DCM/EA = 40/1); ^{1}H NMR (400 MHz, $CDCl_3$) δ 8.35–8.25 (m, 1H), 7.74–7.62 (m, 2H), 7.53–7.44 (m, 2H), 7.44–7.31 (m, 4H), 7.30–7.24 (m, 4H), 7.21–7.15 (m, 2H), 7.14–7.10 (m, 3H), 7.01–6.92 (m, 1H), 6.53 (dd, *J* = 17.3, 1.3 Hz, 1H), 6.29 (dd, *J*

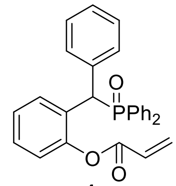

4

= 17.3, 10.4 Hz, 1H), 6.03 (dd, J = 10.4, 1.3 Hz, 1H), 4.93 (d, J_{H-P} = 9.2 Hz, 1H); ^{13}C NMR (100 MHz, $CDCl_3$) δ 163.9, 148.2 (d, J_{C-P} = 8.8 Hz), 135.6 (d, J_{C-P} = 5.2 Hz), 133.1, 132.5 (d, J_{C-P} = 99.7 Hz), 132.1 (d, J_{C-P} = 97.2 Hz), 131.6 (d, J_{C-P} = 3.0 Hz), 131.5 (d, J_{C-P} = 2.6 Hz), 131.3, 131.3 (d, J_{C-P} = 2.2 Hz), 131.2 (d, J_{C-P} = 2.3 Hz), 130.2 (d, J_{C-P} = 6.1 Hz), 129.9 (d, J_{C-P} = 2.9 Hz), 128.4 (d, J_{C-P} = 11.6 Hz), 128.3 (d, J_{C-P} = 1.3 Hz), 128.2, 128.1, 127.9 (d, J_{C-P} = 62.5 Hz), 127.0 (d, J_{C-P} = 2.1 Hz), 126.4, 45.70 (d, J_{C-P} = 67.4 Hz); ^{31}P NMR (162 MHz, $CDCl_3$) δ 31.6; IR (KBr): 3052, 3019, 2925, 1739, 1486, 1436, 1402, 1139, 982, 800, 724, 693, 553, 515; HRMS (ESI) calcd for $C_{28}H_{24}O_3P$ $[(M+H)]^+$: 439.1458, found: 439.1455.

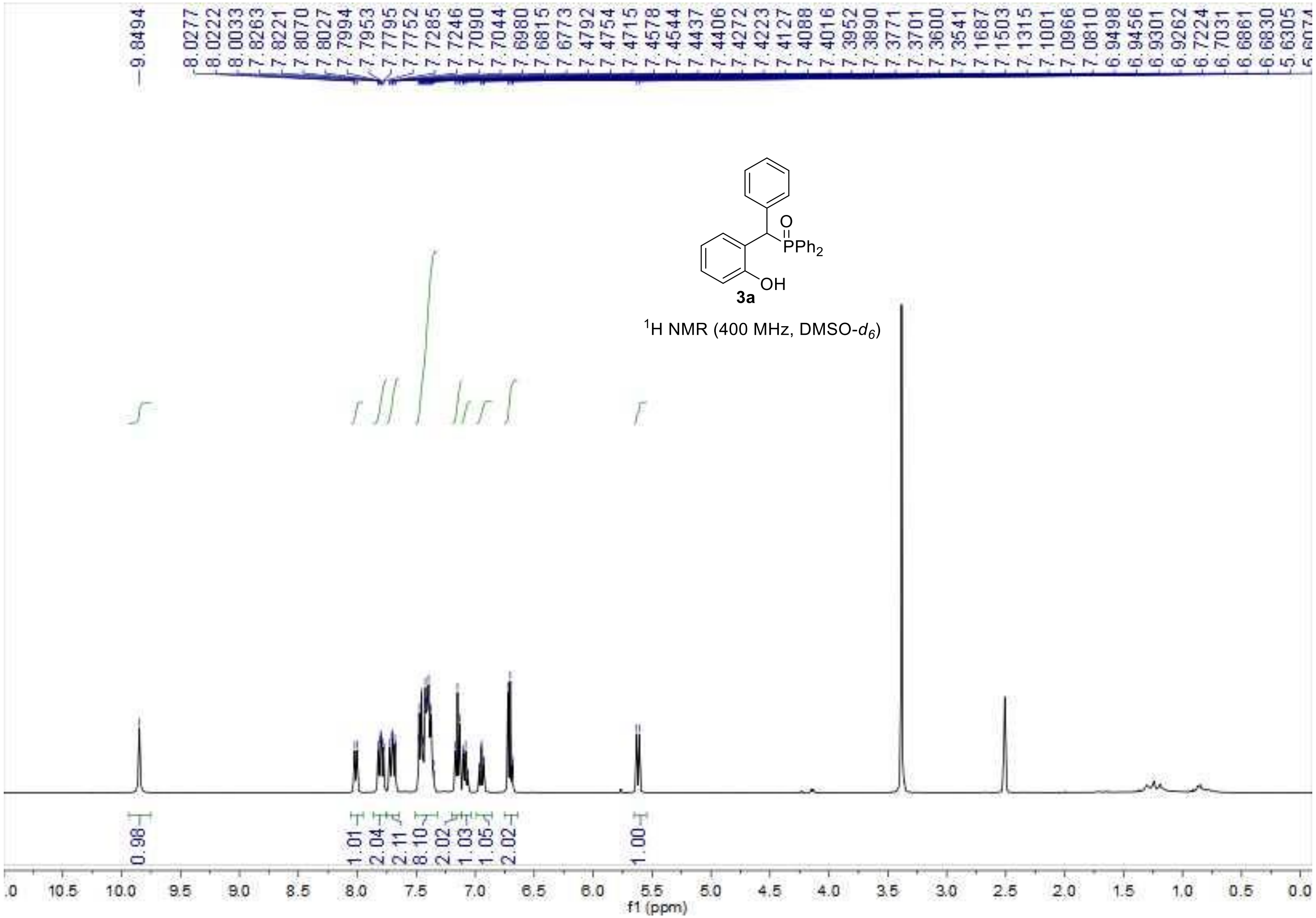

9.8494
8.0277 8.0222 8.0033 7.8263 7.8221 7.8070 7.8027 7.7994 7.7953 7.7795 7.7752 7.7285 7.7246 7.7090 7.7044 7.6980 7.6815 7.6773 7.4792 7.4754 7.4715 7.4578 7.4544 7.4437 7.4406 7.4272 7.4223 7.4127 7.4088 7.4016 7.3952 7.3890 7.3771 7.3701 7.3600 7.3541 7.1687 7.1503 7.1315 7.1001 7.0966 7.0810 6.9498 6.9456 6.9301 6.9262 6.7224 6.7031 6.6861 6.6830 5.6305
O
PPh2
OH
3a
1H NMR (400 MHz, DMSO-d6)
0.98
1.01 2.04 2.11 8.10 2.02 1.03 1.05 2.02
1.00
f1 (ppm)

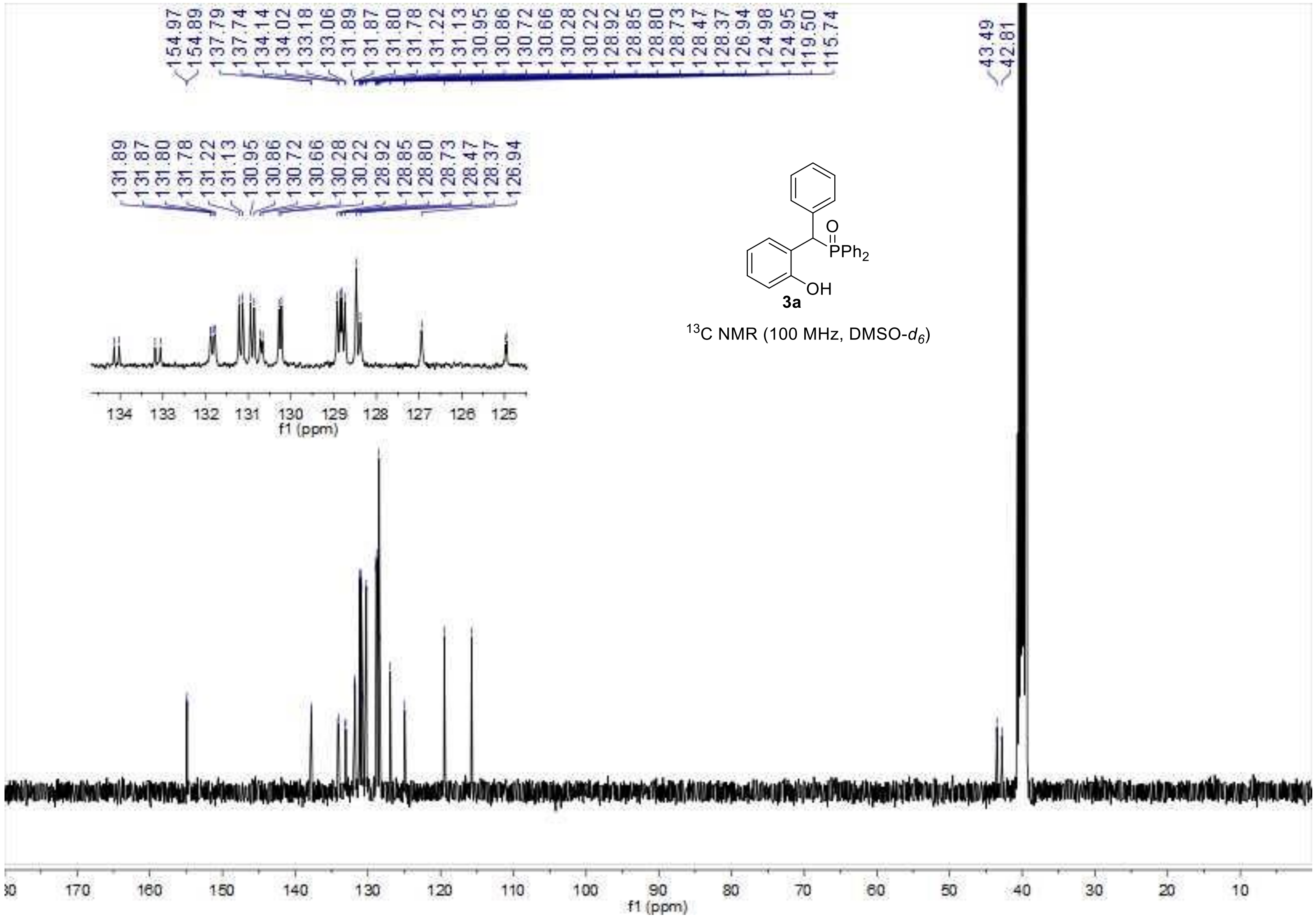

154.97
154.89
137.79
137.74
134.14
134.02
133.18
133.06
131.89
131.87
131.80
131.78
131.22
131.13
130.95
130.86
130.72
130.66
130.28
130.22
128.92
128.85
128.80
128.73
128.47
128.37
126.94
124.98
124.95
119.50
115.74
43.49
42.81
O
PPh2
OH
3a
13C NMR (100 MHz, DMSO-d6)
f1 (ppm)

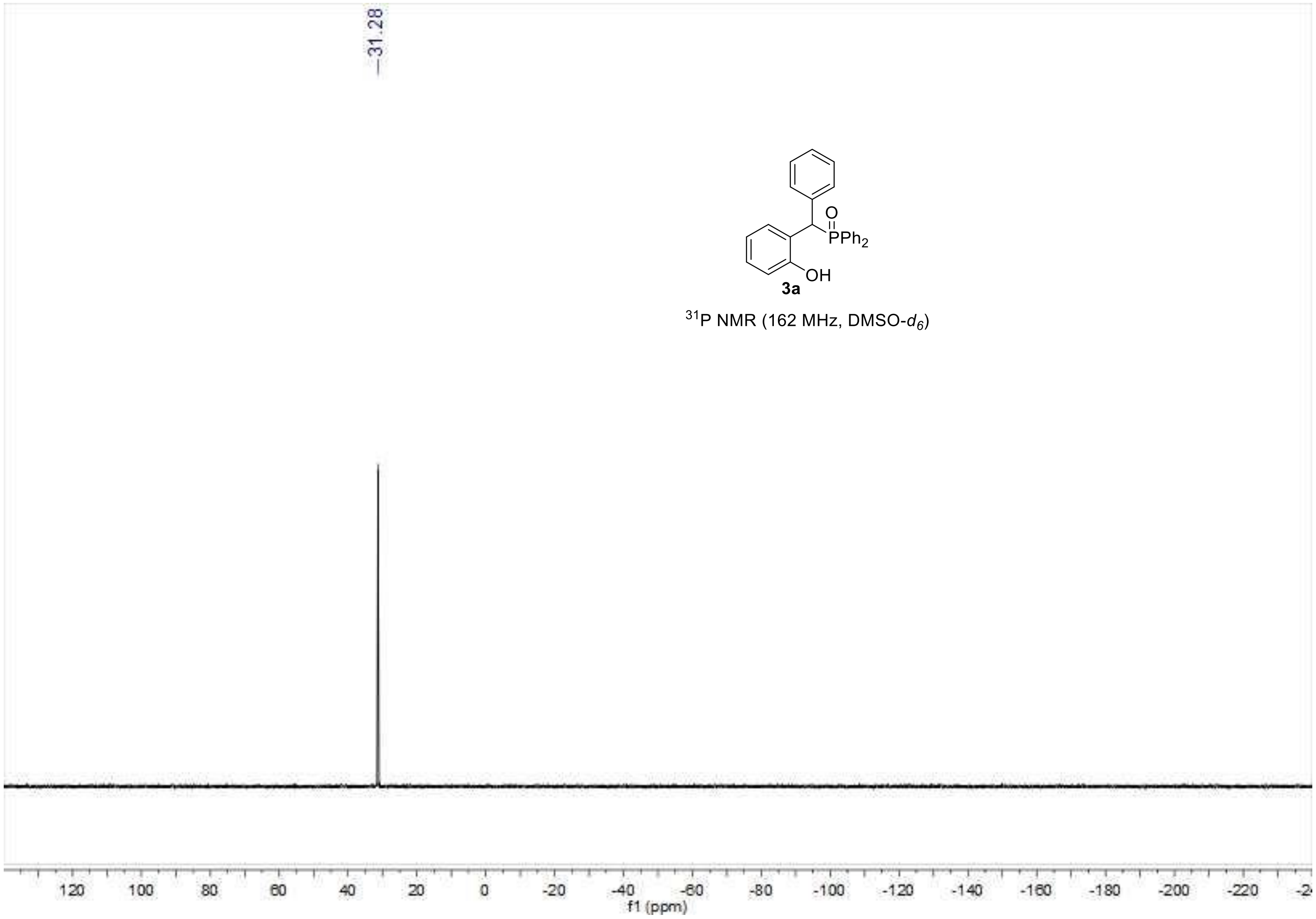

^{31}P NMR (162 MHz, DMSO-d_6)

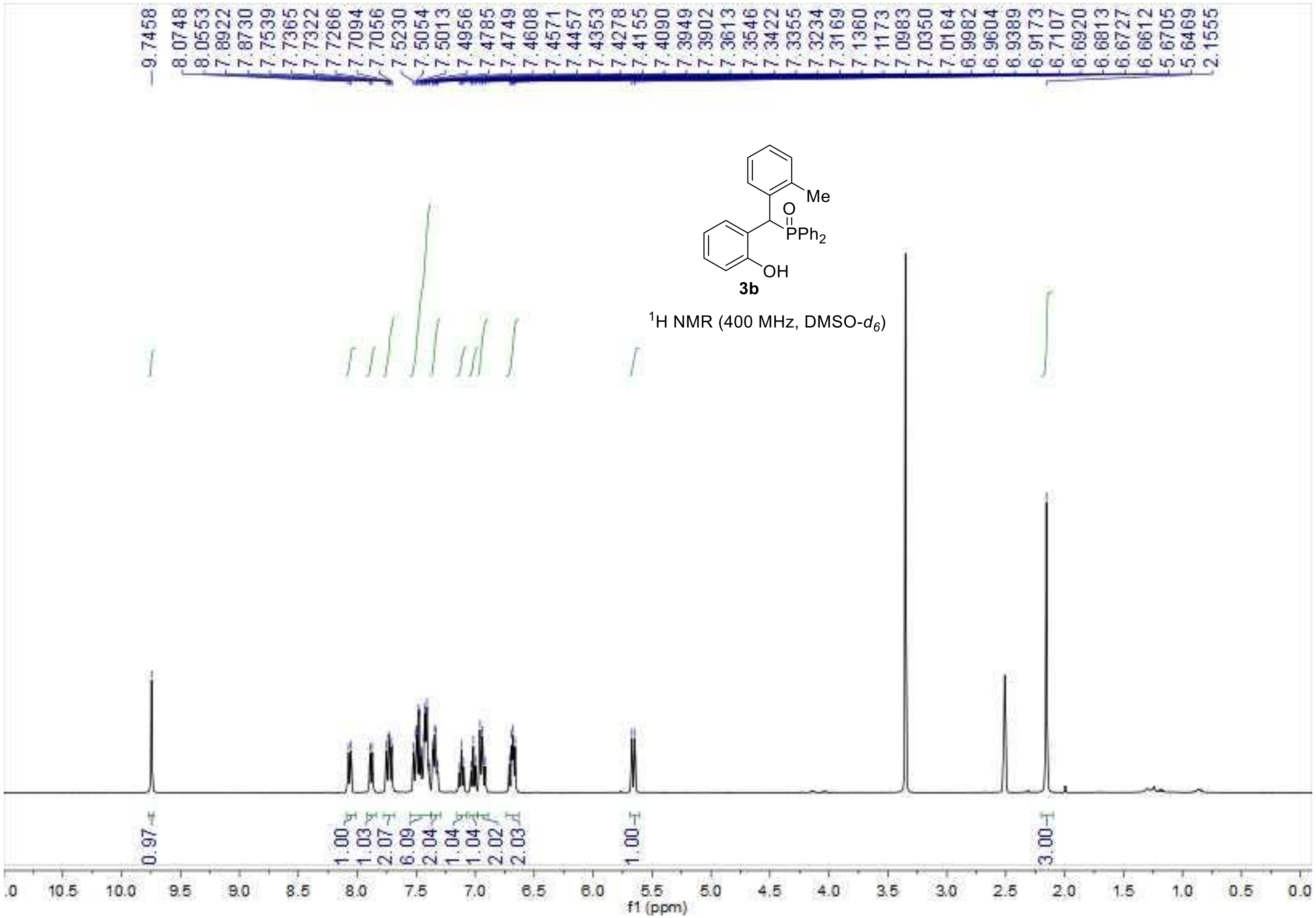

3b

^{1}H NMR (400 MHz, DMSO-d_6)

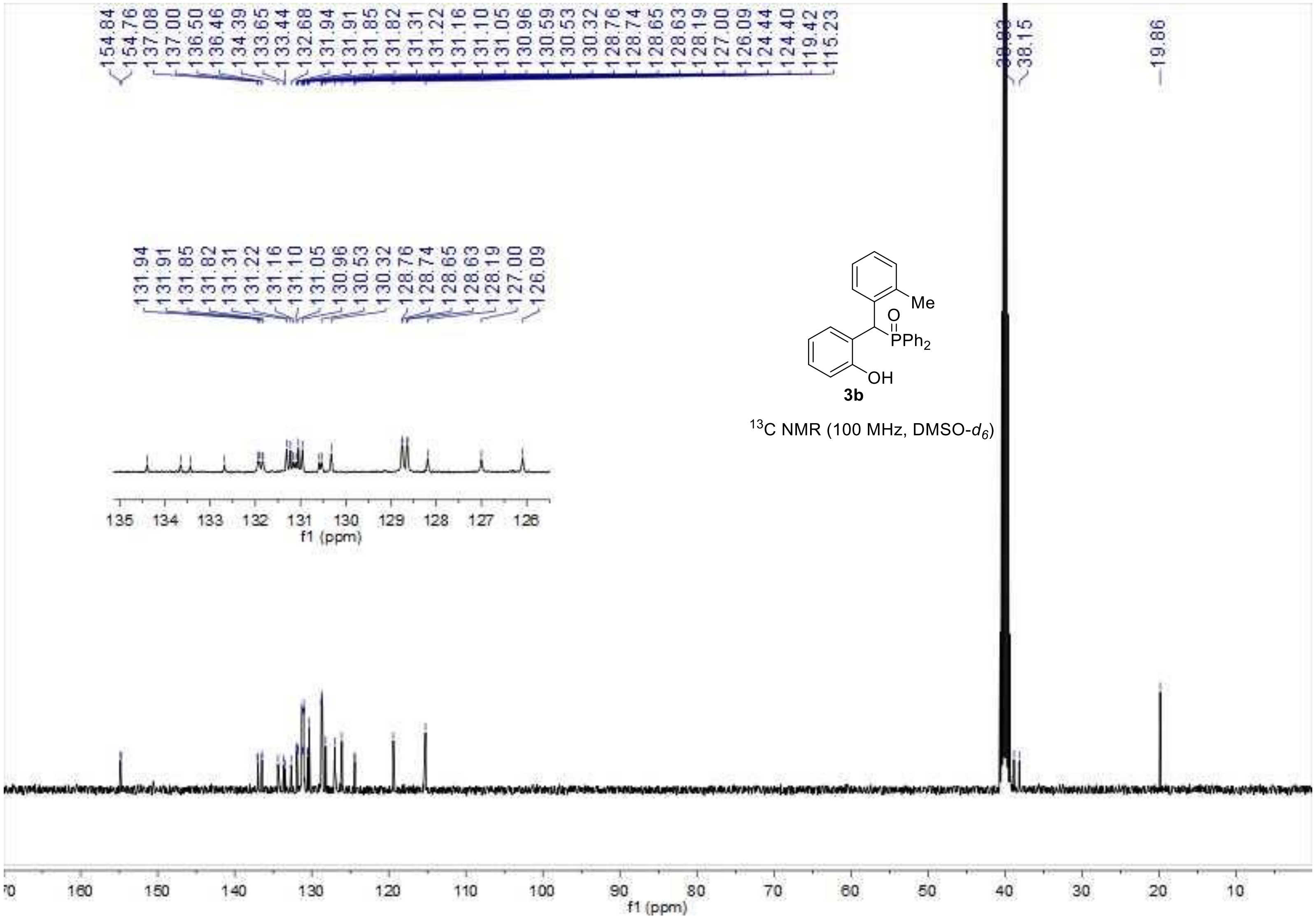
154.84
154.76
137.08
137.00
136.50
136.46
134.39
133.65
133.44
132.68
131.94
131.91
131.85
131.82
131.31
131.22
131.16
131.10
131.05
130.96
130.59
130.53
130.32
128.76
128.74
128.65
128.63
128.19
127.00
126.09
124.44
124.40
119.42
115.23
38.15
19.86
Me
O
PPh2
OH
3b
13C NMR (100 MHz, DMSO-d6)
f1 (ppm)
f1 (ppm)

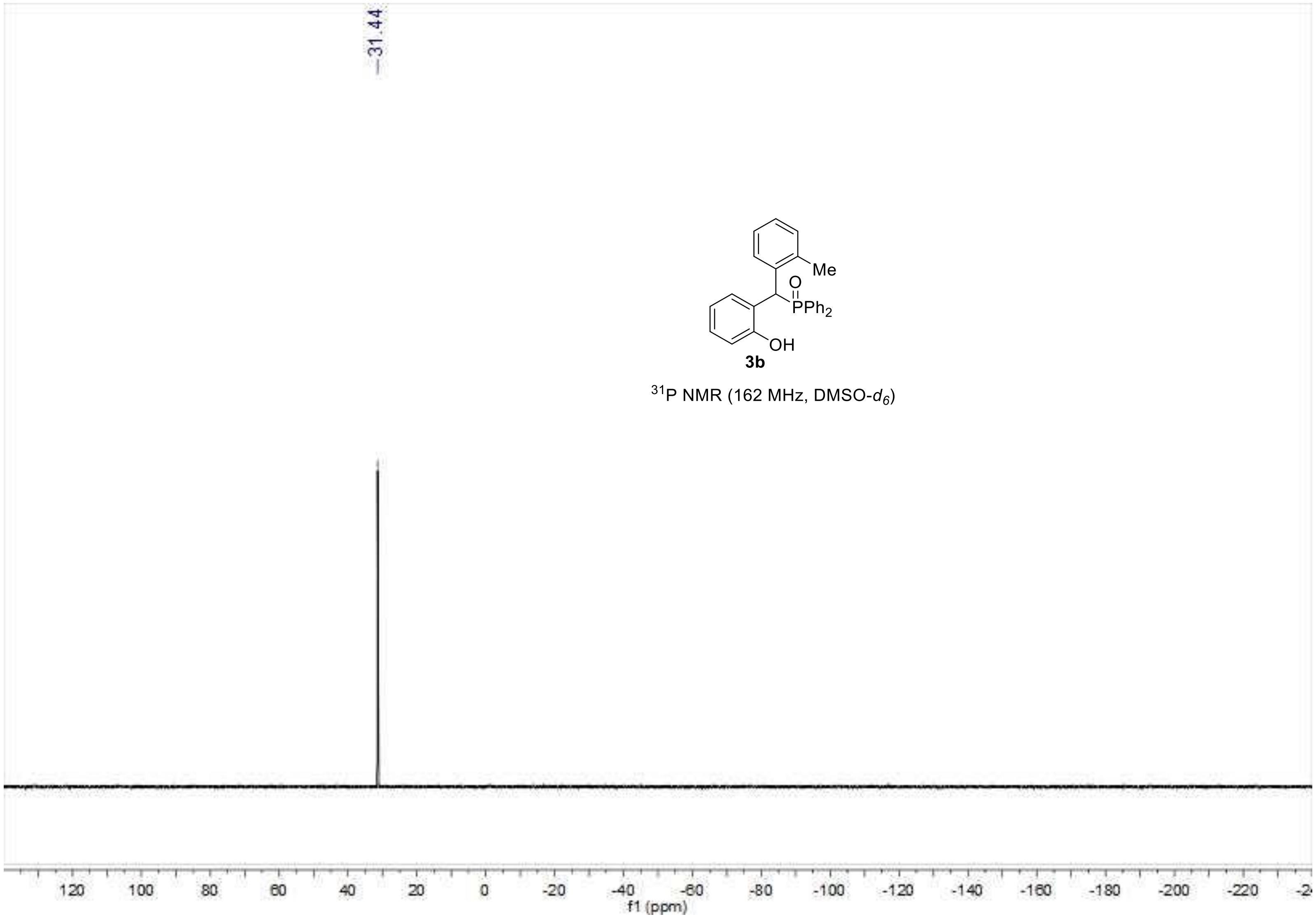

31.44
Me
O
PPh2
OH
3b
^{31}P NMR (162 MHz, DMSO-d_6)
120
100
80
60
40
20
0
-20
-40
-60
-80
-100
-120
-140
-160
-180
-200
-220
f1 (ppm)

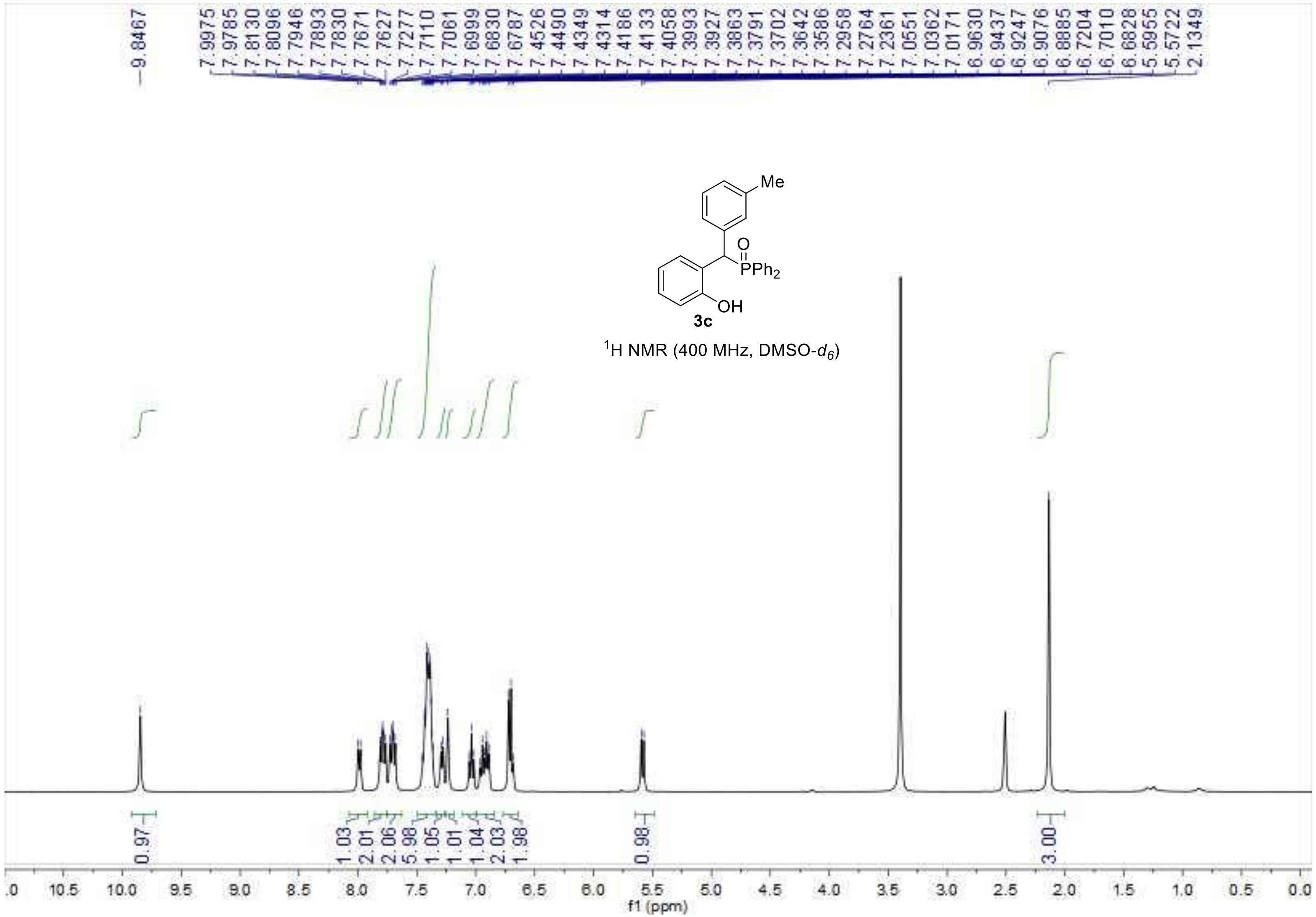

9.8467
7.9975 7.9785 7.8130 7.8096 7.7946 7.7893 7.7830 7.7671 7.7627 7.7277 7.7110 7.7061 7.6999 7.6830 7.6787 7.4526 7.4490 7.4349 7.4314 7.4186 7.4133 7.4058 7.3993 7.3927 7.3863 7.3791 7.3702 7.3642 7.3586 7.2958 7.2764 7.2361 7.0551 7.0362 7.0171 6.9630 6.9437 6.9247 6.9076 6.8885 6.7204 6.7010 6.6828 5.5955 5.5722 2.1349
Me
O
PPh2
OH
3c
1H NMR (400 MHz, DMSO-d6)
0.97
1.03 2.01 2.06 5.98 1.05 1.01 1.04 2.03 1.98
0.98
3.00
10.5 10.0 9.5 9.0 8.5 8.0 7.5 7.0 6.5 6.0 5.5 5.0 4.5 4.0 3.5 3.0 2.5 2.0 1.5 1.0 0.5 0.0
f1 (ppm)

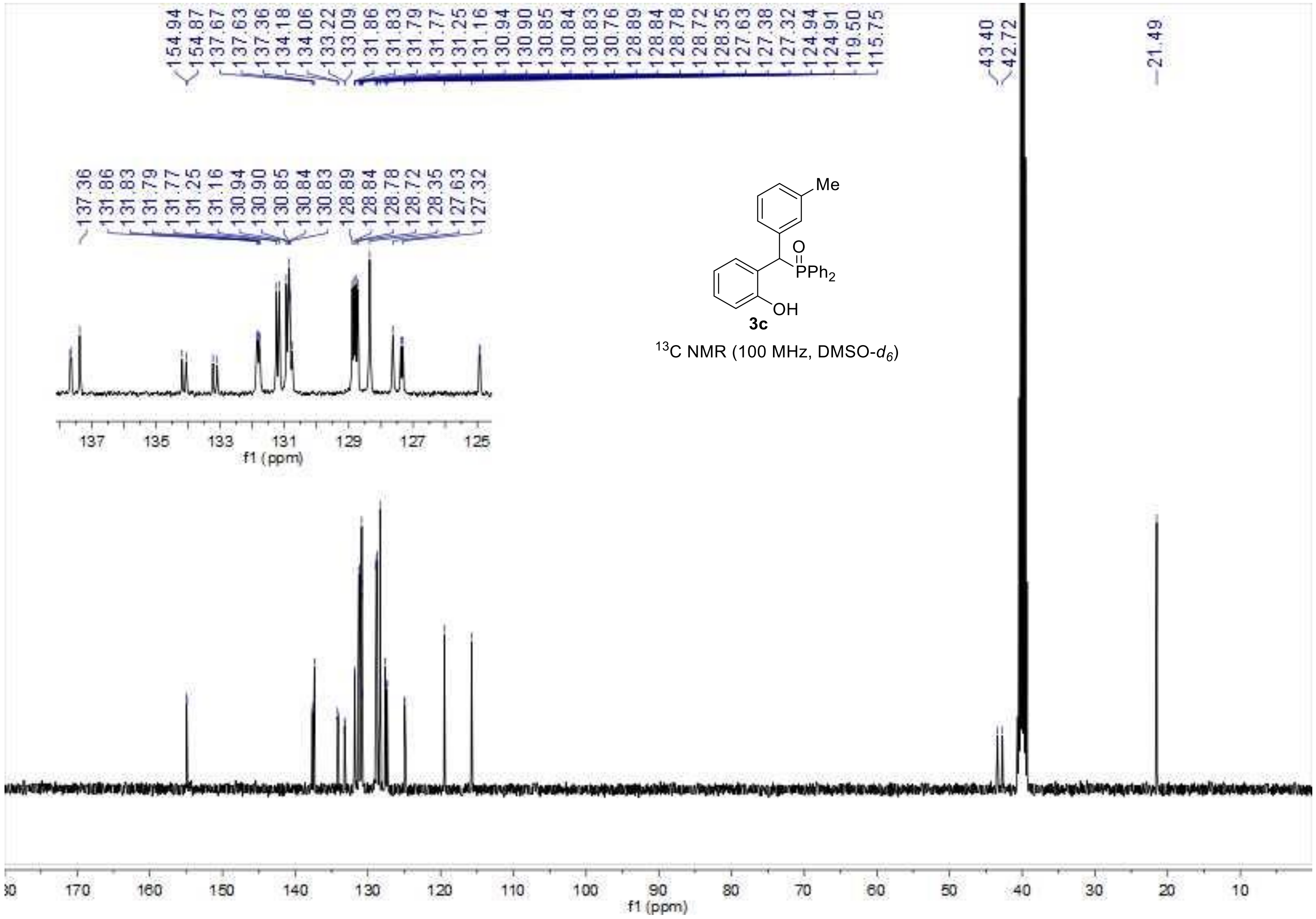

154.94
154.87
137.67
137.63
137.36
134.18
134.06
133.22
133.09
131.86
131.83
131.79
131.77
131.25
131.16
130.94
130.90
130.85
130.84
130.83
130.76
128.89
128.84
128.78
128.72
128.35
127.63
127.38
127.32
124.94
124.91
119.50
115.75
43.40
42.72
21.49
Me
O
PPh2
OH
3c
13C NMR (100 MHz, DMSO-d6)
137 135 133 131 129 127 125
f1 (ppm)
170 160 150 140 130 120 110 100 90 80 70 60 50 40 30 20 10
f1 (ppm)

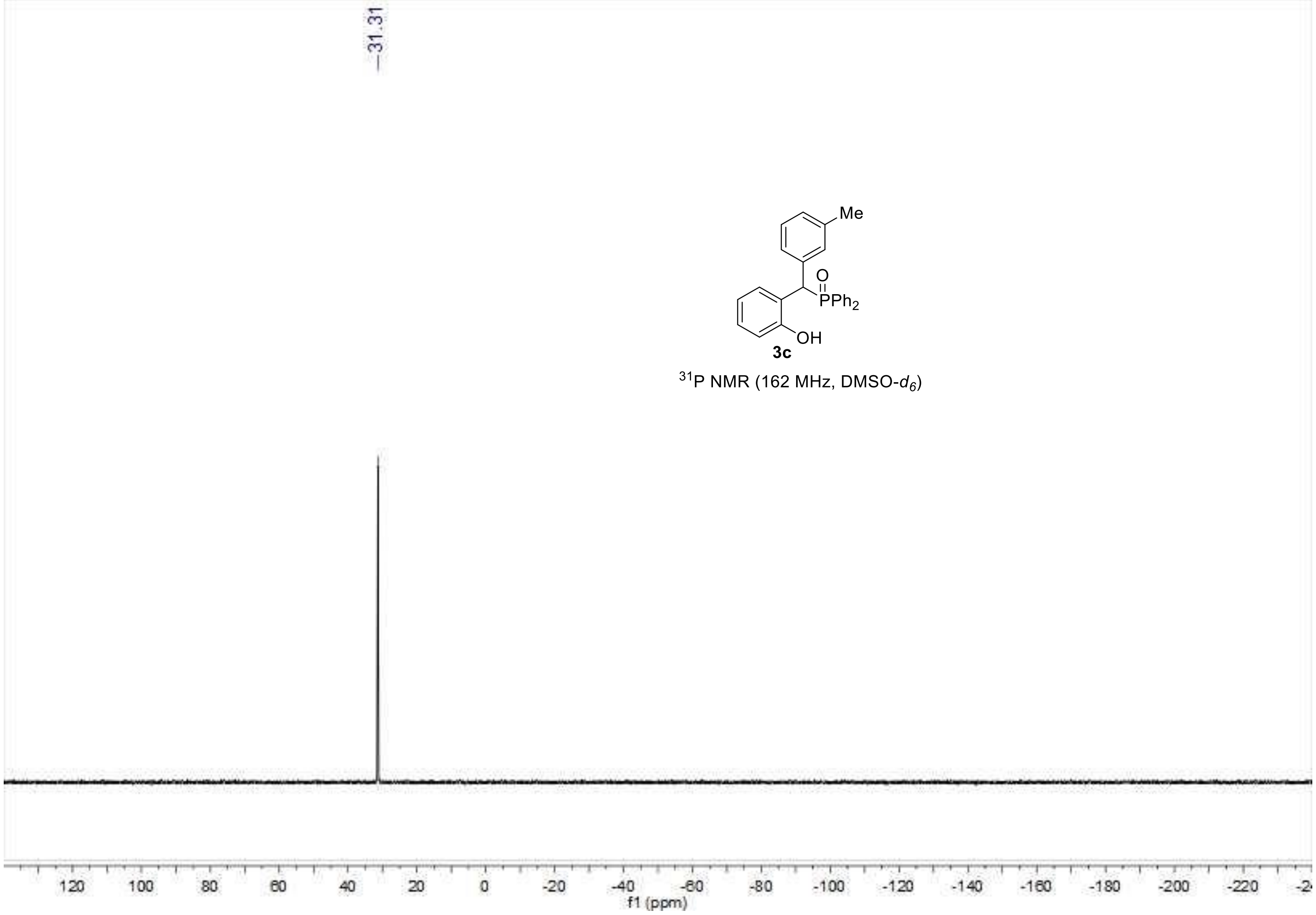

—31.31
Me
O
PPh2
OH
3c
31P NMR (162 MHz, DMSO-d6)
120
100
80
60
40
20
0
-20
-40
-60
-80
-100
-120
-140
-160
-180
-200
-220
f1 (ppm)

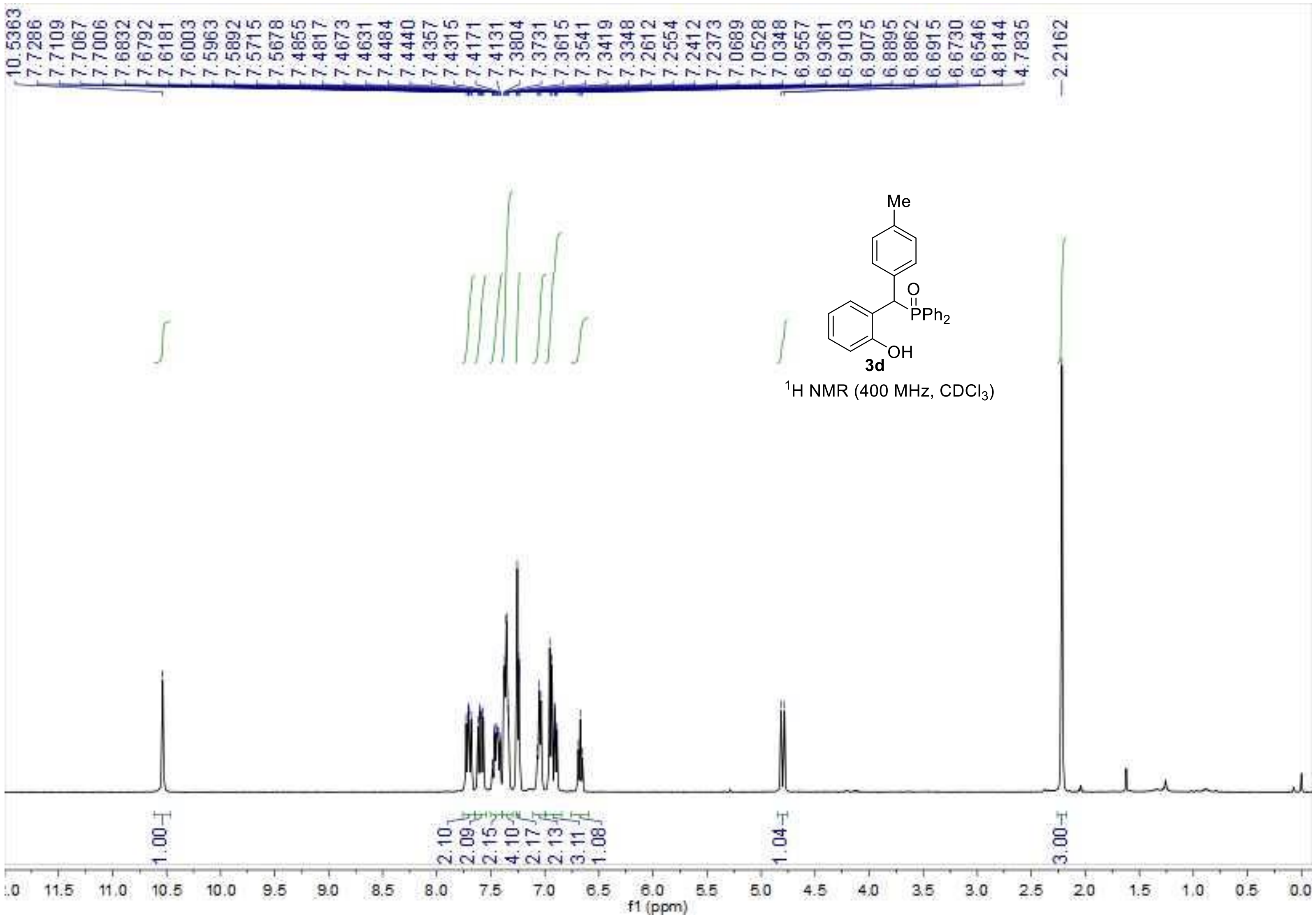

3d
¹H NMR (400 MHz, CDCl₃)
f1 (ppm)

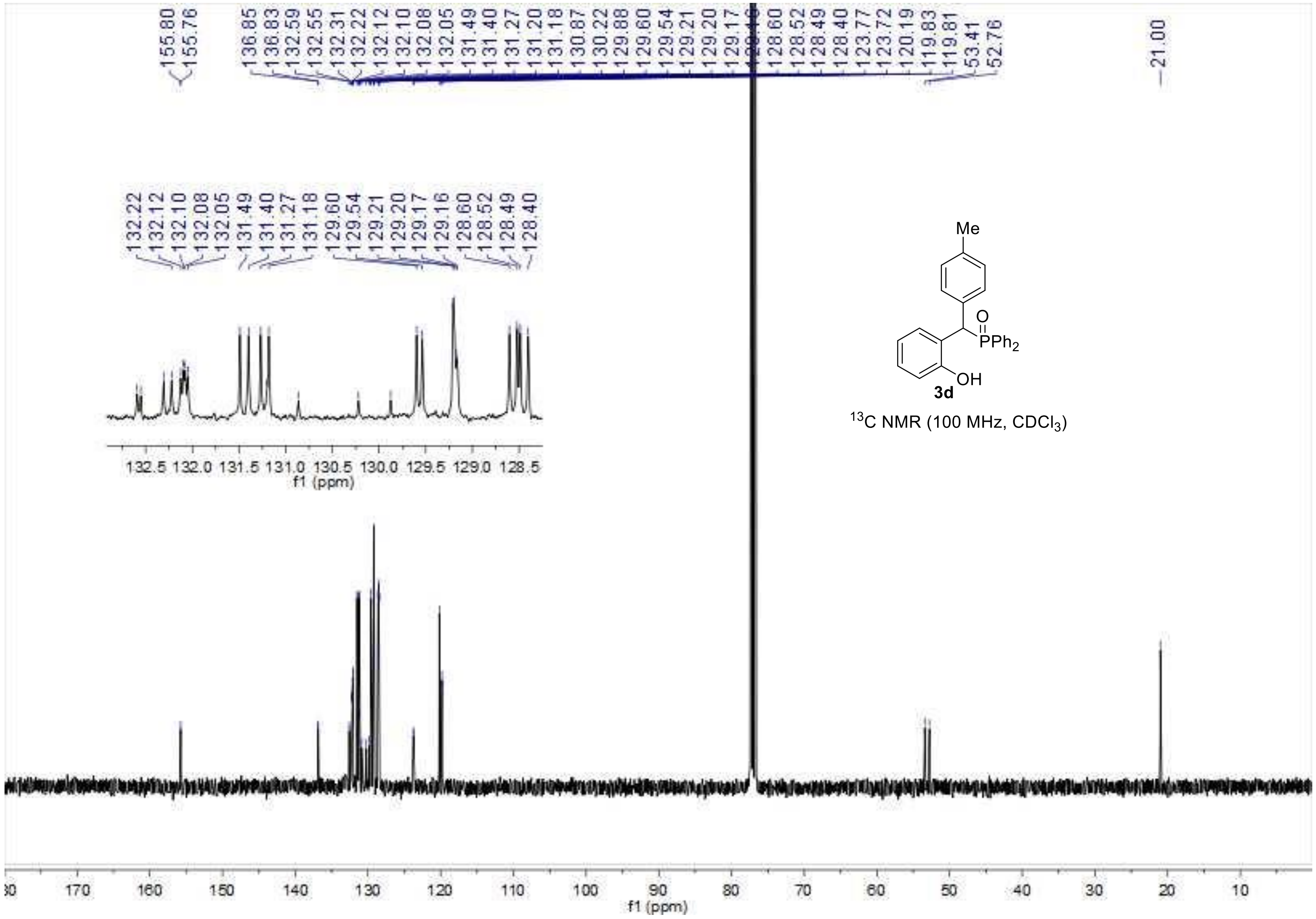

^{13}C NMR (100 MHz, $CDCl_3$)

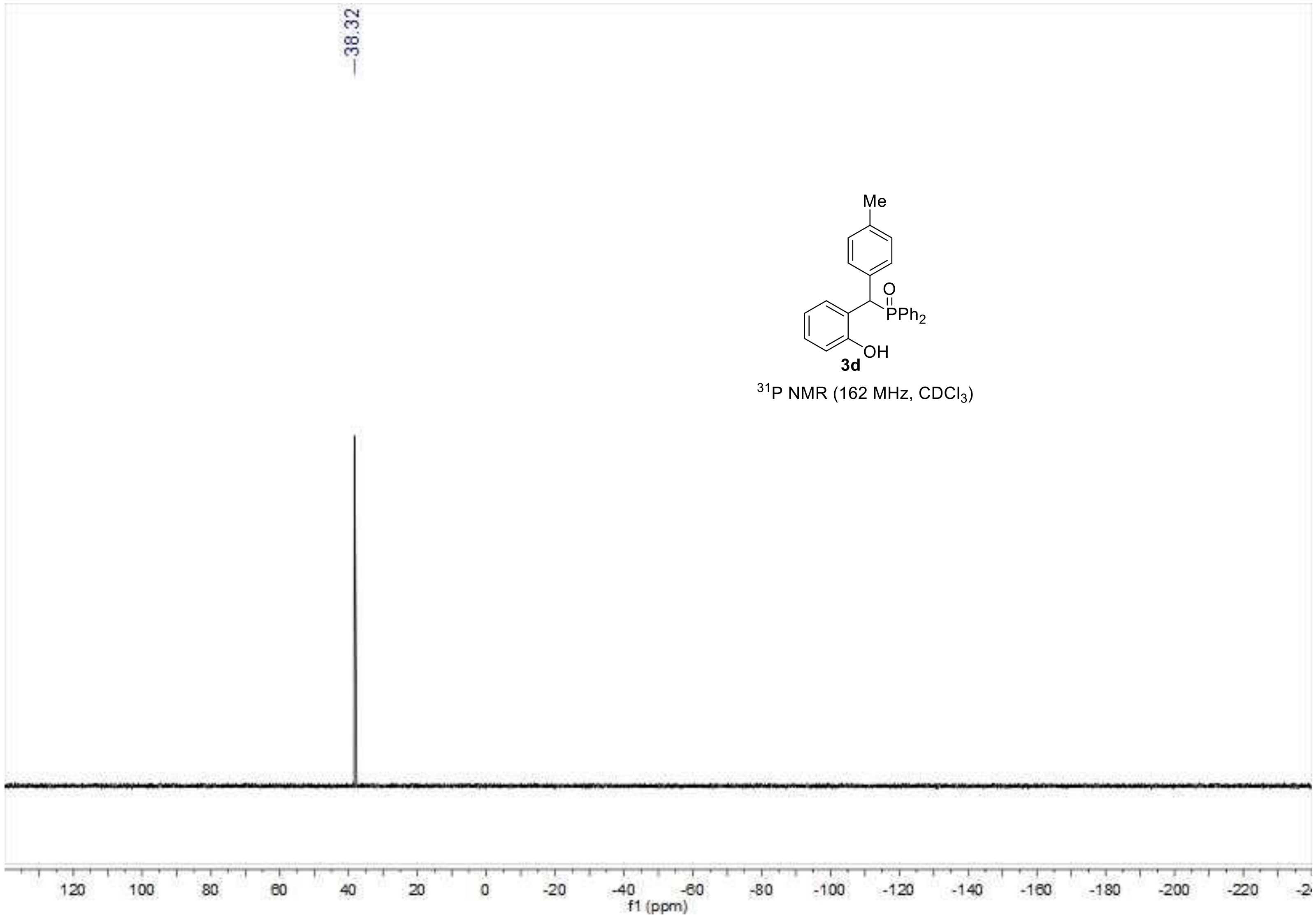
38.32
Me
O
PPh2
OH
3d
31P NMR (162 MHz, CDCl3)
120
100
80
60
40
20
0
-20
-40
-60
-80
-100
-120
-140
-160
-180
-200
-220
f1 (ppm)

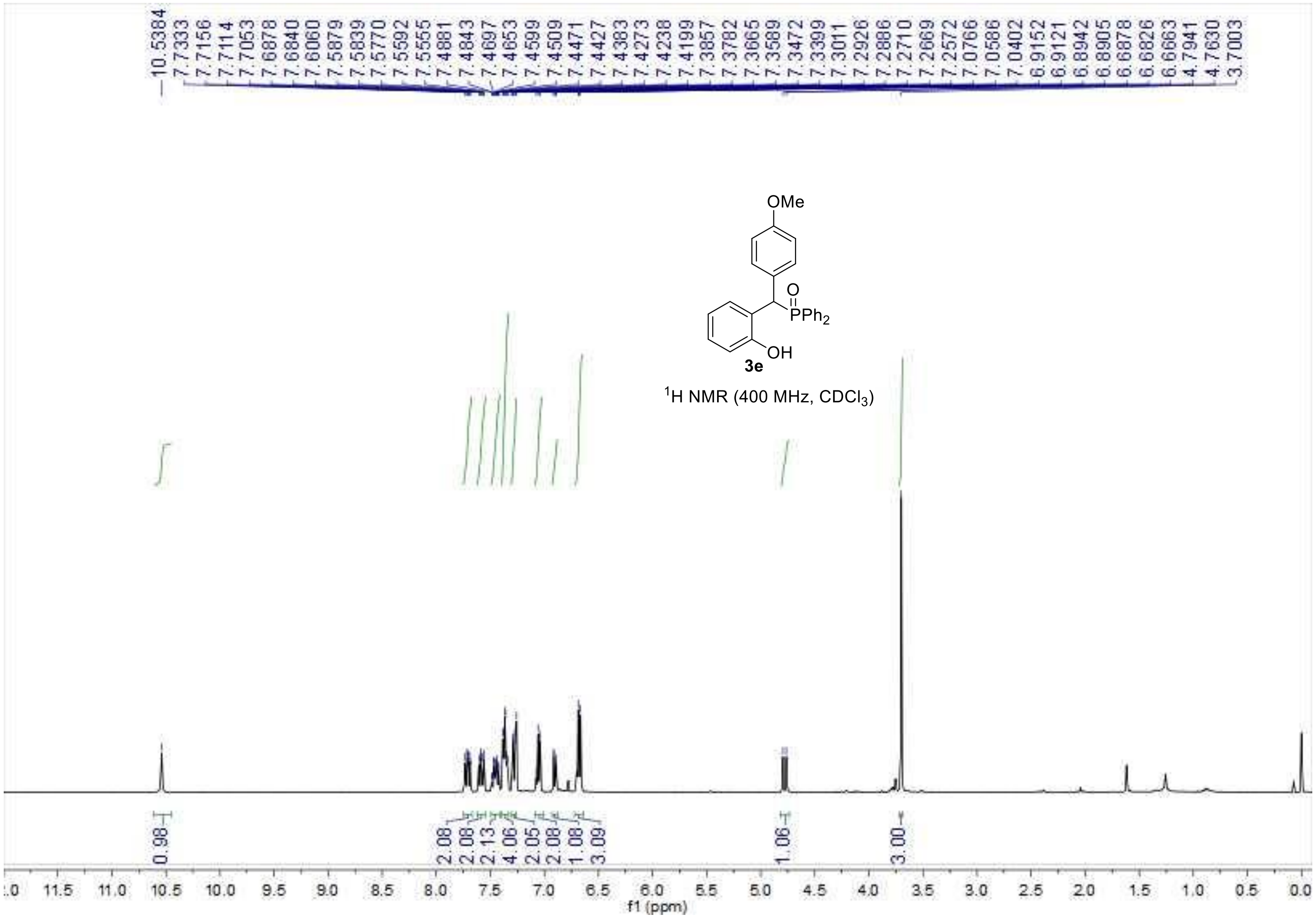
10.5384
7.7333
7.7156
7.7114
7.7053
7.6878
7.6840
7.6060
7.5879
7.5839
7.5770
7.5592
7.5555
7.4881
7.4843
7.4697
7.4653
7.4599
7.4509
7.4471
7.4427
7.4383
7.4273
7.4238
7.4199
7.3857
7.3782
7.3665
7.3589
7.3472
7.3399
7.3011
7.2926
7.2886
7.2710
7.2669
7.2572
7.0766
7.0586
7.0402
6.9152
6.9121
6.8942
6.8905
6.6878
6.6826
6.6663
4.7941
4.7630
3.7003
OMe
O
PPh2
OH
3e
1H NMR (400 MHz, CDCl3)
0.98
2.08
2.08
2.13
4.06
2.05
2.08
1.08
3.09
1.06
3.00
f1 (ppm)

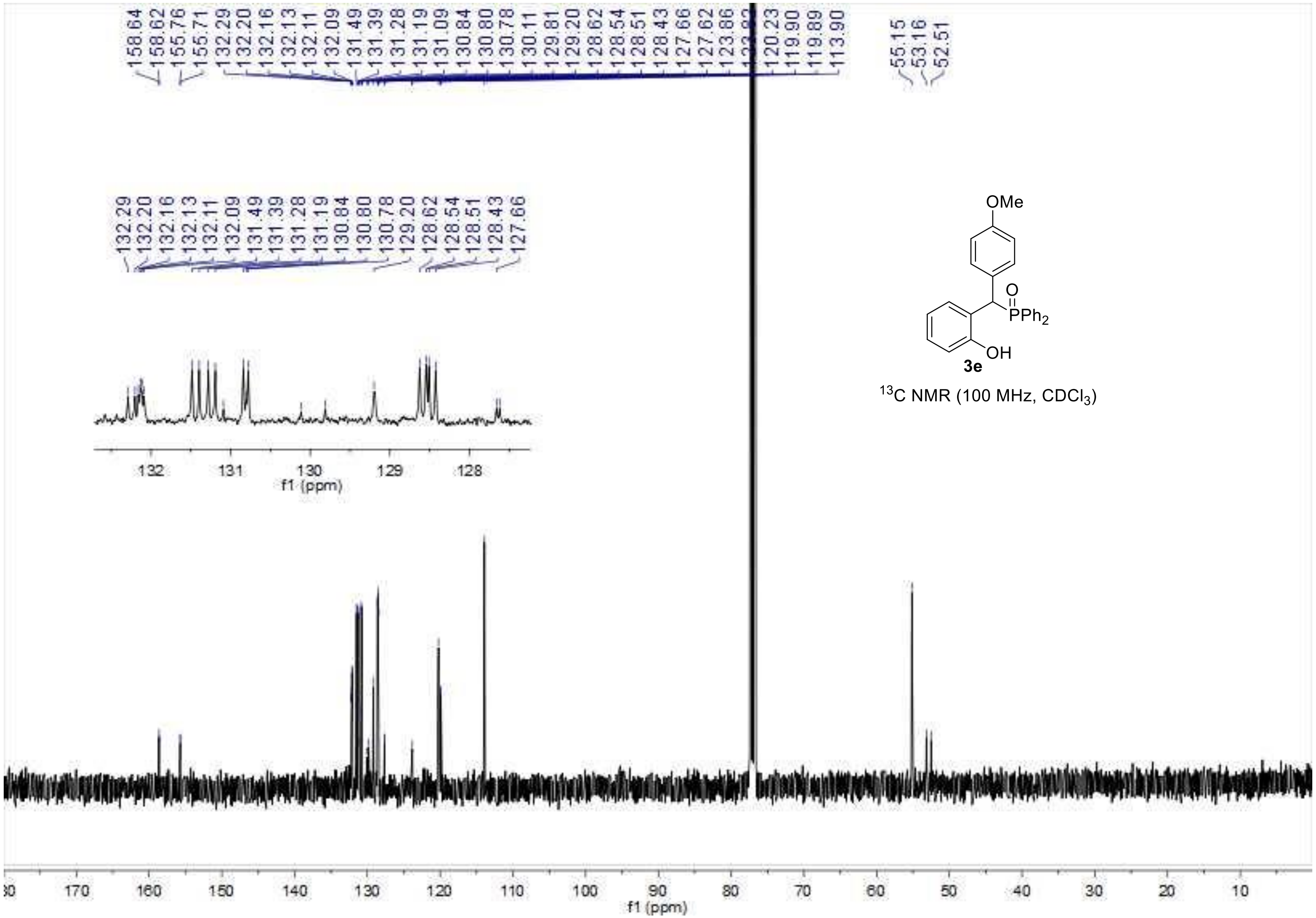

158.64
158.62
155.76
155.71
132.29
132.20
132.16
132.13
132.11
132.09
131.49
131.39
131.28
131.19
131.09
130.84
130.80
130.78
130.11
129.81
129.20
128.62
128.54
128.51
128.43
127.66
127.62
123.86
120.23
119.90
119.89
113.90
55.15
53.16
52.51
OMe
PPh2
OH
3e
^{13}C NMR (100 MHz, $CDCl_3$)
f1 (ppm)

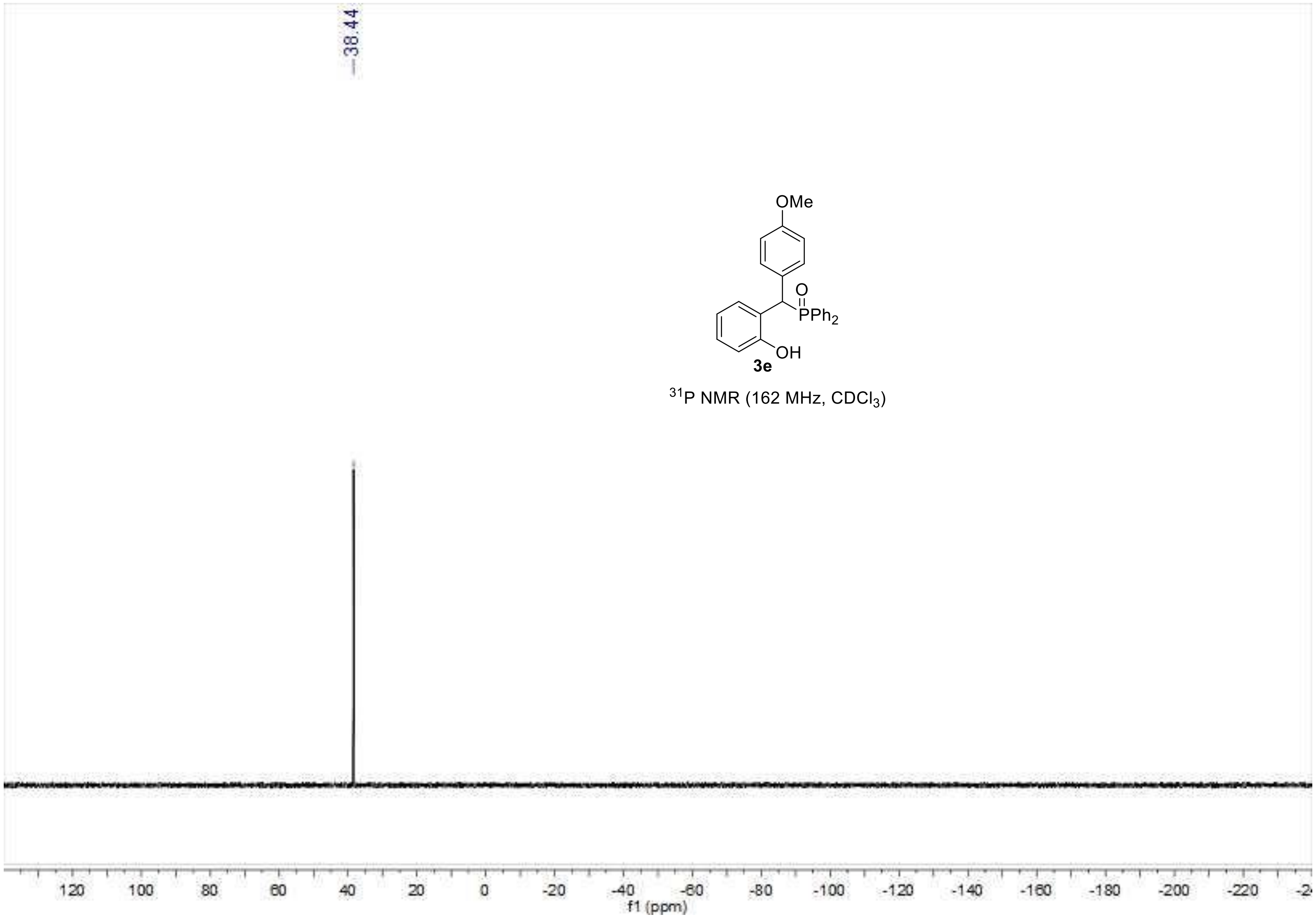
38.44
OMe
O
PPh2
OH
3e
31P NMR (162 MHz, CDCl3)
120 100 80 60 40 20 0 -20 -40 -60 -80 -100 -120 -140 -160 -180 -200 -220
f1 (ppm)

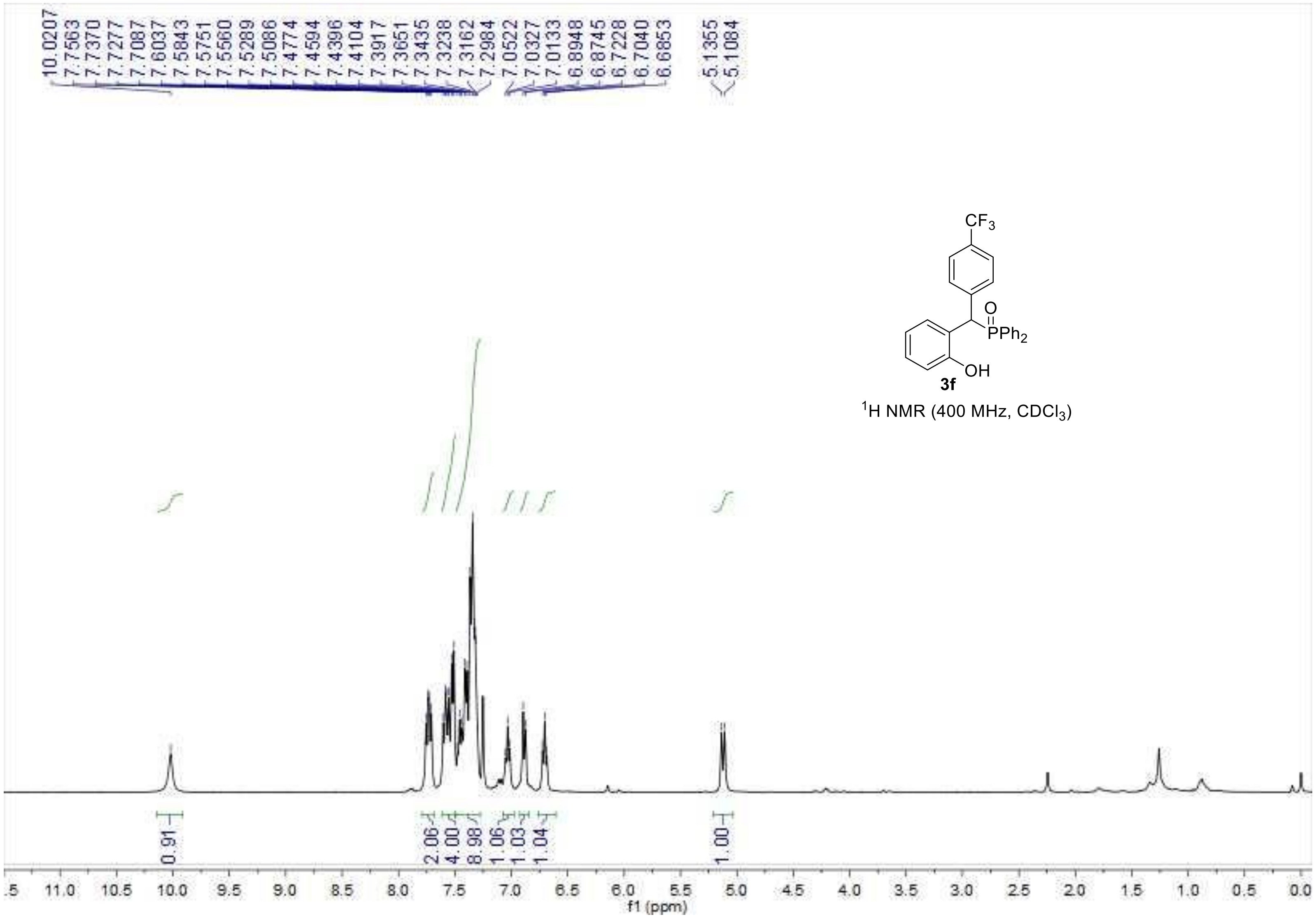
10.0207
7.7563
7.7370
7.7277
7.7087
7.6037
7.5843
7.5751
7.5560
7.5289
7.5086
7.4774
7.4594
7.4396
7.4104
7.3917
7.3651
7.3435
7.3238
7.3162
7.2984
7.0522
7.0327
7.0133
6.8948
6.8745
6.7228
6.7040
6.6853
5.1355
5.1084
CF3
O
PPh2
OH
3f
1H NMR (400 MHz, CDCl3)
0.91
2.06
4.00
8.98
1.06
1.03
1.04
1.00
f1 (ppm)

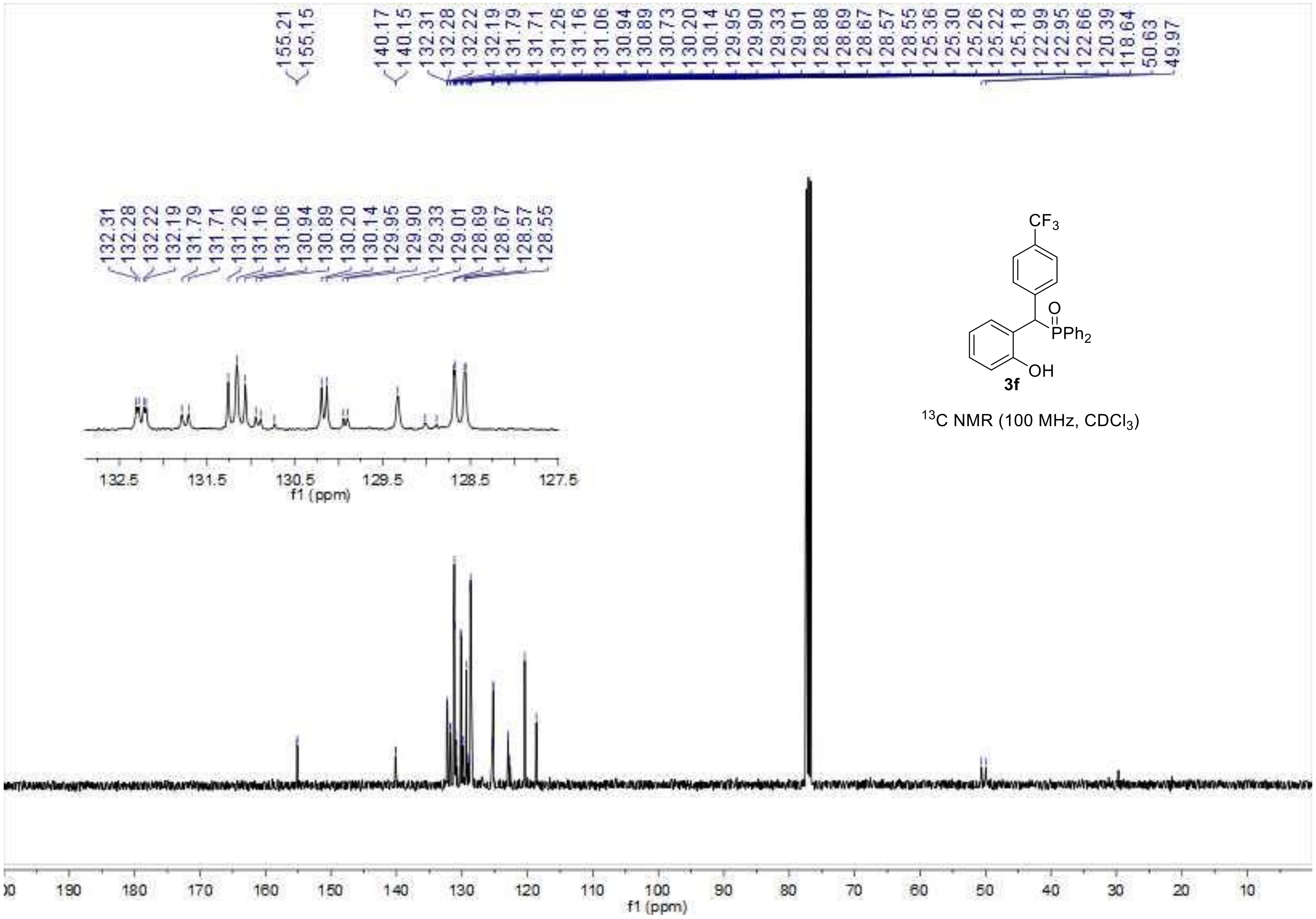
155.21
155.15
140.17
140.15
132.31
132.28
132.22
132.19
131.79
131.71
131.26
131.16
131.06
130.94
130.89
130.73
130.20
130.14
129.95
129.90
129.33
129.01
128.88
128.69
128.67
128.57
128.55
125.36
125.30
125.26
125.22
125.18
122.99
122.95
122.66
120.39
118.64
50.63
49.97
132.5
131.5
130.5
129.5
128.5
127.5
f1 (ppm)
CF_3
O
PPh_2
OH
3f
^{13}C NMR (100 MHz, $CDCl_3$)
190
180
170
160
150
140
130
120
110
100
90
80
70
60
50
40
30
20
10
f1 (ppm)

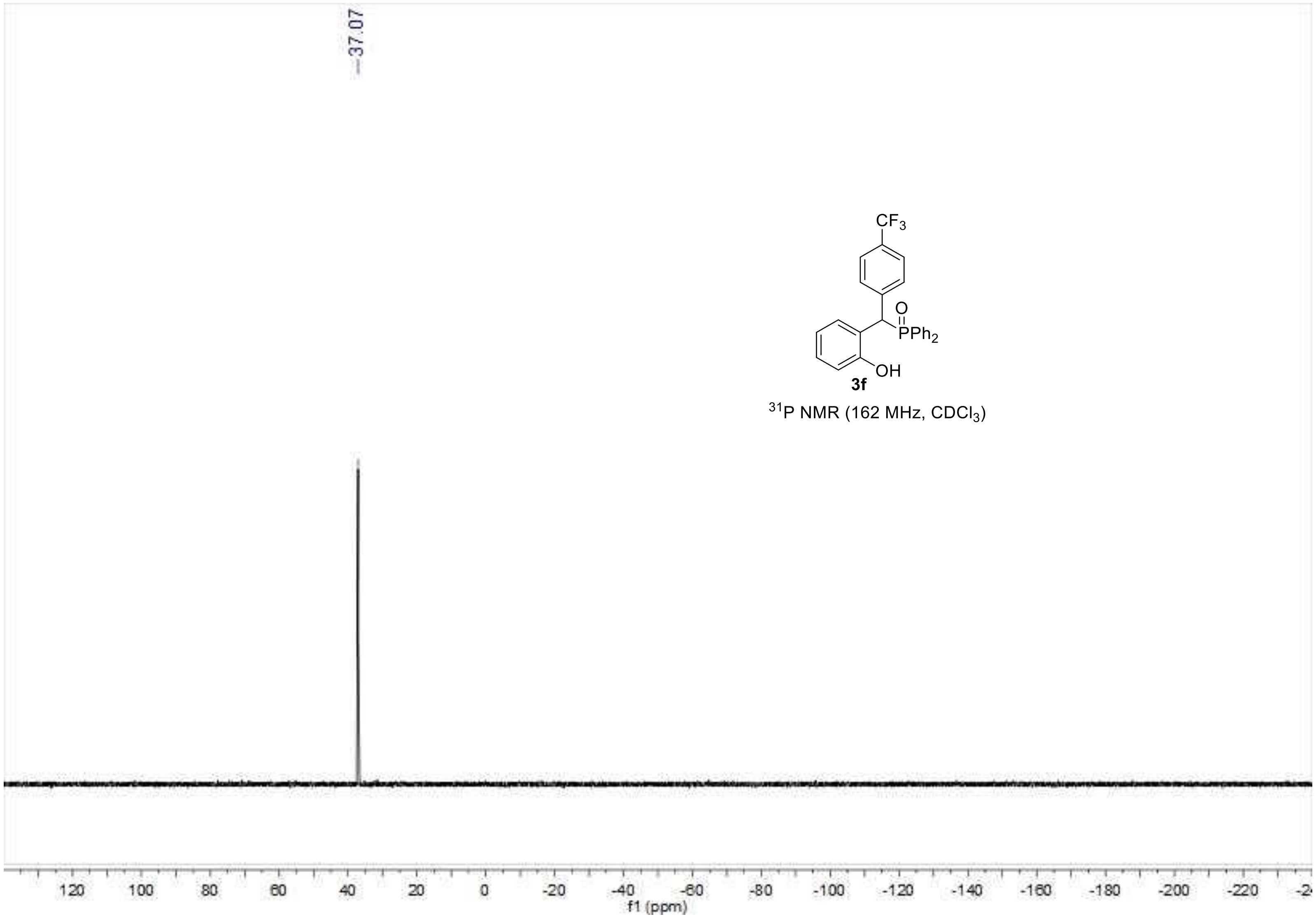

37.07
CF_3
O
PPh_2
OH
3f
^{31}P NMR (162 MHz, $CDCl_3$)
120 100 80 60 40 20 0 -20 -40 -60 -80 -100 -120 -140 -160 -180 -200 -220
f1 (ppm)

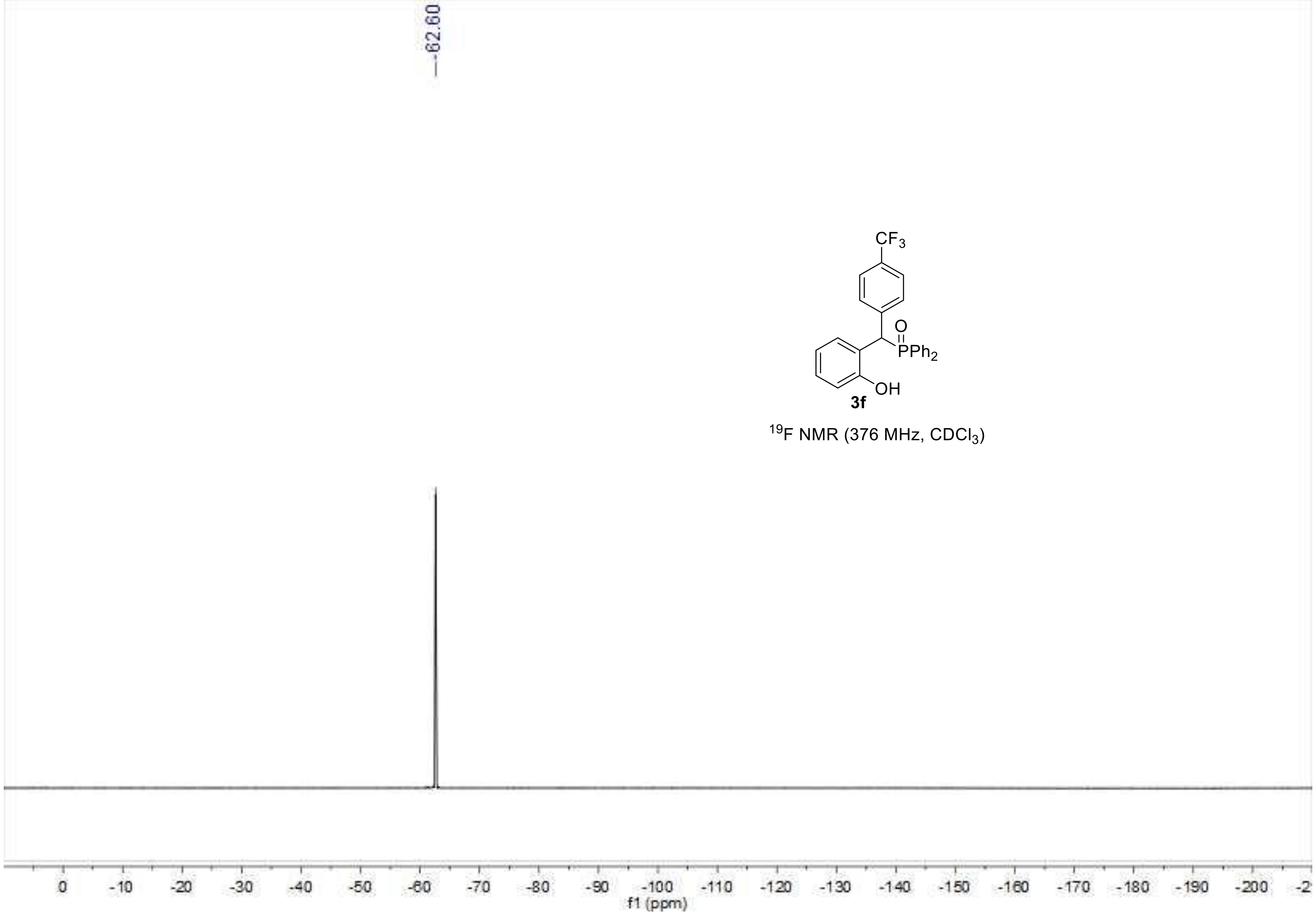

-62.60
CF3
O
PPh2
OH
3f
19F NMR (376 MHz, CDCl3)
0 -10 -20 -30 -40 -50 -60 -70 -80 -90 -100 -110 -120 -130 -140 -150 -160 -170 -180 -190 -200
f1 (ppm)

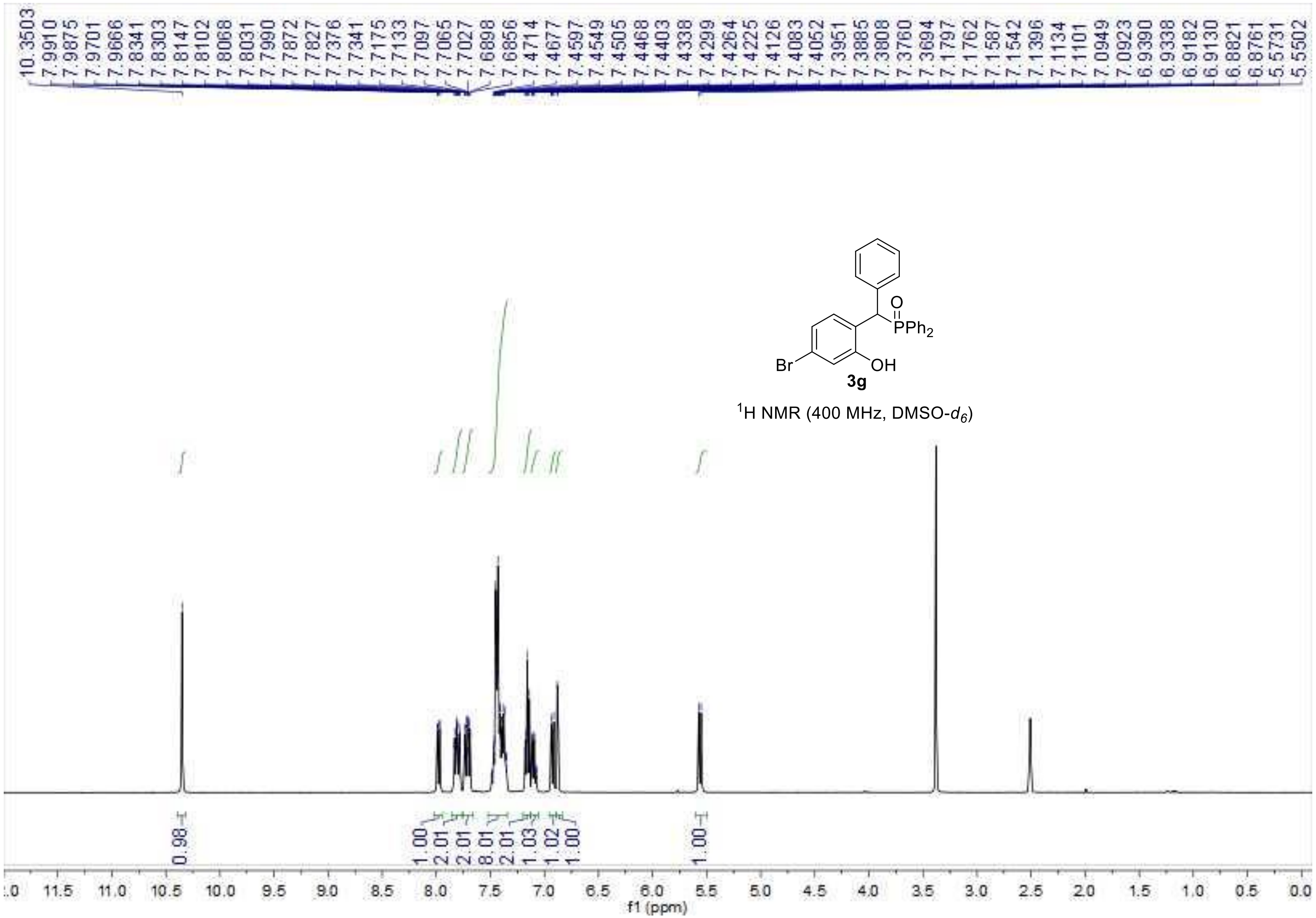
10.3503
7.9910
7.9875
7.9701
7.9666
7.8341
7.8303
7.8147
7.8102
7.8068
7.8031
7.7990
7.7872
7.7827
7.7376
7.7341
7.7175
7.7133
7.7097
7.7065
7.7027
7.6898
7.6856
7.4714
7.4677
7.4597
7.4549
7.4505
7.4468
7.4403
7.4338
7.4299
7.4264
7.4225
7.4126
7.4083
7.4052
7.3951
7.3885
7.3808
7.3760
7.3694
7.1797
7.1762
7.1587
7.1542
7.1396
7.1134
7.1101
7.0949
7.0923
6.9390
6.9338
6.9182
6.9130
6.8821
6.8761
5.5731
5.5502
O
PPh2
Br
OH
3g
1H NMR (400 MHz, DMSO-d6)
0.98
1.00
2.01
2.01
8.01
2.01
1.03
1.02
1.00
1.00
f1 (ppm)

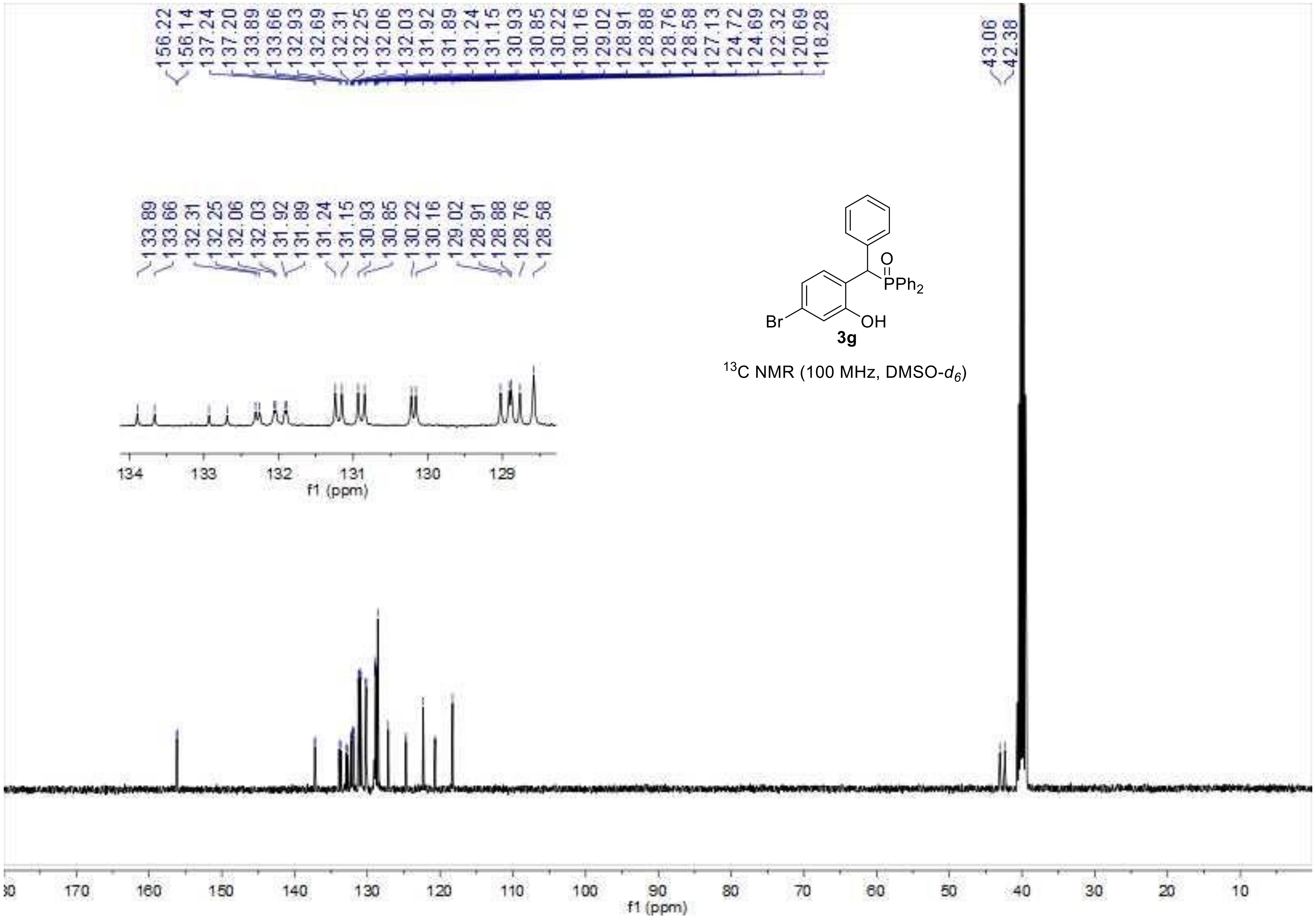
156.22
156.14
137.24
137.20
133.89
133.66
132.93
132.69
132.31
132.25
132.06
132.03
131.92
131.89
131.24
131.15
130.93
130.85
130.22
130.16
129.02
128.91
128.88
128.76
128.58
127.13
124.72
124.69
122.32
120.69
118.28
43.06
42.38
133.89
133.66
132.31
132.25
132.06
132.03
131.92
131.89
131.24
131.15
130.93
130.85
130.22
130.16
129.02
128.91
128.88
128.76
128.58
134
133
132
131
130
129
f1 (ppm)
O
PPh2
Br
OH
3g
^{13}C NMR (100 MHz, DMSO-d_6)
170
160
150
140
130
120
110
100
90
80
70
60
50
40
30
20
10
f1 (ppm)

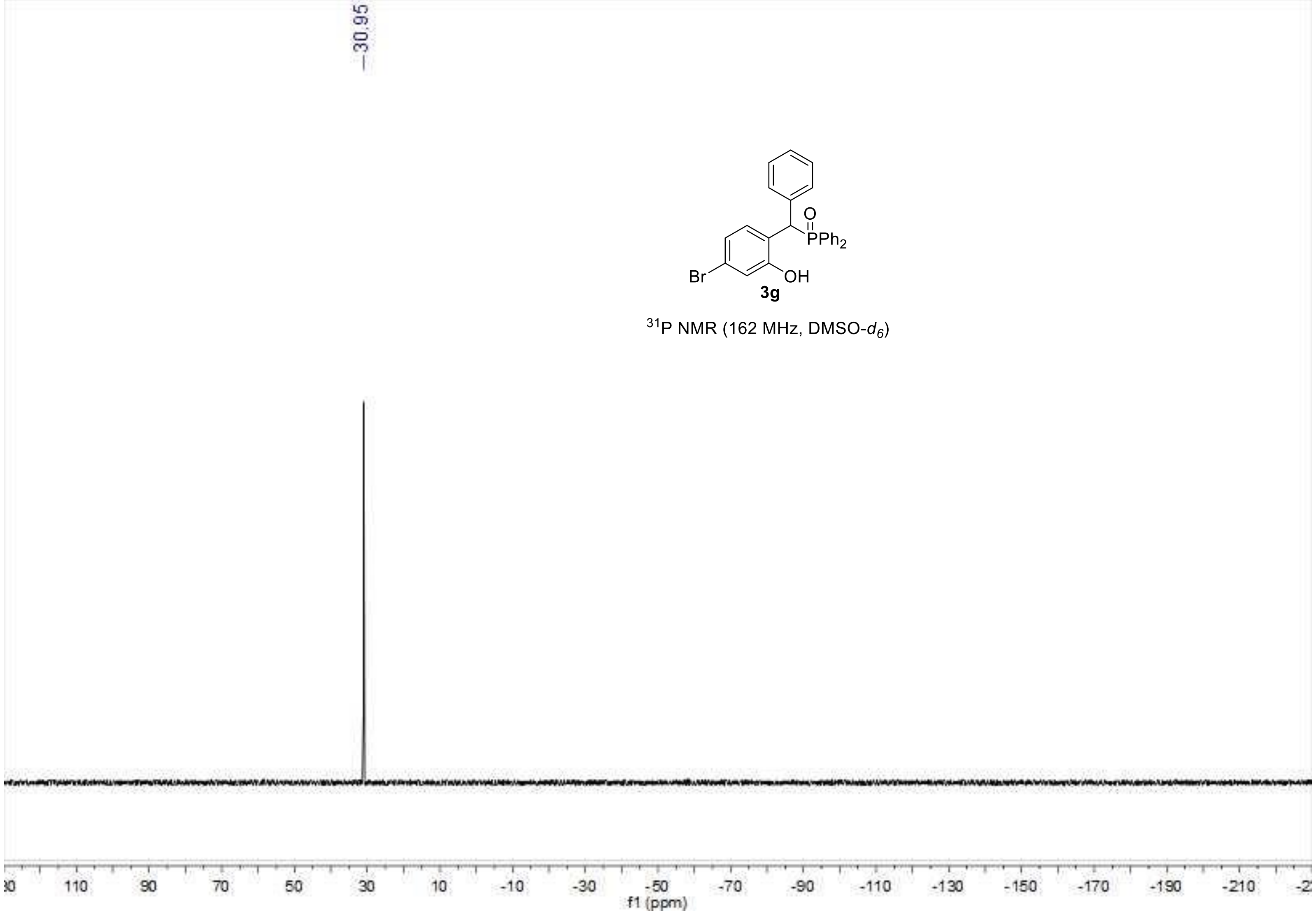
30.95
Br
OH
O
PPh2
3g
31P NMR (162 MHz, DMSO-d6)
110
90
70
50
30
10
-10
-30
-50
-70
-90
-110
-130
-150
-170
-190
-210
f1 (ppm)

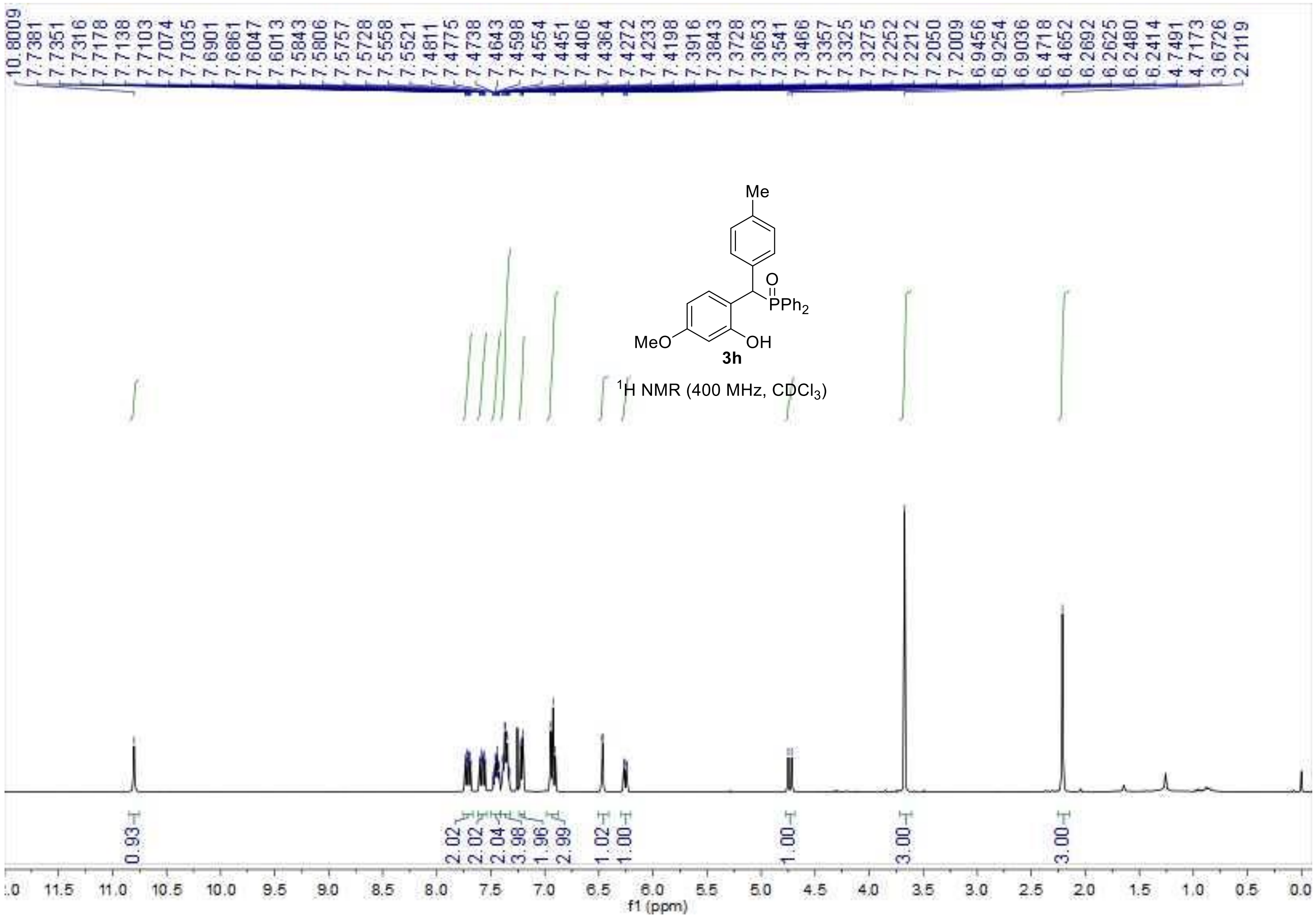

10.8009
7.7381
7.7351
7.7316
7.7178
7.7138
7.7103
7.7074
7.7035
7.6901
7.6861
7.6047
7.6013
7.5843
7.5806
7.5757
7.5728
7.5558
7.5521
7.4811
7.4775
7.4738
7.4643
7.4598
7.4554
7.4451
7.4406
7.4364
7.4272
7.4233
7.4198
7.3916
7.3843
7.3728
7.3653
7.3541
7.3466
7.3357
7.3325
7.3275
7.2252
7.2212
7.2050
7.2009
6.9456
6.9254
6.9036
6.4718
6.4652
6.2692
6.2625
6.2480
6.2414
4.7491
4.7173
3.6726
2.2119
Me
O
PPh2
MeO
OH
3h
1H NMR (400 MHz, CDCl3)
0.93
2.02
2.02
2.04
3.98
1.96
2.99
1.02
1.00
1.00
3.00
3.00
f1 (ppm)

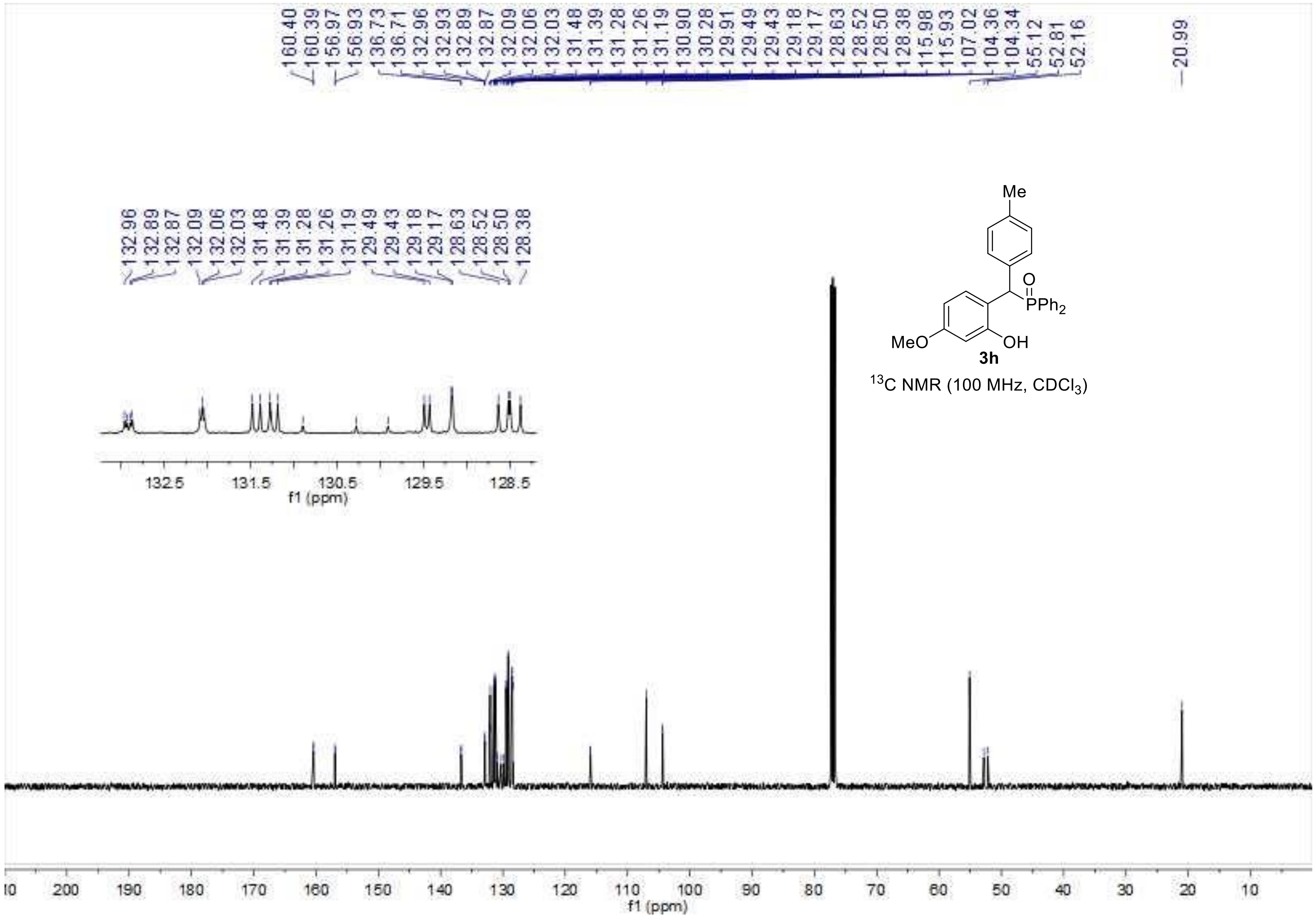
160.40
160.39
156.97
156.93
136.73
136.71
132.96
132.93
132.89
132.87
132.09
132.06
132.03
131.48
131.39
131.28
131.26
131.19
130.90
130.28
129.91
129.49
129.43
129.18
129.17
128.63
128.52
128.50
128.38
115.98
115.93
107.02
104.36
104.34
55.12
52.81
52.16
20.99
Me
O
PPh2
MeO
OH
3h
13C NMR (100 MHz, CDCl3)
f1 (ppm)

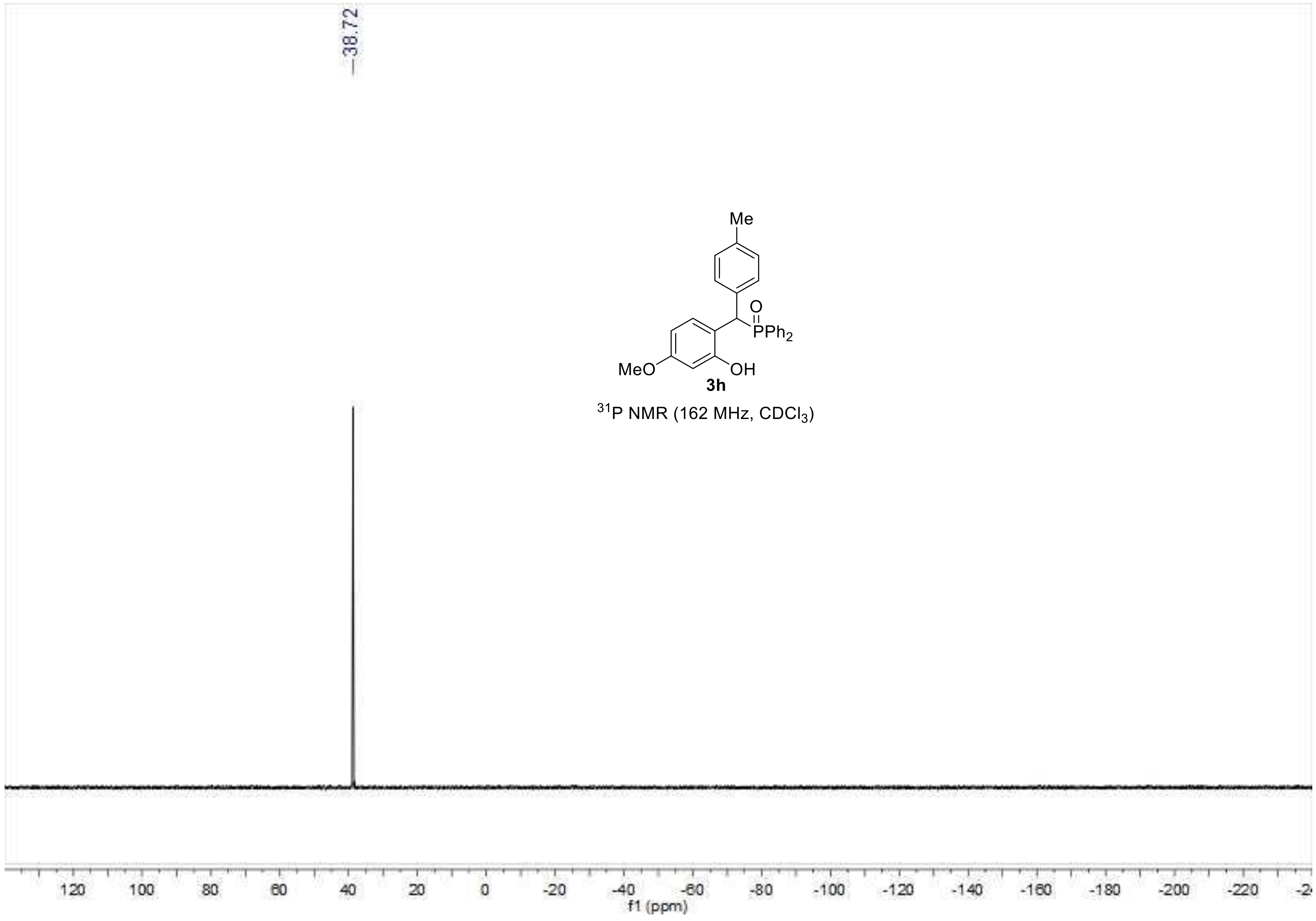

—38.72
Me
O
PPh2
MeO
OH
3h
31P NMR (162 MHz, CDCl3)
120 100 80 60 40 20 0 -20 -40 -60 -80 -100 -120 -140 -160 -180 -200 -220
f1 (ppm)

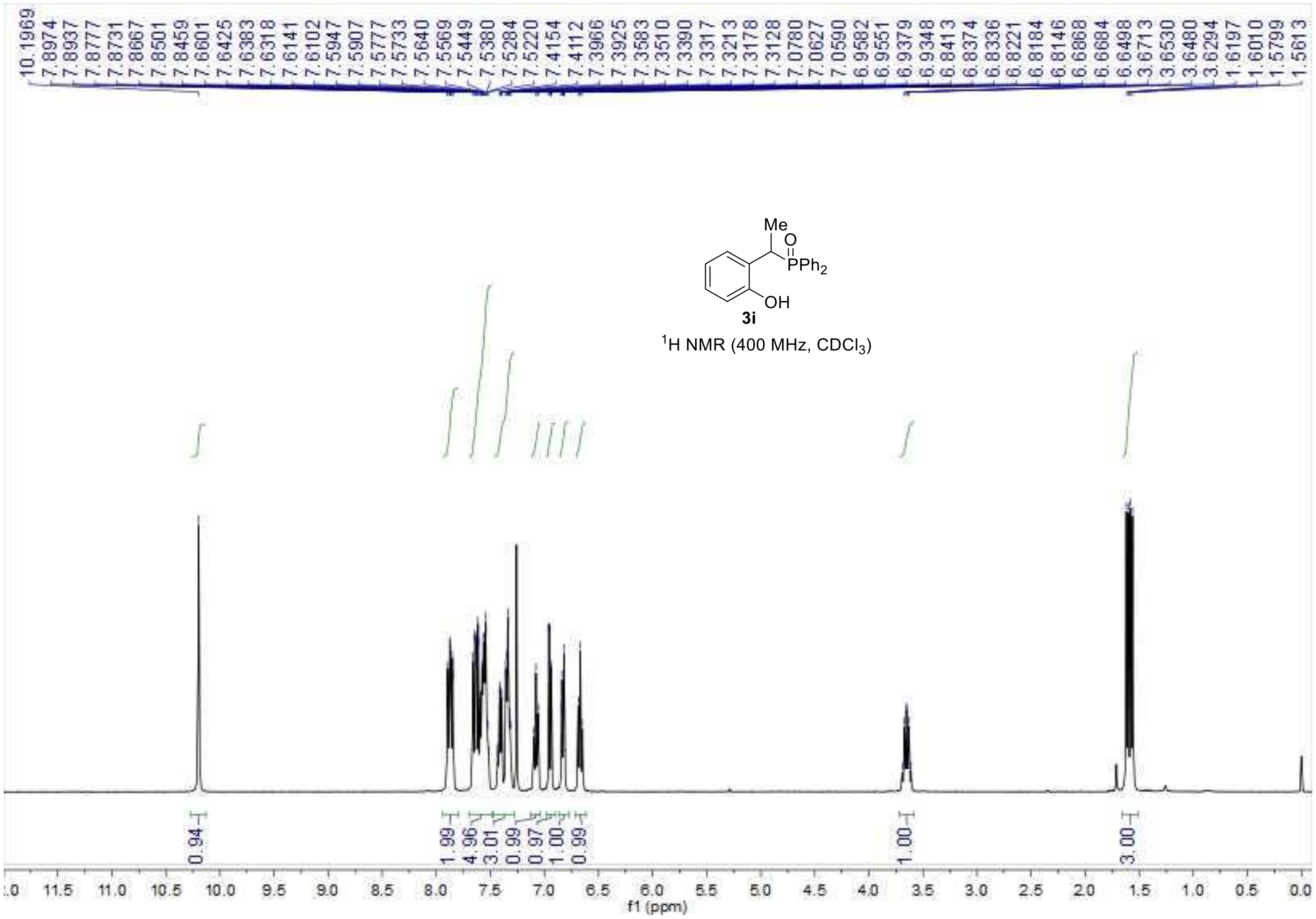

10.1969
7.8974
7.8937
7.8777
7.8731
7.8667
7.8501
7.8459
7.6601
7.6425
7.6383
7.6318
7.6141
7.6102
7.5947
7.5907
7.5777
7.5733
7.5640
7.5569
7.5449
7.5380
7.5284
7.5220
7.4154
7.4112
7.3966
7.3925
7.3583
7.3510
7.3390
7.3317
7.3213
7.3178
7.3128
7.0780
7.0627
7.0590
6.9582
6.9551
6.9379
6.9348
6.8413
6.8374
6.8336
6.8221
6.8184
6.8146
6.6868
6.6684
6.6498
3.6713
3.6530
3.6480
3.6294
1.6197
1.6010
1.5799
1.5613
Me
O
PPh2
OH
3i
1H NMR (400 MHz, CDCl3)
0.94
1.99
4.96
3.01
0.99
0.97
1.00
0.99
1.00
3.00
11.5 11.0 10.5 10.0 9.5 9.0 8.5 8.0 7.5 7.0 6.5 6.0 5.5 5.0 4.5 4.0 3.5 3.0 2.5 2.0 1.5 1.0 0.5 0.0
f1 (ppm)

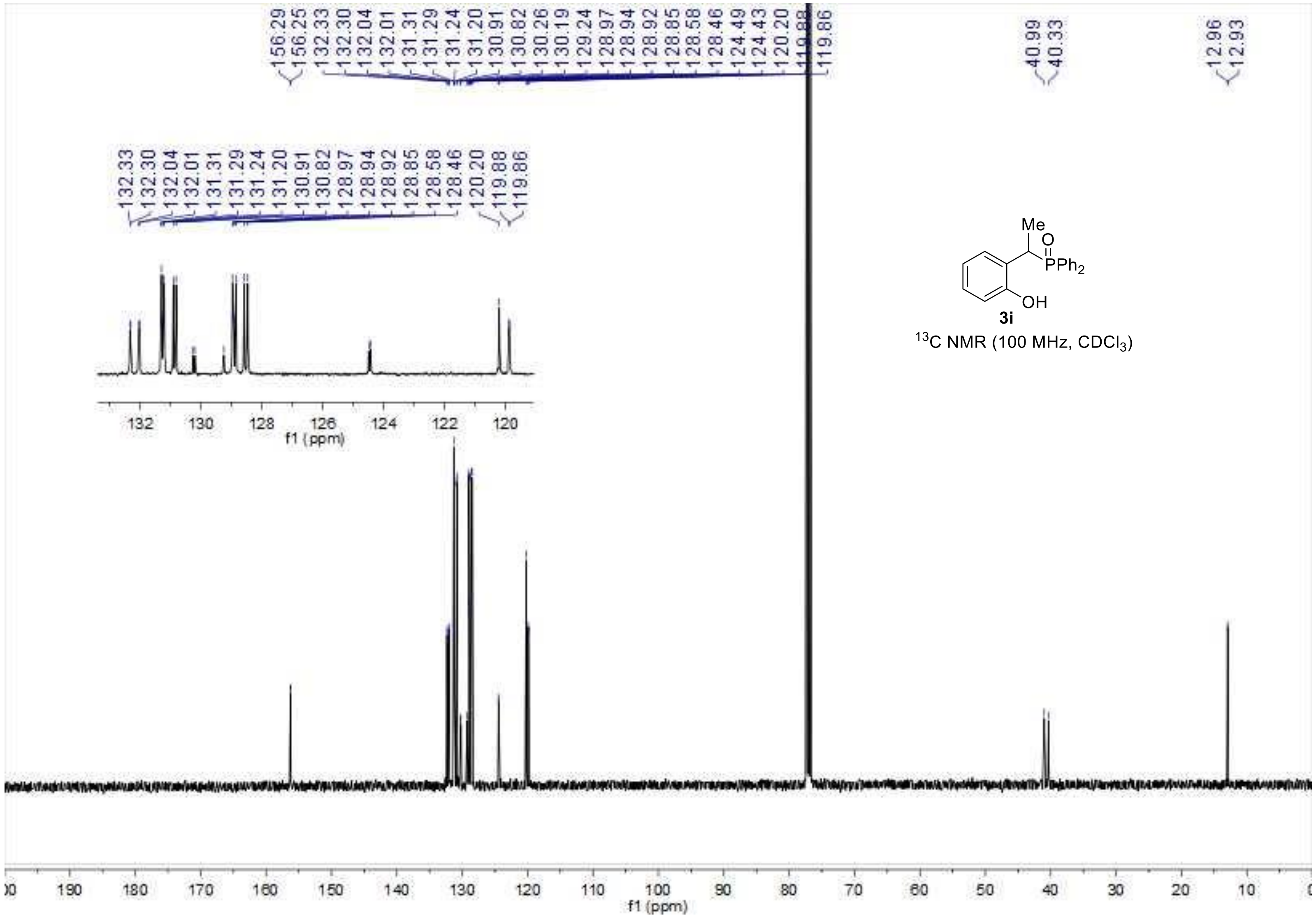
156.29
156.25
132.33
132.30
132.04
132.01
131.31
131.29
131.24
131.20
130.91
130.82
130.26
130.19
129.24
128.97
128.94
128.92
128.85
128.58
128.46
124.49
124.43
120.20
119.88
119.86
40.99
40.33
12.96
12.93
Me
O
PPh2
OH
3i
13C NMR (100 MHz, CDCl3)
f1 (ppm)

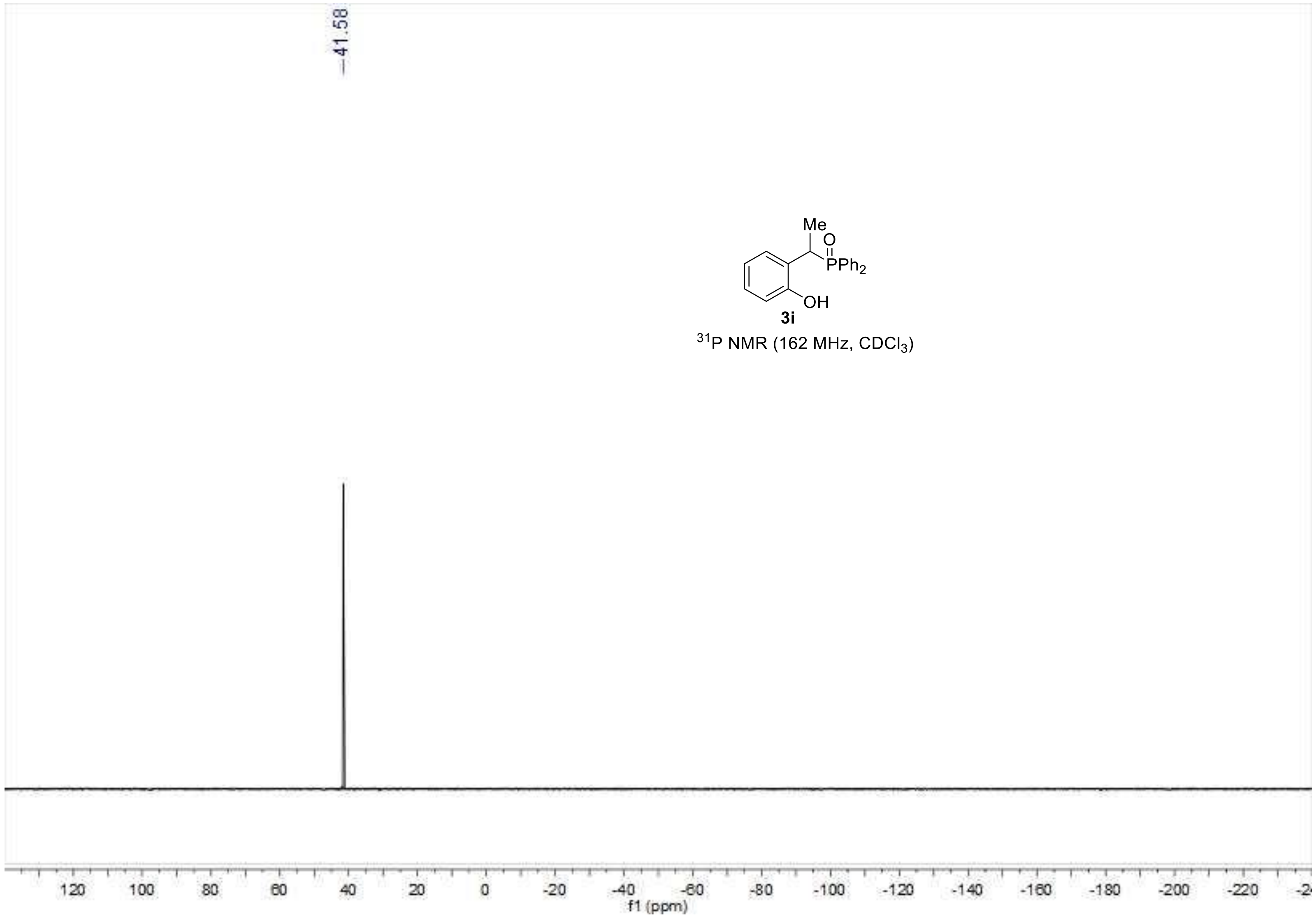

41.58
Me
O
PPh2
OH
3i
31P NMR (162 MHz, CDCl3)
120
100
80
60
40
20
0
-20
-40
-60
-80
-100
-120
-140
-160
-180
-200
-220
f1 (ppm)

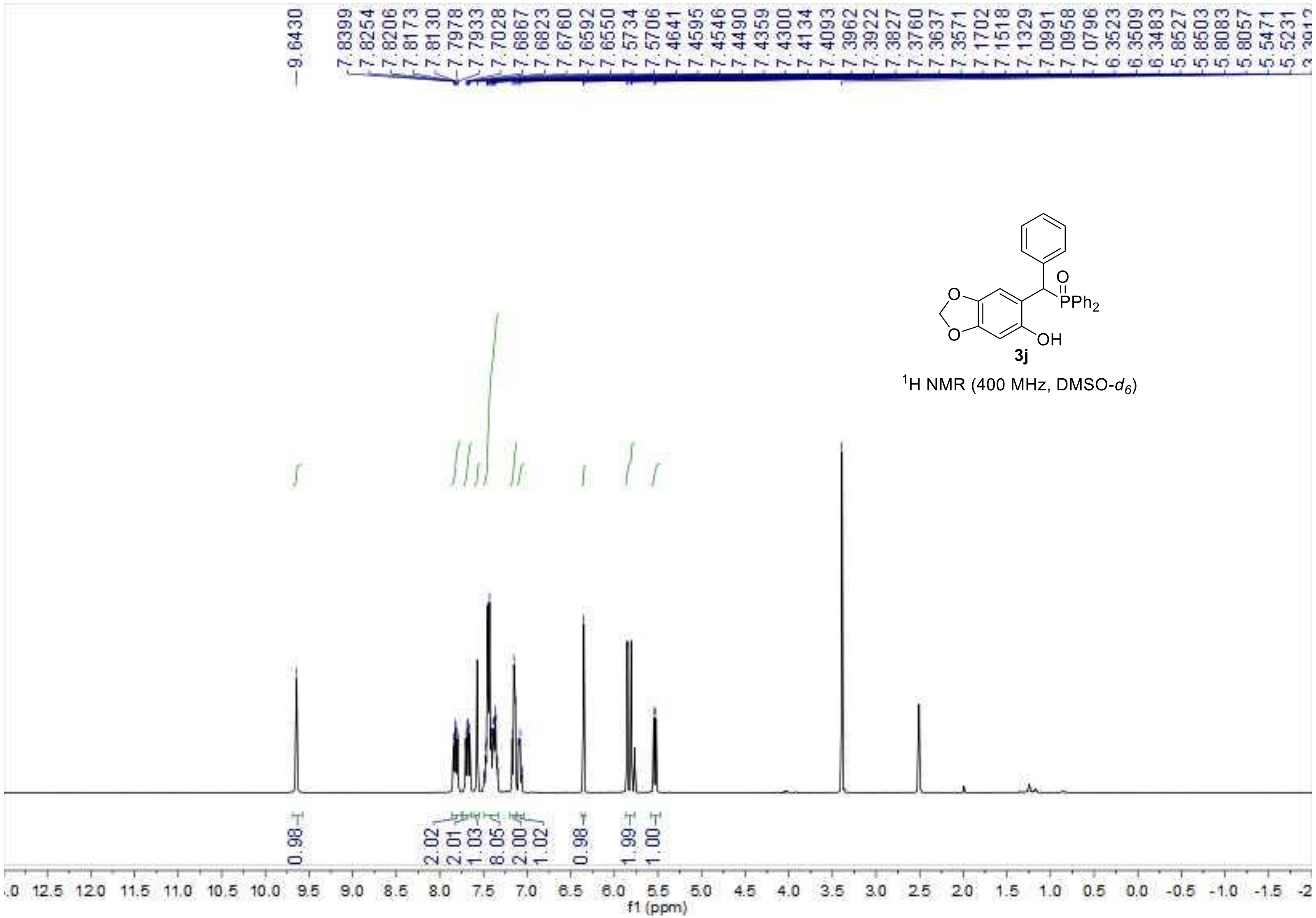

9.6430
7.8399 7.8254 7.8206 7.8173 7.8130 7.7978 7.7933 7.7028 7.6867 7.6823 7.6760 7.6592 7.6550 7.5734 7.5706 7.4641 7.4595 7.4546 7.4490 7.4359 7.4300 7.4134 7.4093 7.3962 7.3922 7.3827 7.3760 7.3637 7.3571 7.1702 7.1518 7.1329 7.0991 7.0958 7.0796
6.3523 6.3509 6.3483
5.8527 5.8503 5.8083 5.8057 5.5471 5.5231
O
PPh2
O
O
OH
3j
1H NMR (400 MHz, DMSO-d6)
0.98
2.02 2.01 1.03 8.05 2.00 1.02
0.98
1.99 1.00
12.5 12.0 11.5 11.0 10.5 10.0 9.5 9.0 8.5 8.0 7.5 7.0 6.5 6.0 5.5 5.0 4.5 4.0 3.5 3.0 2.5 2.0 1.5 1.0 0.5 0.0 -0.5 -1.0 -1.5
f1 (ppm)

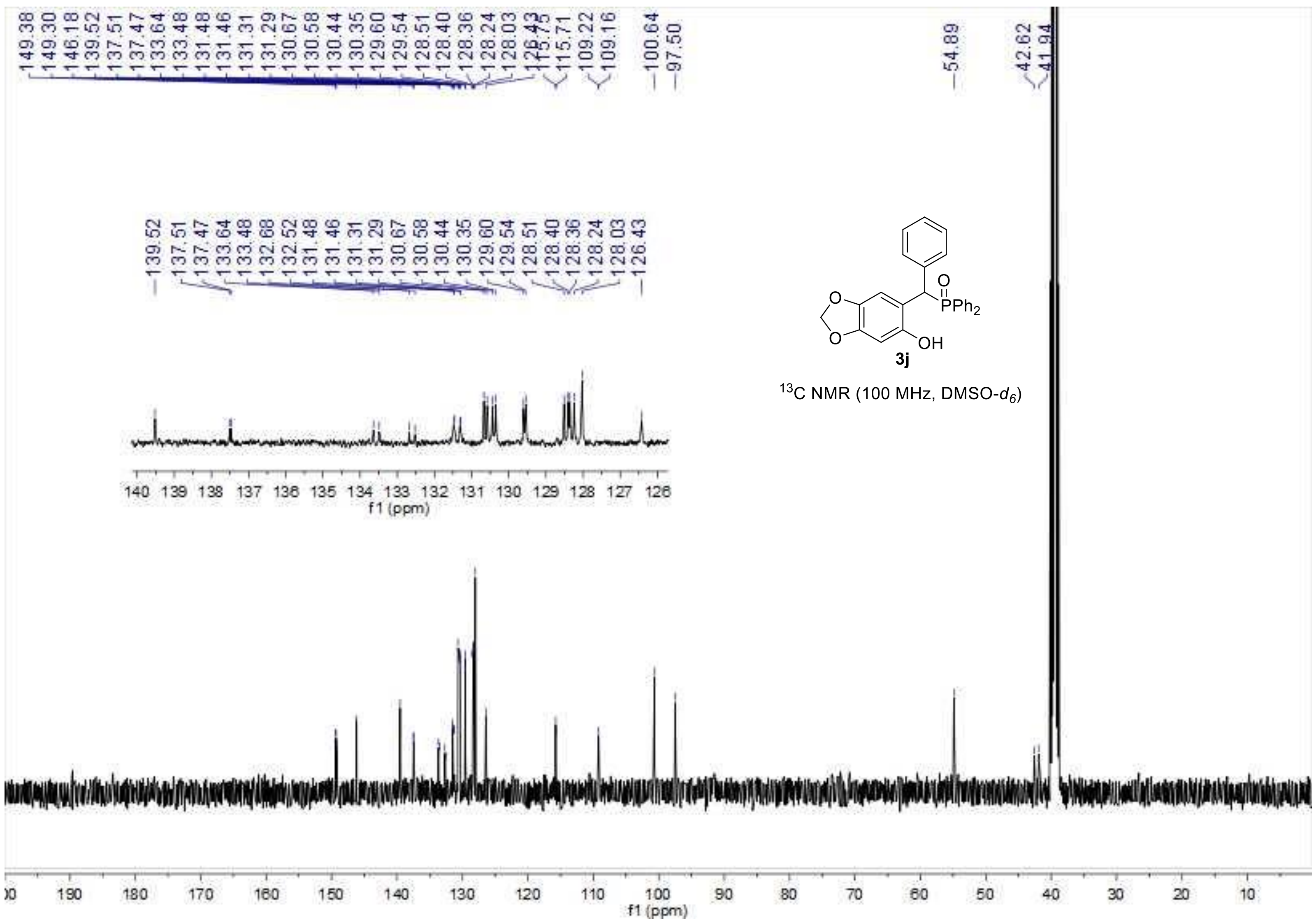

3j
^{13}C NMR (100 MHz, DMSO-d_6)
OH
PPh2
O
f1 (ppm)

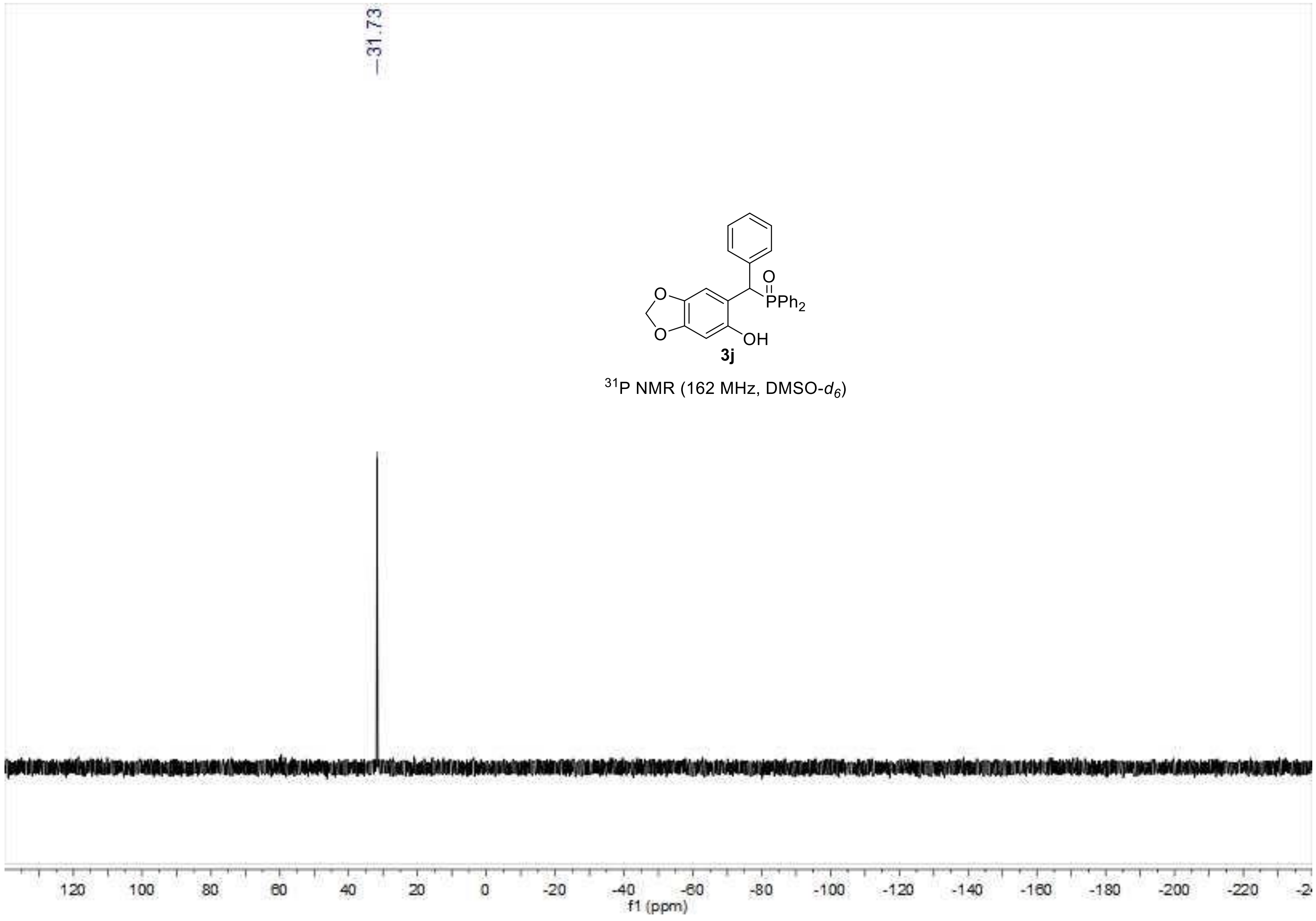

31.73
3j
^{31}P NMR (162 MHz, DMSO-d_6)
120
100
80
60
40
20
0
-20
-40
-60
-80
-100
-120
-140
-160
-180
-200
-220
f1 (ppm)

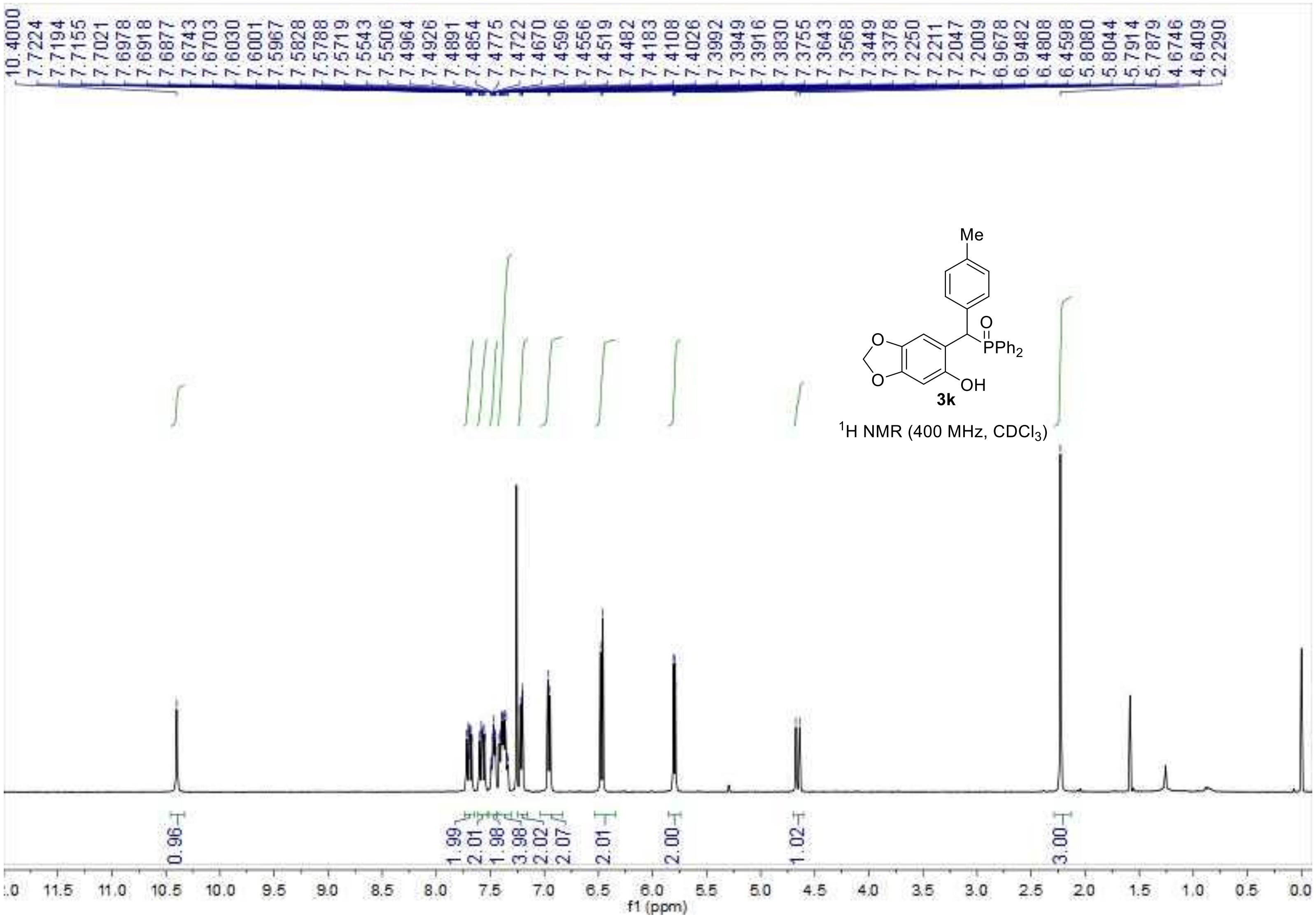

10.4000
7.7224
7.7194
7.7155
7.7021
7.6978
7.6918
7.6877
7.6743
7.6703
7.6030
7.6001
7.5967
7.5828
7.5788
7.5719
7.5543
7.5506
7.4964
7.4926
7.4891
7.4854
7.4775
7.4722
7.4670
7.4596
7.4556
7.4519
7.4482
7.4183
7.4108
7.4026
7.3992
7.3949
7.3916
7.3830
7.3755
7.3643
7.3568
7.3449
7.3378
7.2250
7.2211
7.2047
7.2009
6.9678
6.9482
6.4808
6.4598
5.8080
5.8044
5.7914
5.7879
4.6746
4.6409
2.2290
Me
O
PPh2
OH
3k
1H NMR (400 MHz, CDCl3)
0.96
1.99
2.01
1.98
3.98
2.02
2.07
2.01
2.00
1.02
3.00
f1 (ppm)

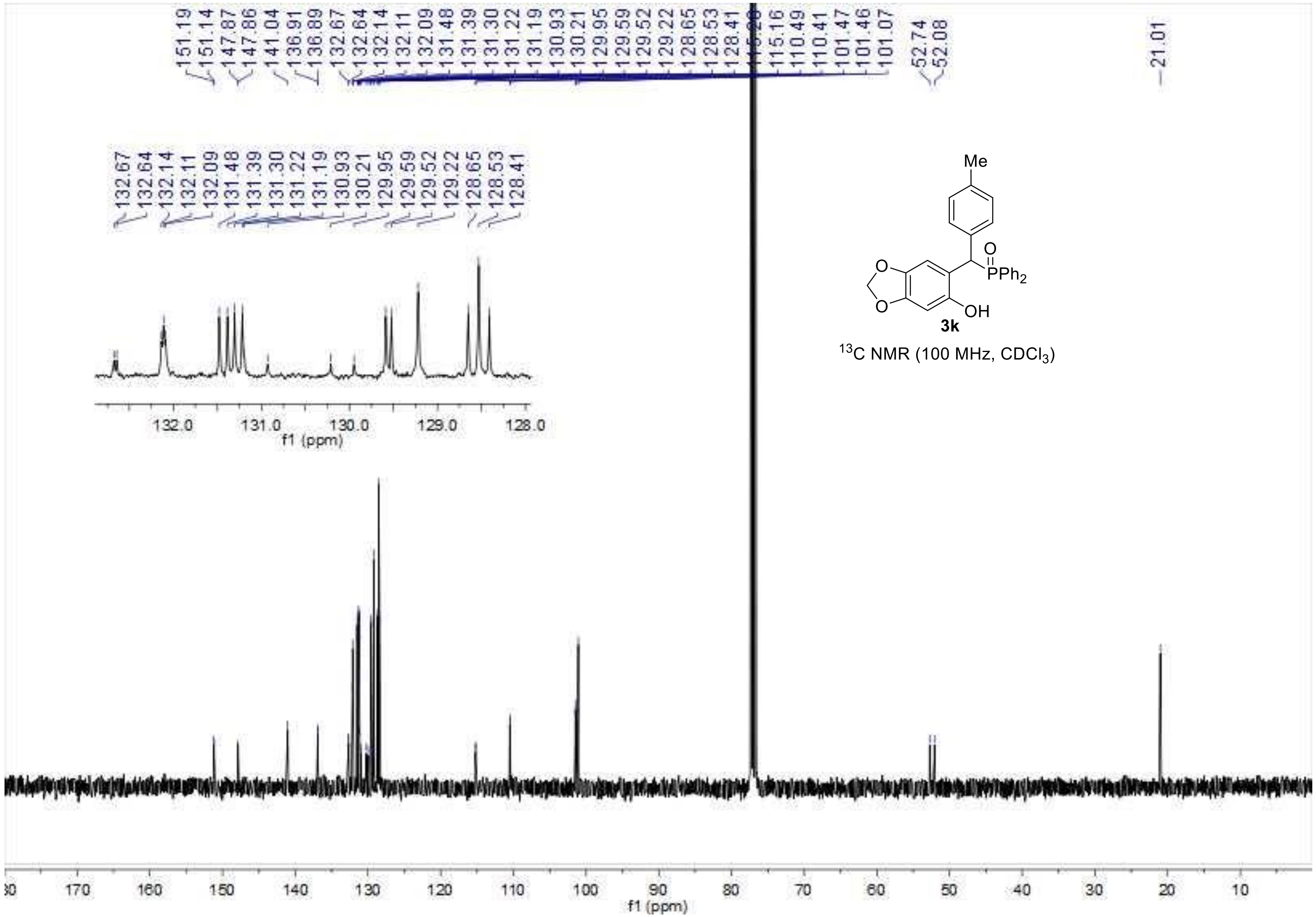

3k
13C NMR (100 MHz, CDCl3)
f1 (ppm)

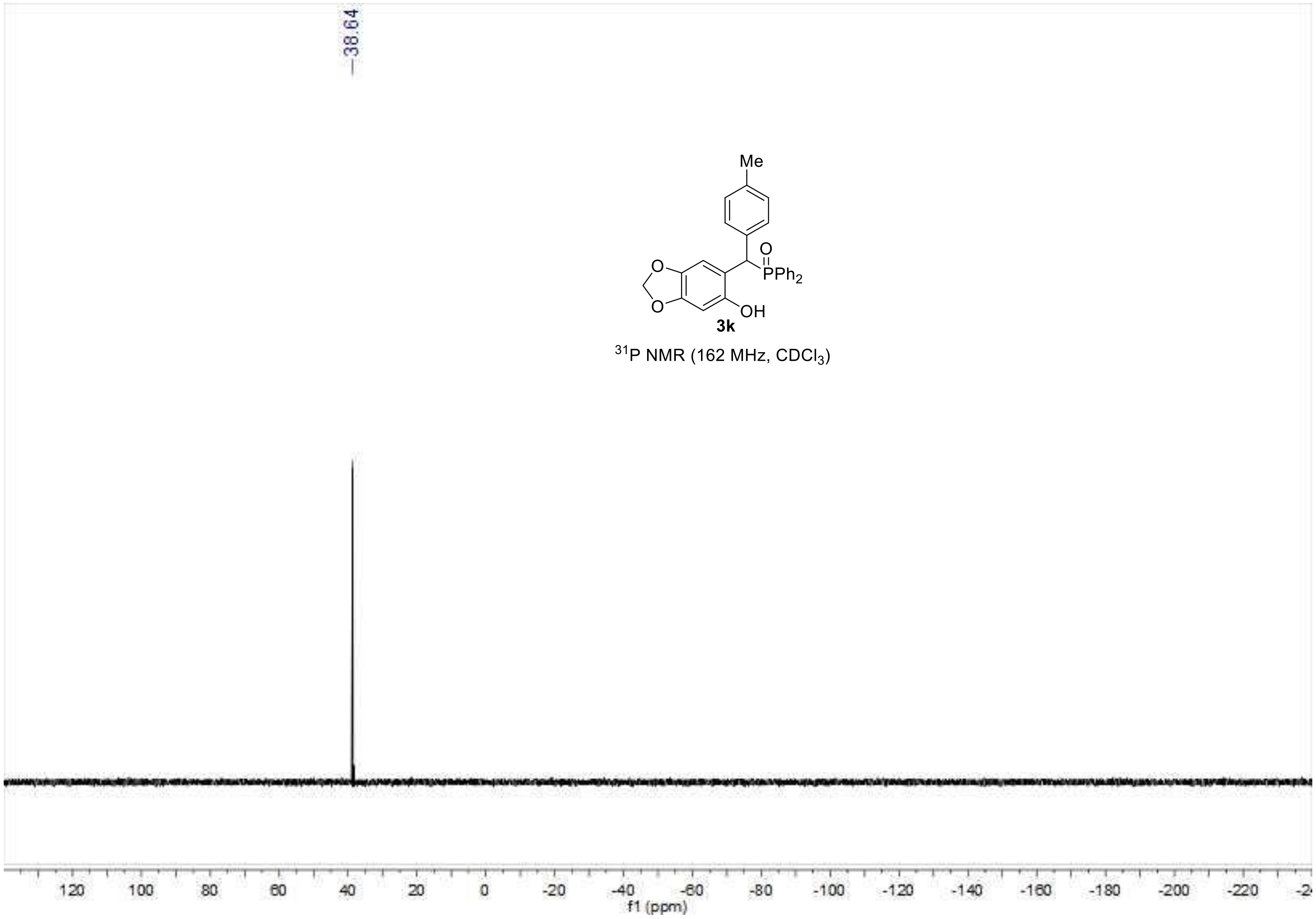

38.64
Me
O
PPh2
O
O
OH
3k
31P NMR (162 MHz, CDCl3)
120 100 80 60 40 20 0 -20 -40 -60 -80 -100 -120 -140 -160 -180 -200 -220
f1 (ppm)

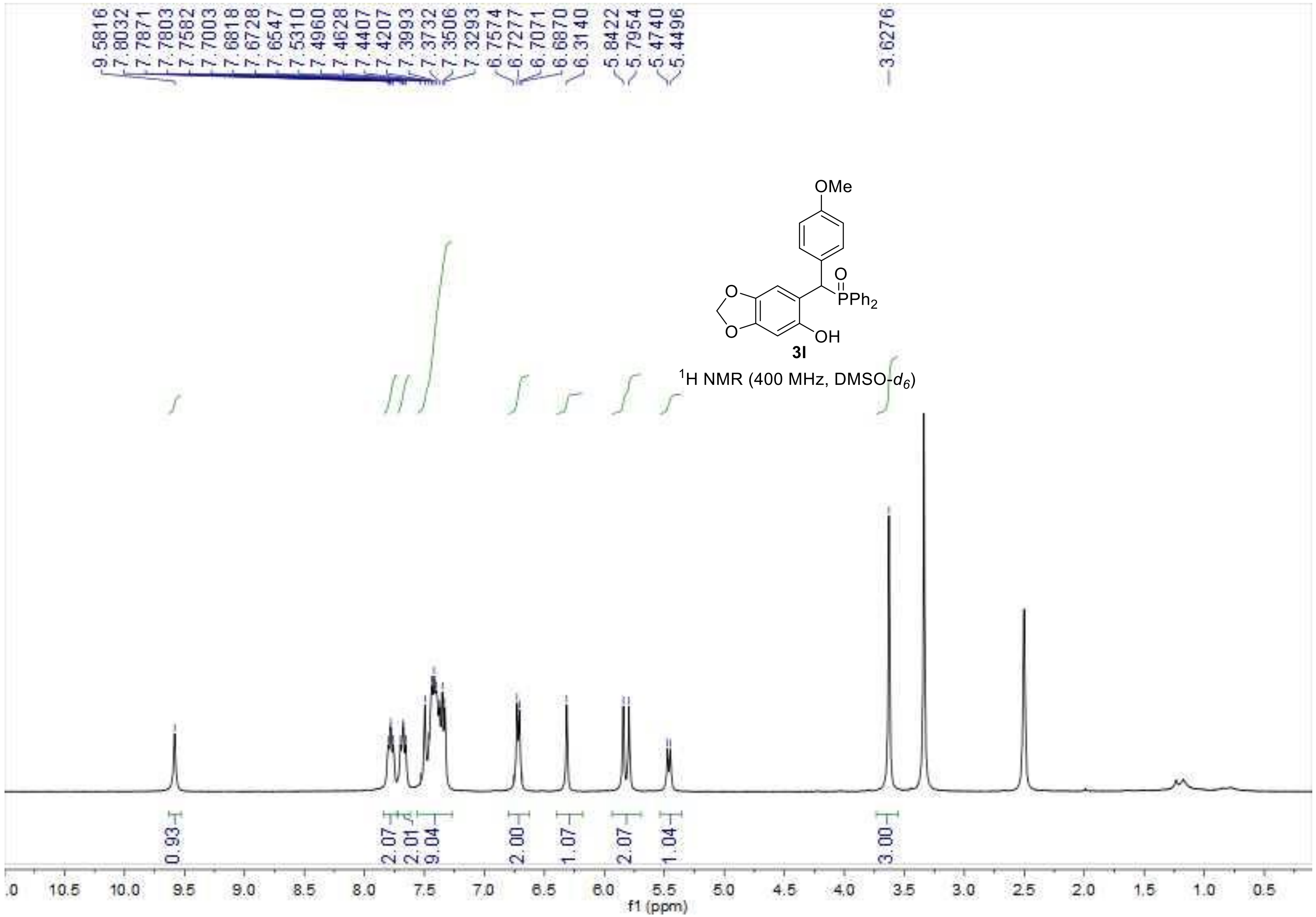

9.5816
7.8032
7.7871
7.7803
7.7582
7.7003
7.6818
7.6728
7.6547
7.5310
7.4960
7.4628
7.4407
7.4207
7.3993
7.3732
7.3506
7.3293
6.7574
6.7277
6.7071
6.6870
6.3140
5.8422
5.7954
5.4740
5.4496
3.6276
OMe
O
PPh2
O
O
OH
3l
1H NMR (400 MHz, DMSO-d6)
0.93
2.07
2.01
9.04
2.00
1.07
2.07
1.04
3.00
f1 (ppm)

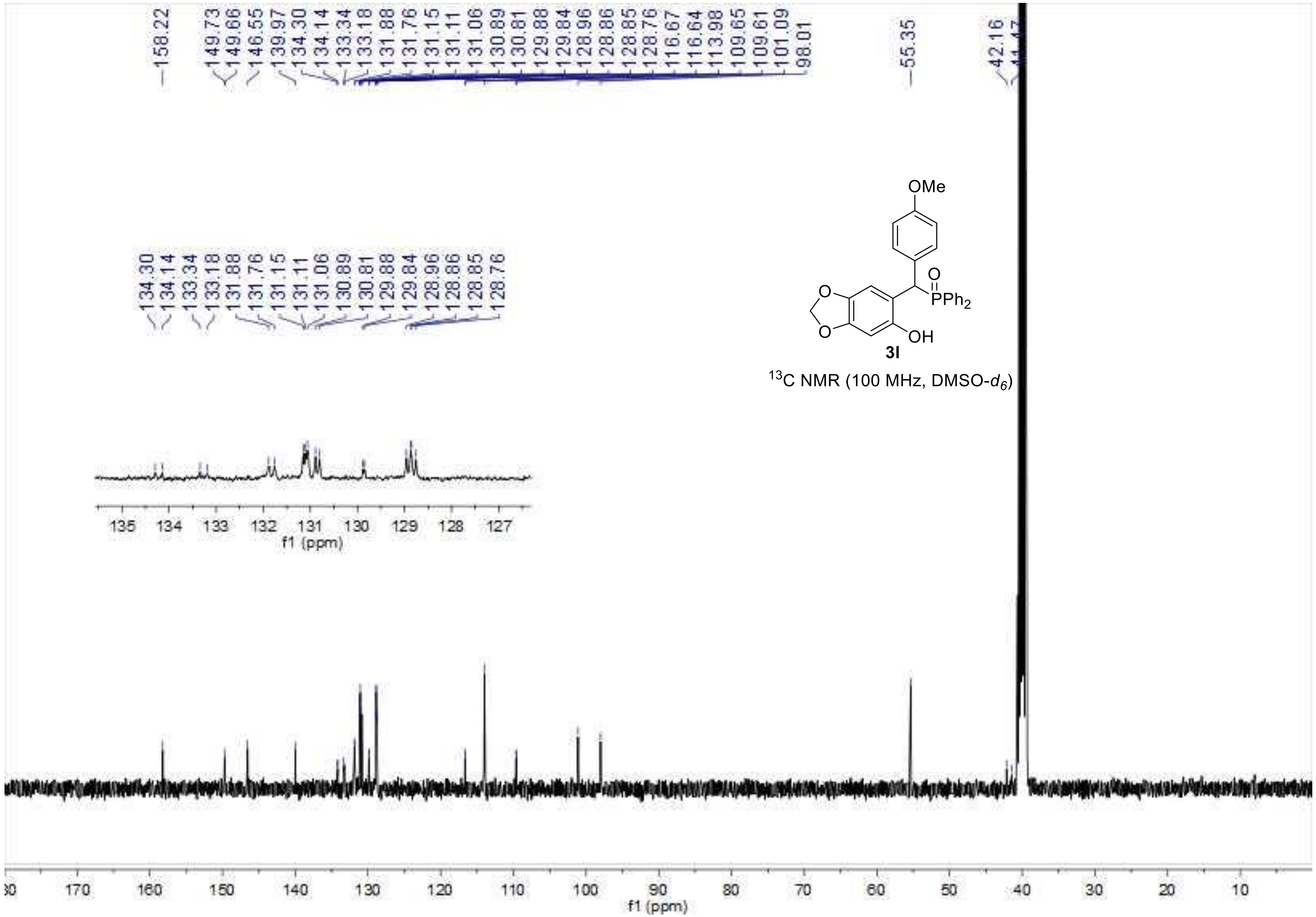

158.22
149.73
149.66
146.55
139.97
134.30
134.14
133.34
133.18
131.88
131.76
131.15
131.11
131.06
130.89
130.81
129.88
129.84
128.96
128.86
128.85
128.76
116.67
116.64
113.98
109.65
109.61
101.09
98.01
55.35
42.16
OMe
O
PPh2
OH
3l
^{13}C NMR (100 MHz, DMSO-d_6)
f1 (ppm)

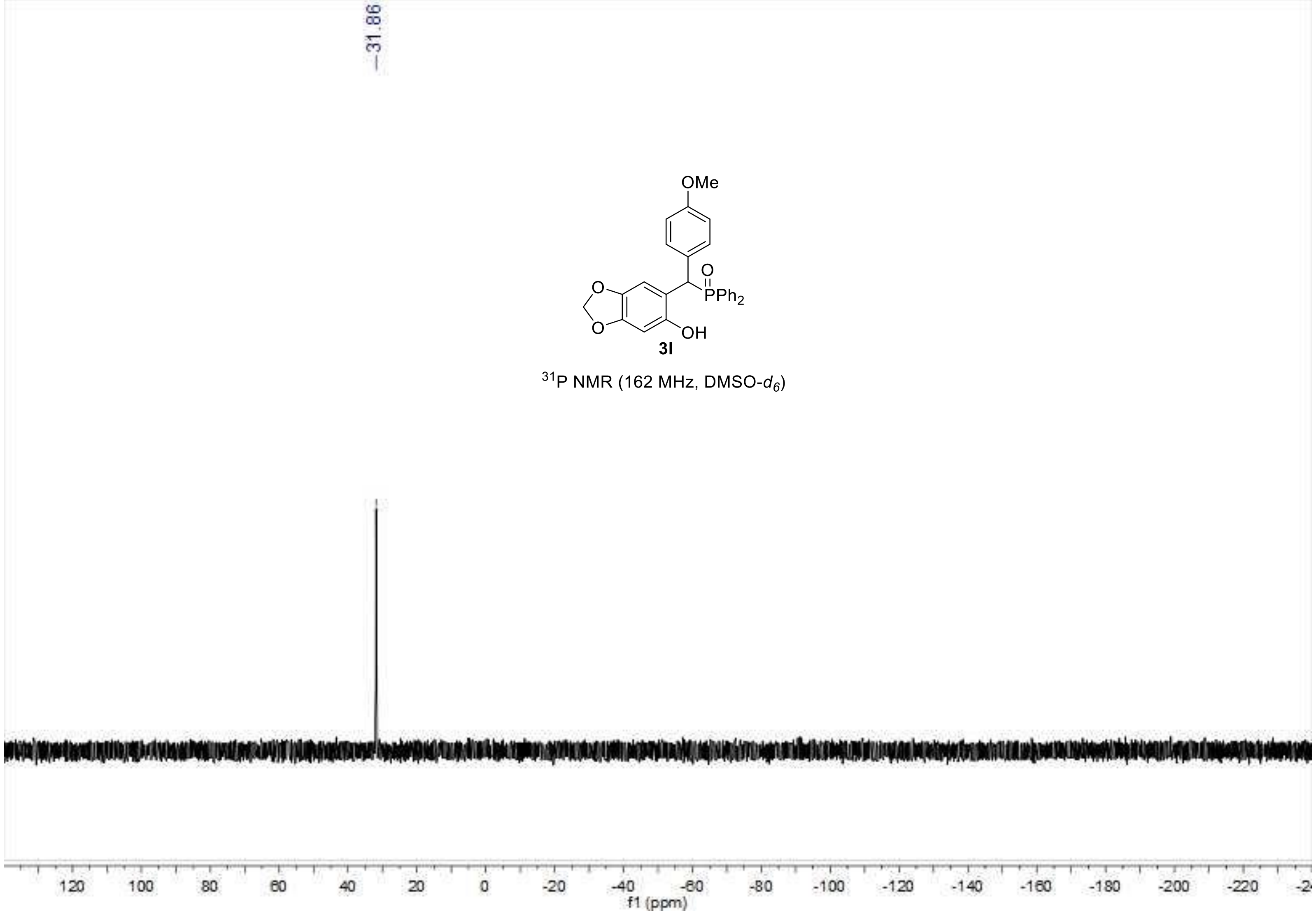

31.86
OMe
O
PPh2
O
O
OH
3l
31P NMR (162 MHz, DMSO-d6)
120 100 80 60 40 20 0 -20 -40 -60 -80 -100 -120 -140 -160 -180 -200 -220
f1 (ppm)

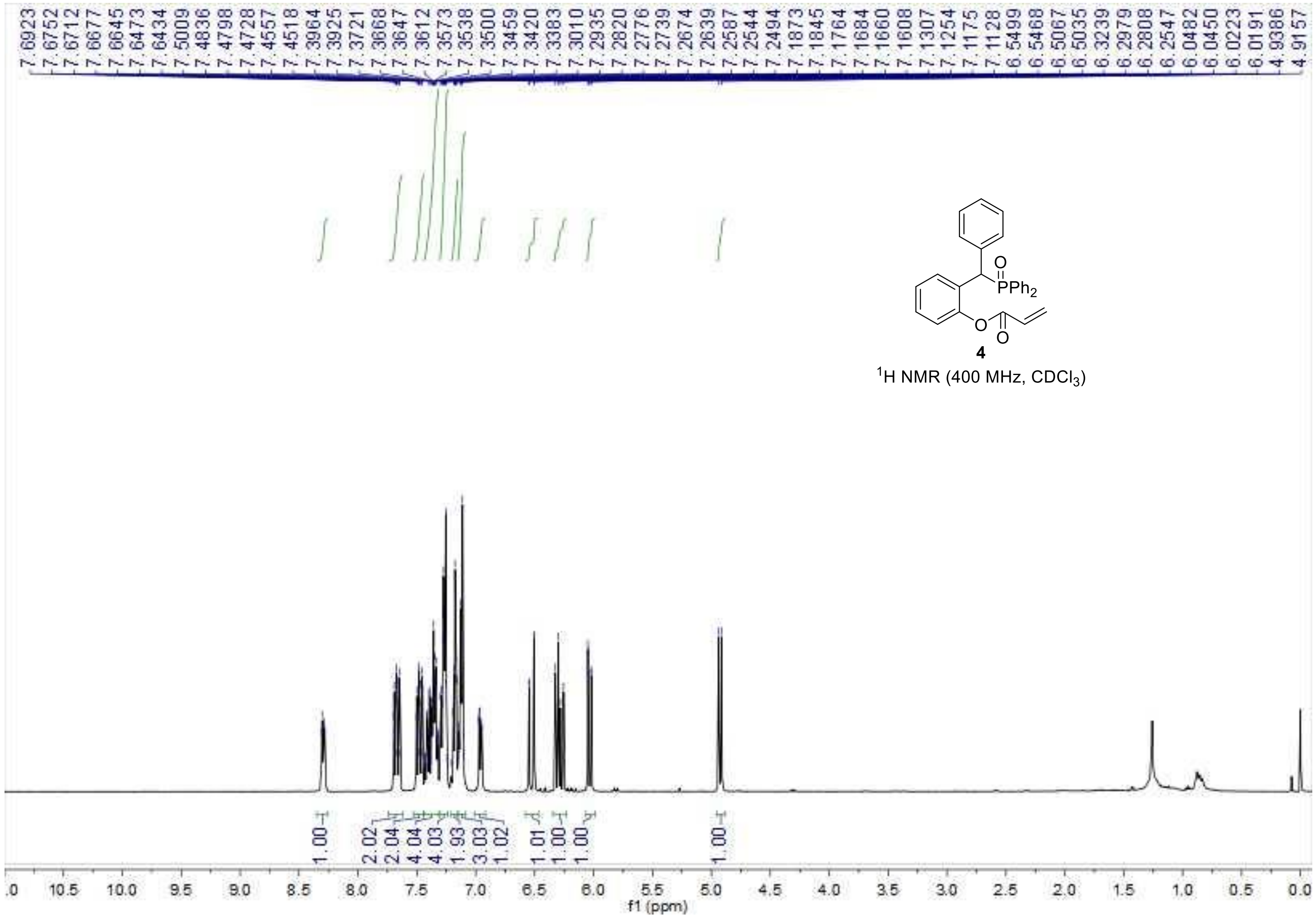
7.6923
7.6752
7.6712
7.6677
7.6645
7.6473
7.6434
7.5009
7.4836
7.4798
7.4728
7.4557
7.4518
7.3964
7.3925
7.3721
7.3668
7.3647
7.3612
7.3573
7.3538
7.3500
7.3459
7.3420
7.3383
7.3010
7.2935
7.2820
7.2776
7.2739
7.2674
7.2639
7.2587
7.2544
7.2494
7.1873
7.1845
7.1764
7.1684
7.1660
7.1608
7.1307
7.1254
7.1175
7.1128
6.5499
6.5468
6.5067
6.5035
6.3239
6.2979
6.2808
6.2547
6.0482
6.0450
6.0223
6.0191
4.9386
4.9157
1.00
2.02
2.04
4.04
4.03
1.93
3.03
1.02
1.01
1.00
1.00
1.00
PPh2
O
O
O
4
1H NMR (400 MHz, CDCl3)
f1 (ppm)

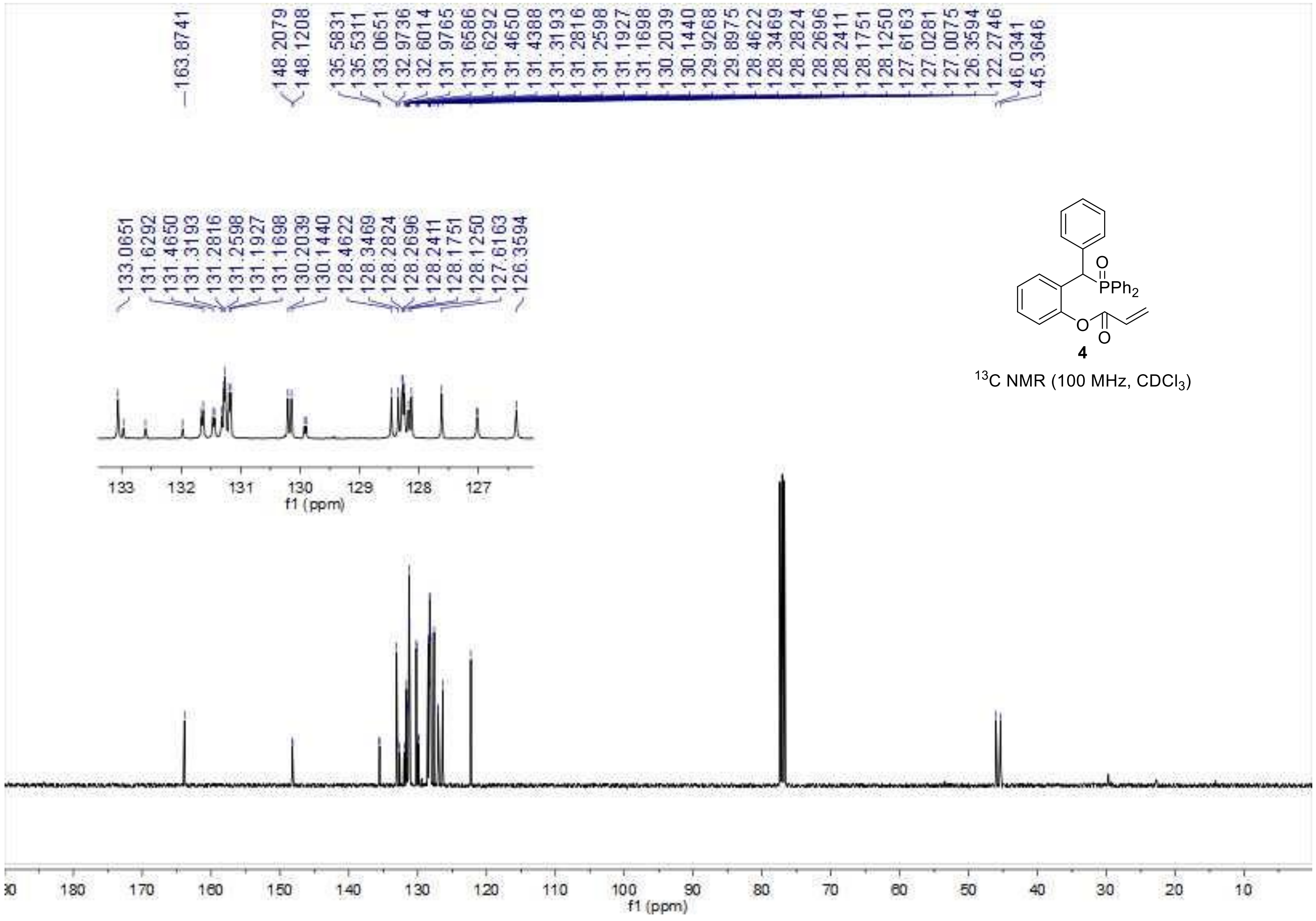

163.8741
148.2079
148.1208
135.5831
135.5311
133.0651
132.9736
132.6014
131.9765
131.6586
131.6292
131.4650
131.4388
131.3193
131.2816
131.2598
131.1927
131.1698
130.2039
130.1440
129.9268
129.8975
128.4622
128.3469
128.2824
128.2696
128.2411
128.1751
128.1250
127.6163
127.0281
127.0075
126.3594
122.2746
46.0341
45.3646
PPh2
O
O
O
4
13C NMR (100 MHz, CDCl3)
f1 (ppm)

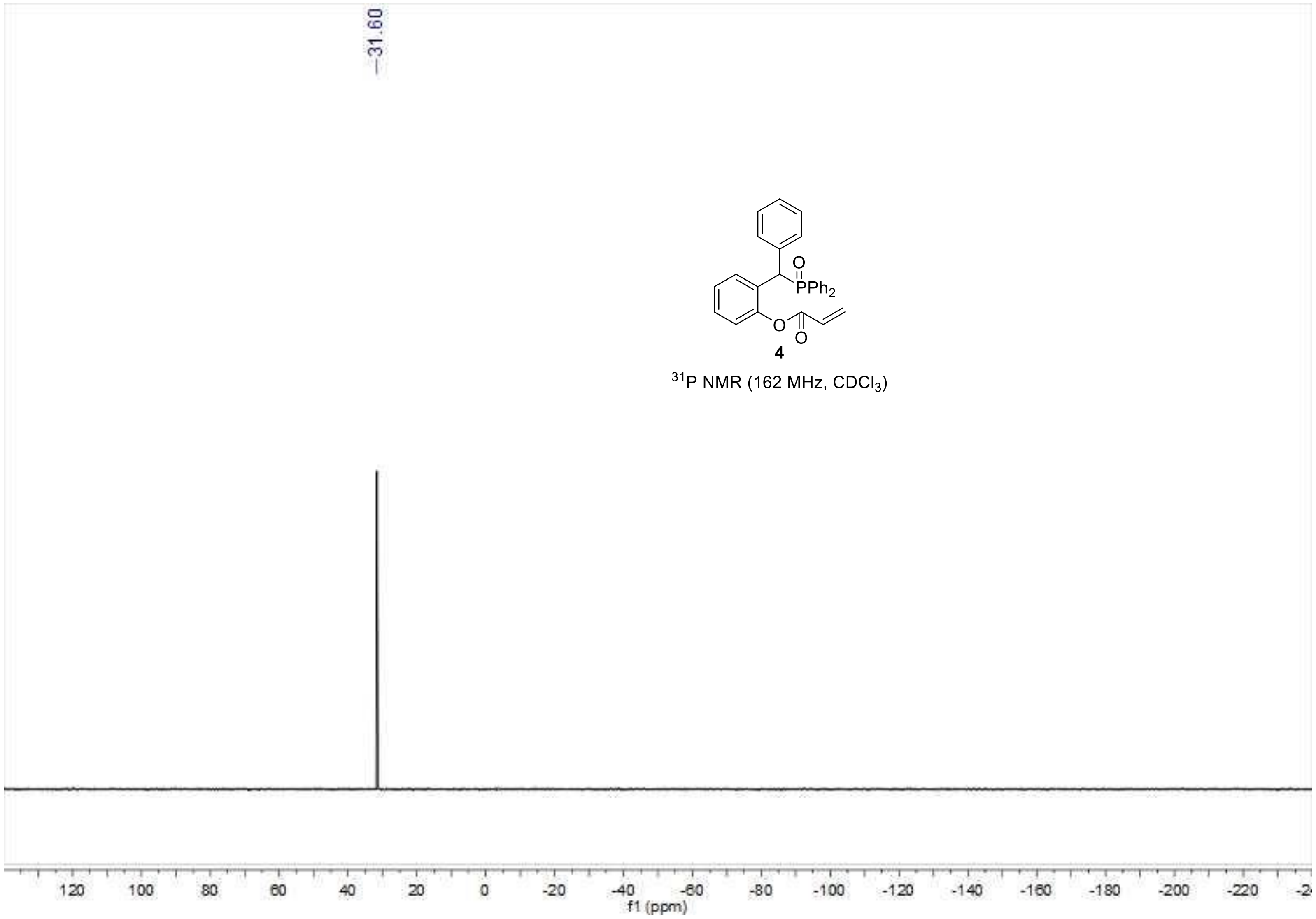
31.60
O
PPh2
O
O
4
31P NMR (162 MHz, CDCl3)
120 100 80 60 40 20 0 -20 -40 -60 -80 -100 -120 -140 -160 -180 -200 -220
f1 (ppm)
